# Integrated QSAR Models for Prediction of Serotonergic Activity: Machine Learning Unveiling Activity and Selectivity Patterns of Molecular Descriptors

**DOI:** 10.3390/pharmaceutics16030349

**Published:** 2024-03-01

**Authors:** Natalia Łapińska, Adam Pacławski, Jakub Szlęk, Aleksander Mendyk

**Affiliations:** 1 Department of Pharmaceutical Technology and Biopharmaceutics, Jagiellonian University Medical College, 30-688 Kraków, Poland; natalia.czub@doctoral.uj.edu.pl (N.Ł.); j.szlek@uj.edu.pl (J.S.); aleksander.mendyk@uj.edu.pl (A.M.); 2 Doctoral School of Medicinal and Health Sciences, Jagiellonian University Medical College, 31-530 Kraków, Poland; 3 Bioinformatics and In Silico Analysis Laboratory, Center for the Development of Therapies for Civilization and Age-Related Diseases (CDT-CARD), 8 Skawińska St., 31-066 Kraków, Poland

**Keywords:** serotonin receptors, molecular descriptors, Mordred, machine learning, statistical analysis, selectivity and activity

## Abstract

Understanding the features of compounds that determine their high serotonergic activity and selectivity for specific receptor subtypes represents a pivotal challenge in drug discovery, directly impacting the ability to minimize adverse events while maximizing therapeutic efficacy. Up to now, this process has been a puzzle and limited to a few serotonergic targets. One approach represented in the literature focuses on receptor structure whereas in this study, we followed another strategy by creating AI-based models capable of predicting serotonergic activity and selectivity based on ligands’ representation by molecular descriptors. Predictive models were developed using Automated Machine Learning provided by Mljar and later analyzed through the SHAP importance analysis, which allowed us to clarify the relationship between descriptors and the effect on activity and what features determine selective affinity for serotonin receptors. Through the experiments, it was possible to highlight the most important features of ligands based on highly efficient models. These features are discussed in this manuscript. The models are available in the additional modules of the SerotoninAI application called “Serotonergic activity” and “Selectivity”.

## 1. Introduction

Serotonin receptors are an important group of biological targets belonging mainly to G protein-coupled receptors (GPCRs). Among them, we can distinguish as many as 13 subtypes of receptors, namely 5-HT1A, 5-HT1B, 5-HT1D, 5-HT1E, 5-HT1F, 5-HT2A, 5-HT2B, 5-HT2C, 5-HT4, 5-HT5A, 5-HT5B, 5-HT6, and 5-HT7. The only instance of a receptor outside this group is the 5-HT3 receptor, which belongs to the group of ionotropic receptors. Serotonin receptors play a key role in various physiological functions as they are widely distributed in the nervous system and peripheral tissues. These receptors are currently extensively explored as targets in the drug discovery process (against migraine, depression, and schizophrenia or in treatment of nausea) [1].

Among the processes focusing on the discovery of new therapeutic molecules, there are two main streams: Structure-Based Drug Design and Ligand-Based Drug Design. The first approach includes molecular docking and virtual screening. One can find most of the structures of serotonin receptors on the UniProt platform [2]. The literature includes publications focusing on describing the structural and functional differences between serotonin receptors, including their localization in different brain areas [3,4,5,6,7]. There have been studies focusing on describing the relationship between descriptors and affinity or activity values for specific receptors. Bukhari et al. discussed the effect of PyDescriptors and PaDEL on the pKi values of ligands relative to serotonin 5-HT6 receptors. They developed QSAR (quantitative structure–activity relationship) models based on a database of more than 1200 molecules and applied molecular docking to visualize the potential effect of selected descriptors on ligand–receptor interactions [8]. In turn, the paper by Petković et al. presented datasets of 50 molecules with observed serotonin transporter (pIC50) inhibitory effects. They created QSAR models using Monte Carlo optimization on local graph invariants and descriptors based on SMILES notation, a genetic algorithm based on two-dimensional PaDEL descriptors [9]. 

This was based on a review of information by A. Sandri [10] in which the author conducted a thorough analysis of different approaches to drug discovery together with the number of approved molecules. According to this information, focusing solely on biological targets may have limited chances of success. However, it is worth noting that this opinion does not exclude the usefulness of methods based on biological targets and affinity. On the contrary, it may represent one of many insights into a comprehensive research process. At the outset, it can be emphasized that these methods are an important part of successful drug discovery in the future, albeit while taking into account other approaches such as those based on observable phenotypic effects.

In this article, the analysis concentrates on differences between active/inactive compounds in the serotonin system and serotonin receptors based on ligand characteristics, represented by molecular descriptors. Moreover, we want to obtain models of serotonergic activity and selective binding to a chosen serotonin receptor. To our best knowledge, this type of concept has not yet been developed for serotonin receptors. These research objectives were based on statistical methods and machine learning with an emphasis on Automated Machine Learning with SHapley Additive exPlanations analysis (SHAP analysis). The results obtained provide a basis for the search for molecule features important for active and selective interaction with serotonin receptors. Perhaps these descriptors would be analogous to Lipinski’s features indicating ‘druglikeness’ and, in our case, will be features indicating serotonergic activity and selectivity for selected serotonin receptors [11]. This selectivity may have a positive effect by knowingly reducing the occurrence of adverse effects. In the case of the serotonergic system, adverse events are varied; for example, 5-HT2A receptor activation can lead to psychedelic effects whereas 5-HT3 receptor activation can lead to nausea. 

## 2. Materials and Methods

### 2.1. Databases

The databases were prepared in September 2023 based on two leading repositories of compounds—ZINC and ChEMBL [12,13]. Data regarding 5-HT1A, 5-HT1B, 5-HT1D, 5-HT2A, 5-HT2B, 5-HT2C, 5-HT3, 5-HT4, 5-HT5A, 5-HT6, and 5-HT7 receptors were acquired for which the pKi values (negative logarithm from the inhibition constant) were known, indicating the affinity of the molecule to the selected receptor. In the data cleaning process, the first step was to remove duplicate molecules in the ZINC and ChEMBL databases separately. Later, the two databases were merged and then checked for duplicates once again. If the difference in pKi values for the same molecule between the ZINC and ChEMBL databases was greater than 0.1, that ligand was removed from the database. When the difference was less than or equal to 0.1, ZINC molecule was selected, according to methods described in previously published article [14]. 

The next step involved merging these databases. At the same time, compounds that were present in more than one serotonin receptor’s database were removed from them. As a result, molecules that were unique to only one subtype of serotonin receptor remained in the database. This step was performed to avoid overlapping activities when one ligand could interact with several serotonin receptors, i.e., 5-HT1B, 5-HT1D, and 5-HT6 subtypes. The purpose of this model created was to achieve selectivity of molecules rather than to detect all available serotonin receptor affinity possibilities. 

Our analysis was based on two classification models. The first was designed to predict highly active compounds for serotonergic receptors/system without selectivity to the specific subtypes. The cutoff point for active/inactive compounds was set at pKi = 7, approximately 100 nM, considered an effective concentration. For the second model, which aimed to identify compounds with selective affinity to serotonin receptors, only a fraction of molecules that proved to be active compounds (pKi ≥ 7) were used. In each case, we randomly divided datasets into training and test sets (80:20) [15].

### 2.2. Molecular Descriptors

Mordred [16] descriptors were used for all calculations. The input vector is composed of over 1600 two dimensional descriptors. Mordred provides a variety of molecular features, covering aspects such as 2D structure, chemical properties, topological indices, and number of atoms and bonds and characterizing physicochemical features such as polarizability or molecule size, which enables a comprehensive molecular analysis from the perspective of various parameters. We obtained molecular 2D descriptors with Mordred (version 1.2.0) using Python script according to authors’ guidelines from GitHub platform [17]. The database was pre-processed, involving the imputation of missing values with the average value of the relevant column and eliminating constant columns.

### 2.3. Metrics

For binary and multiclass (eleven classes) classification tasks, the main metrics used were average accuracy, precision, recall, Matthews correlation coefficient (MCC), and F1 score (Equations (1)–(5)). Moreover, experiment results are presented in confusion matrices.

(1) $$accuracy=\frac{TP+TN}{TP+TN+FP+FN}$$

(2) $$precision=\frac{TP}{TP+FP}$$

(3) $$recall=\frac{TP}{TP+FN}$$

(4) $$MCC=\frac{TP\times TN-FP\times FN}{\sqrt{\left(TP+FP)(TP+FN)(TN+FP)(TN+FN\right)}}$$

Here, TP = true positive, TN = true negative, FP = false positive, and FN = false negative.

(5) $$F1=\frac{2\times precision\times recall}{precision+recall}$$

### 2.4. Binary Classification for Serotonergic Activity

The first part of the computational experiment was the development and analysis of binary classification model. We assigned active compounds to class 1 (pKi value greater than or equal to 7) and inactive compounds to class 0. For model creation, Mljar tool was used in mode ‘classification’ [18]. The model was built according to 10-fold cross-validation executed on 80% of the dataset (train set), and its performance was evaluated on the remaining 20%. 

### 2.5. Multiclass Classification for Compounds with Selective Affinity to Serotonin Receptors

Further research was based on multiclass classification model. For this purpose, we used Mljar automated machine learning tool [18]. It does not have a direct multiclass classification mode, so in our case, we created a regression model with additional if–then rules to assign predicted values to appropriate classes. All transformations are presented in Table 1. 

Serotonin receptors were assigned the following numbers: 5-HT1A—1, 5-HT1B—2, 5-HT1D—3, 5-HT2A—4. 5-HT2B—5, 5-HT2C—6, 5-HT3—7, 5-HT4—8, 5-HT5A—9, 5-HT6—10, and 5-HT7—11. The MLjar-generated model was developed using 10-fold cross-validation method on 80% of the dataset and tested on the remaining 20%. 

### 2.6. Seeking Differentiating Descriptors

For databases of 11 serotonin receptors’ active molecules, we conducted analysis while creating multiclass classification models to find descriptors that differentiated these receptors with a ligand-based attitude. Moreover, for the best Mljar multiclass model, we conducted SHAP analysis. Furthermore, SHAP values give us information on the influence of particular descriptors on selective activity for serotonin receptors. On the other hand, we applied a classical statistical approach to this challenge by looking for statistically significant differences in descriptors representing ligands characterized by selectivity to particular serotonin receptors.

#### 2.6.1. SHAP Analysis

SHAP (SHapley Additive exPlanations) is a method for explaining complex AI/ML models based on the Shapley value concept introduced by Lloyd Shapley in 1952 [19]. Shapley, a Nobel laureate in Economic Sciences in 2012 for his contributions to game theory, focused on fairly distributing rewards among collaborating players in a cooperative game. The Shapley value, ensuring fairness by allocating the reward based on each player’s marginal contribution, provides a unique solution for dividing rewards among team members. In predictive modeling, SHAP analysis assesses the incremental contribution of each input variable to the model’s predicted outcome [20]. This research employed SHAP analysis in Python, utilizing a framework developed by J. Szlęk [21], augmented with a wrapper for the Mljar package to investigate the overall influence of each variable on the final prediction, considering both magnitude and direction. 

#### 2.6.2. Statistical Methods

**Figure 1:**
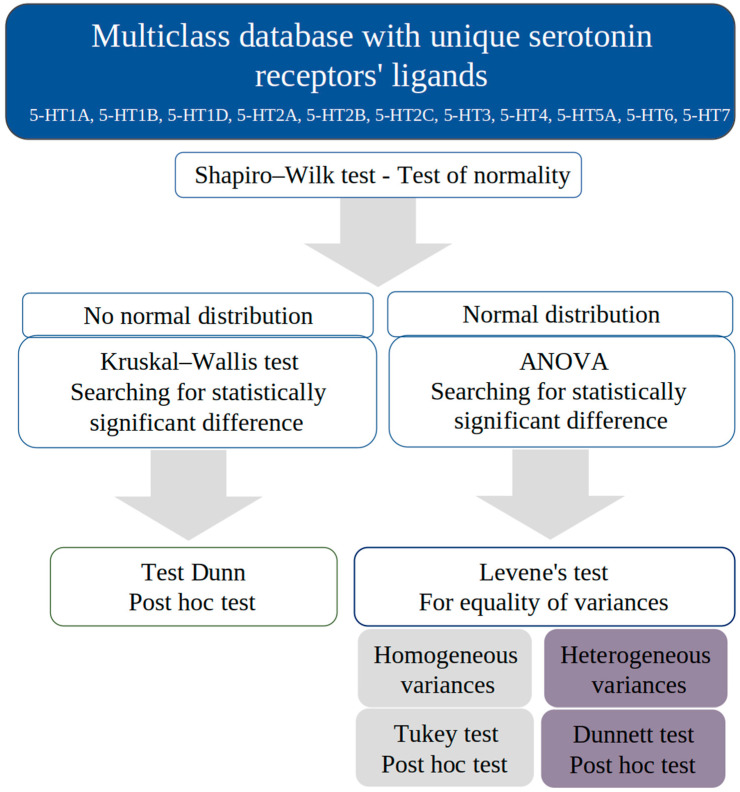
Scheme of ligands’ statistical analysis of serotonin receptors.

We conducted a comprehensive analysis to identify significant differences among serotonin receptors, using standard statistical tests based on entire databases. The statistical analysis was carried out using Python programming language and libraries containing relevant statistical tests: scipy (version 1.7.3) [22], statsmodels (version 0.13.5) [23], and scikit-posthocs (version 0.7.0) [24]. The script created and used is available on the GitHub platform (https://github.com/nczub/Statistical_analysis, accessed on 1 January 2024). Our inference was based on the significance level of α = 0.05. The steps of statistical analysis are presented in Figure 1. First, among all serotonin receptor groups, we analyzed the distribution of the variables, using the Shapiro–Wilk test to evaluate the normality of the distribution. 

If we found normality of distribution for a given descriptor in each serotonin receptor group, we conducted an analysis of variance (ANOVA). Once we were informed of statistically significant differences, we checked the type of variance using the Levene test to adjust the analysis to the appropriate post hoc test. For homogeneous variances, we used Tukey’s test, while for heterogeneous variances, we used Dunnett’s test to locate significant differences between serotonin receptors on selected descriptors. 

In the absence of normality in the distribution of variables, we used the Kruskal–Wallis test. Analogous to the ANOVA test, it helped us identify descriptors that differentiated selected serotonin receptors based on ligands’ descriptors. Dunn’s test was used as a post hoc test to further locate significant differences between serotonin receptors. Our analysis was a comprehensive approach to understanding the subtle differences between serotonin receptors, which may be crucial to understanding their functions and interactions with ligands. 

## 3. Results

### 3.1. Databases

**Figure 2:**
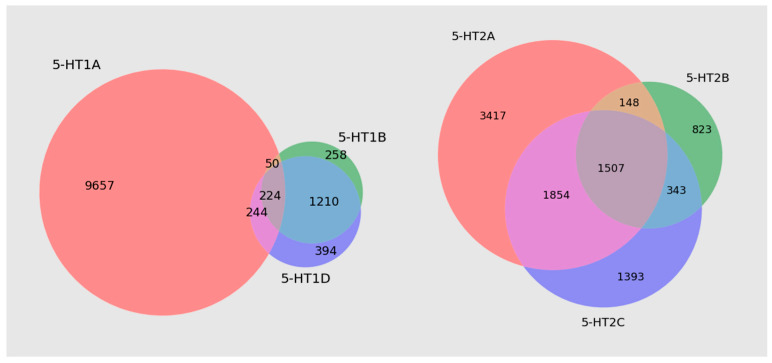
Venn diagrams for the subfamily of 5-HT1 and 5-HT2 serotonin receptors were constructed using the entire preliminary database.

The preliminary serotonin receptor databases contained molecules that appeared more than once. In the Venn diagrams below (Figure 2), we have shown the degree of molecular overlap using examples for 5-HT1 and 5-HT2 subfamilies. 

In the dataset preparation process, we obtained 40,542 records. Based on these data, we removed ligands that occurred more than once in the database. In the end, there were 18,967 unique records in the database. Next, based on adopted methods, data collection was divided into active (11,885) and inactive (7082) compounds towards serotonin receptors, and this was the basis for the binary classification model. In the multiclass classification problem, we used only active ligands; the distribution of serotonin receptors ligands is presented in Table 2. The dataset includes a variety of record counts for different receptor subtypes, ranging from 76 for 5-HT1B to 5422 for 5-HT1A. To accurately represent the analyzed problem, we chose not to balance the classes when developing our multiclass classification model. This method ensured that the development of our model and the results it produced were true reflections of the data’s real-world distribution and complexity. It also helped avoid additional biases that could have affected the model’s analysis later on. The distribution of classes was preserved when dividing the dataset into training and test sets. 

### 3.2. Statistical Approach

**Figure 3:**
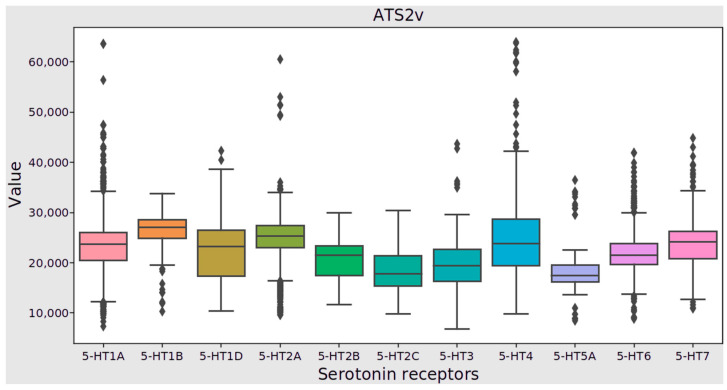
Box plot for serotonin receptors for ATS2v Mordred descriptor.

During statistical analysis, 1306 descriptors were assessed against 11 groups of data describing unique serotonin receptor ligands. Ten descriptors (NddC, NssNH2, NsssNH, SddC, SssNH2, SsssNH, n12HRing, n12AHRing, n7FHRing, n7FAHRing) had a constant value for all groups. None of the descriptors satisfied the assumption of normal distribution; therefore, the Kruskal–Wallis test was employed to assess statistically significant differences. As a result, 1268 ligand features exhibited statistically significant differences. In order to identify the specific pairs of serotonin receptors between which these differences occurred, a Dunn’s test (post hoc) was performed. The results indicated that no single descriptor was differentiated among all 11 serotonin receptor types. The highest number of significantly different descriptors was observed between the 5-HT2A and 5-HT6 receptors (1136) while the lowest number was found between the 5-HT2C and 5-HT5A receptors (583). In the Supplementary Materials, the results of Dunn’s tests for all pairs of serotonin receptors (Supplementary S1), as well as graphs of the distribution of the values of 31 selected descriptors (Supplementary S2) that exhibited the most substantial differences between serotonin receptors, can be found. Figure 3 illustrates the distribution of values for serotonin receptors using the ATS2v descriptor as an example. Notably, no statistically significant differences were found between the 5-HT1A–5-HT1D, 5-HT1B–5-HT2A, 5-HT2C–5-HT3, 5-HT2C–5-HT5A, and 5-HT4–5-HT7 receptors. These results indicate that there are significant differences between certain pairs of serotonin receptors. However, the statistical analysis of ligand descriptors alone may not suffice for identifying the selectivity of a given molecule towards serotonin receptors. 

### 3.3. Binary Classification Model

**Figure 4:**
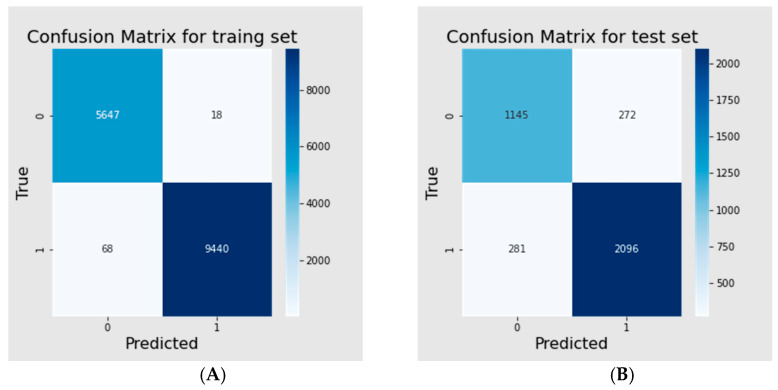
Confusion matrices for training (**A**) and test (**B**) sets of active/inactive model.

From over 1600 models, we have selected the best classification ensemble model based on the F1 score value (5 × Xgboost, 6 × LightGBM, 2 × Neural Networks, 2 × CatBoost). The results are shown in Table 3 and confusion matrices (Figure 4). Based on the results, almost 100% and 85% of the training and test sets, respectively were assigned correctly. The model incorrectly predicted only 86 molecules, representing 0.57% of the data. Most of these (68 molecules) were active molecules predicted to be inactive. For the test set, for the binary classification, 281 active molecules and 272 inactive molecules were incorrectly assigned to the observed class, representing, in total, 15% of the test data.

SHAP analysis outlined 37 of the most important descriptors that, in total, represented 50% of the influence on the model assignment of each molecule to the active or inactive class. They are presented in Table 4 with short descriptions. The Supplementary Materials include information on descriptor value ranges for the entire set for active and inactive molecules (value ranges—Supplementary S3 and radial plots—Supplementary S4). 

### 3.4. Multiclass Classification Models

**Figure 5:**
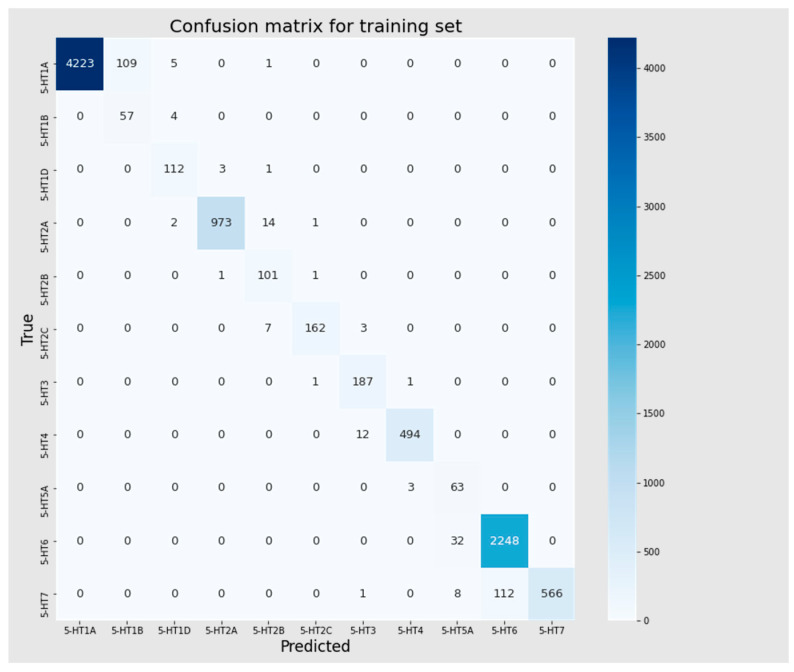
Confusion matrix for training set.

**Figure 6:**
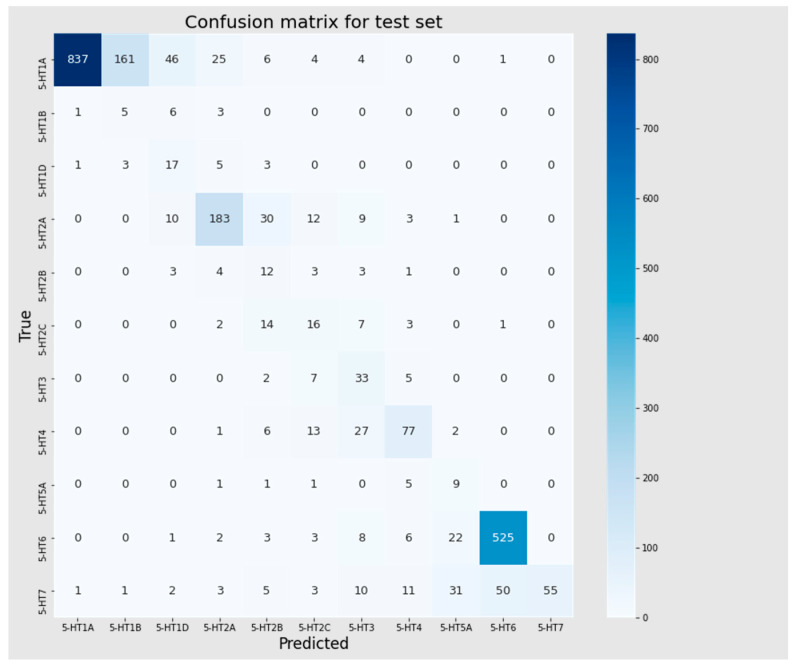
Confusion matrix for test set.

In the Mljar model, over 96% and 74% of the training and test sets, respectively were assigned correctly. Mispredictions for the training set mainly involved the 5-HT1A receptor, predicted as 5-HT1B (109 cases), and the 5-HT7 receptor, predicted as 5-HT6 (112 cases). These instances of misclassification of neighboring classes might have been attributed to the cutoff values. A closer look revealed that for only seventeen compounds, the predicted difference was not related to a neighboring class. Among them, the most common were 5-HT7 receptor ligands predicted as 5-HT5A receptors (eight cases) and 5-HT1A receptor ligands predicted as 5-HT1D (five cases). For the test set, the model assigned 608 ligands to the incorrect receptor subtype. Sixty percent (60%) of the cases were related to a neighboring class, and similarly to the training set; these errors mainly affected the 5-HT1A (predicted as 5-HT1B) and 5-HT7 (predicted as 5-HT6) receptors. Below, we present classification metrics of the best model selected based on the F1 score (Table 5) and confusion matrices (Figure 5 and Figure 6) for these sets. 

**Figure 7:**
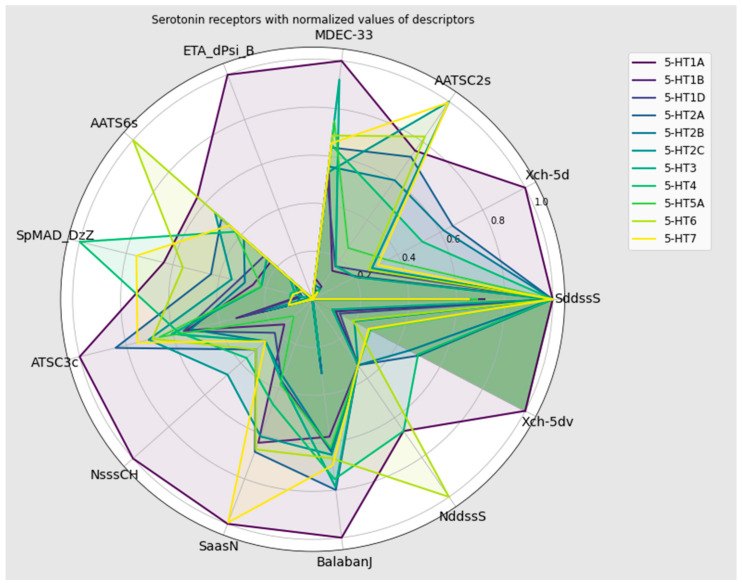
Radial plot of the most important descriptors for serotonin receptors (Min–Max normalization).

SHAP analysis proved 13 of the most important descriptors for selectivity towards serotonin receptors. They are SddssS, Xch-5d, AATSC2s, MDEC-33, ETA_dPsi_B, AATS6s, SpMAD_DzZ, ATSC3c, NsssCH, SaasN, BalabanJ, NddssS, and Xch-5dv. Their descriptions are provided in Table 6. Figure 7 presents the range of normalized (Min–Max) values for all serotonin receptors. The overlapping areas of values show, similar to the results of statistical tests, that there was no single descriptor whose values determined selective activity against the serotonin receptor. In the case of the Mljar model, it is important to notice that simple differences between serotonin receptors in the manner of specific descriptors are not easily perceived. This is due to the fact that the model is more complex. For details on the ranges of selected descriptors and the radial graph for individual serotonin receptors, see the Supplementary Materials (Supplementary S5 and Supplementary S6). 

## 4. Discussion

The findings presented in this article indicate that a single descriptor alone may not clearly differentiate the presence or absence of serotonergic activity or demonstrate selectivity towards serotonin receptors. Despite this observation, the statistical analysis results reveal a possibility to highlight a group of descriptors to collectively establish rules for determining activity and selectivity. These insights provide a basis for the further exploration and understanding of the relationships within serotonergic receptors. 

The binary classification model highlights the substantial importance of the features associated with both the descriptor groups (ATSC, GATS, JGI, GGI, Kier, MATS, PEOE_VSA, SlogP, VSA_Estate) as well as structure elements (aasC, aaaC, ssO, sssN, FRing, nBase). Moreover, the selectivity model for the 11 serotonin receptors shows the following groups of descriptors, including ATS, BlabanJ, Xch; those related to the number of structure elements (ddssS, sssCH, aasN); and those associated with the distance between atoms (MDEC-33). 

In both models, ATS Mordred descriptors are present. The AATSC features represent the average-centered autocorrelation of the topological structure (Moreau–Broto autocorrelation descriptor), defined as AATSk = ATSk/Δk, where Δk is the number of vertex pairs at an order equal to k. The ATSC descriptors represent a way of measuring the similarity or correlation between different atoms in a molecule based on their properties and distances. This involves calculating the average-centered autocorrelation of the molecule’s topological structure, where the topological structure refers to the arrangement of atoms and bonds in the molecule. This method helps capture important information about the molecular structure for further analysis in a simpler form [25]. Moreover, both models use descriptors representing spectral mean absolute deviation from the Barysz matrix (SpMAD_Dzare, SpMAD_DzZ). Another group of descriptors present in the models related to serotonergic activity and selectivity comprises those related to Chi descriptors. In the case of the binary model, these are AXp-7dv (valence-electron-weighted Chi path) and Xch-7dv (seven-ordered valence-electron-weighted Chi chain), and in the selectivity model they are Xc-5dv (five-ordered valence electron-weighted Chi chain) and Xch-5d (five-ordered sigma-electron-weighted Chi chain) [26]. 

**Figure 8:**
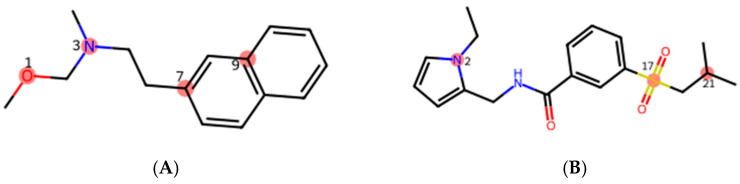
Specific atoms assigned to chemical structure; atoms selected in binary and multiclass classification models. (**A**) Binary classification: 1—ssO, 3—sssN, 7—aasC, 9—aaaC. (**B**) Multiclass classification: 2—aasN, 17—ddssS, 21—sssCH.

For the binary classification of serotonergic activity, more descriptors were detected. The first group, GATS, stands for the Geary coefficient descriptor. Those features represent a set of molecular descriptors that describe the spatial distribution of atom or bond properties in a molecule. Specifically, GATS descriptors are a type of autocorrelation descriptor calculated based on the Geary autocorrelation function. Autocorrelation involves measuring the similarity or correlation of a property between different atoms or bonds at varying distances within a molecule. Secondly, both JGI and GGI are descriptors that fall under the category of topological charge descriptors. These descriptors capture information about the electronic distribution and charge-related properties of atoms within a molecule. Another group of descriptors present in the binary model is Kier. It stands for ‘Kappa Shape Index’ and measures the molecular shape based on specific atom paths. Furthermore, MATS (Moran autocorrelation descriptor) is presented by this equation: MATS_k_ = AATSC_k_/(1/A⋅∑w^2^_c_), where W is the atomic property vector. An important group of descriptors appearing only in the binary classification model is PEOE-VSA (partial equalization of orbital electronegativity of van der Waals surface area). PEOE is a method of calculating partial atomic charges in which a charge is transferred between bound atoms until equilibrium is reached. To ensure convergence, the quantity of charge transferred in each iteration is suppressed by an exponentially decreasing scale factor. PEOE charges depend only on the connectivity of the input structures: elements, formal charges, and bond orders. Also associated with the van der Waals area are the VSA_EState2 and VSA_Estate7 descriptors found in the binary model. These are MOE-type descriptors using EState and surface share indices. In the serotonergic activity model, there are single descriptors discussing the neighborhoods of the atoms (IC1—number of edges of the subgraph, ZMIC3—three-ordered Z-modified information content) or the shape of the molecule TopoShapeIndex (topological shape index). Specific types of atoms and surroundings are also distinguished—aasC, aaaC, ssO, sssN—and shown in Figure 8. Moreover, SHAP analysis distinguished the number of base groups (nBase) or the fused ring count (nFRing). In drug design, an important feature of molecules is the logP value, which is one of Lipinski’s rules for the lipophilicity of a molecule. For the binary model, there are two derivatives of the logP descriptor, namely the SLogP value—the Wildman–Crippen LogP and SlogP_VSA1—and MOE-type descriptors using the Wildman–Crippen LogP and surface contribution [25,26,27]. 

In the selectivity model, the descriptors describing the structure are BalabanJ and the Extended Topochemical Atom descriptor (ETA_dPsi_B). The first one is a graph index used to describe the structural features of a graph. It takes into account the number of nodes, edges, and connected components in the graph. The calculation involves the graph’s distance matrix and the circuit rank, providing a numerical value that characterizes the graph’s complexity [28]. The serotonin receptor prediction model focuses on individual atom types in a specific environment (descriptors ddssS, sssCH, aasN—shown in Figure 8). Additionally, it considers the edge of the molecular distance between two tertiary carbon atoms, represented by MDEC-33 [25,26,27]. 

**Figure 9:**
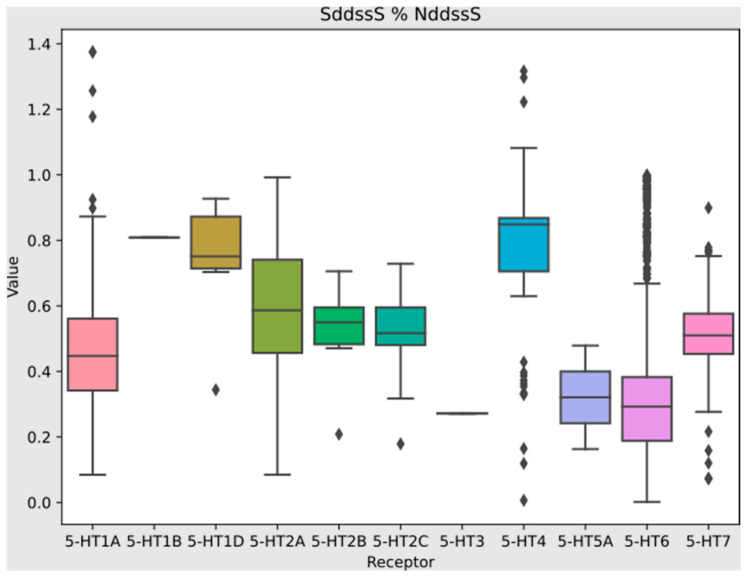
Box plots of derivatives of SddssS and NddssS Mordred descriptors for serotonin receptors.

Simultaneously examining these two models reveals the essential role of descriptors such as ATS, SpMAD, and Xch in predicting both serotonergic activity and selectivity. A comparative analysis underscores that for selectivity, information pertaining to the presence of sulfur in a specific arrangement within the molecular structure assumes greater importance. In contrast, in the serotonergic activity model, these elements do not emerge as descriptors responsible for 50% of the influence on predicting this feature. Descriptors NddssS and SddssS characterize the number and sum of electron states. The sum of SHAP values for those features constitutes almost 17% of the influence on selectivity towards serotonin receptors. In Figure 9, we present a modification of these two descriptors, which represents the remainder obtained by dividing the ‘NddssS’ variable by the ‘SddssS’ variable. This derivative differentiates over half of the serotonin receptors.

All the descriptors discussed above are the results of extensive modeling experiments carried out in this work. However, they were chosen from the predefined set of descriptors falling into the category of “2D” descriptors from the Mordred package [16]. Thus, our initial choice to limit the descriptor search space to 2D descriptors only was based on our previous experience with the numerical instability of 3D structure optimization methods resulting in the variability of 3D descriptors. In this view, we deny the added value of the descriptors’ sophistication [29] and trade it for the robustness and stability of a whole system.

Models of serotonergic activity, as well as the selectivity model, have become new extensions of SerotoninAI, a new web application related to serotonergic QSAR models [30] described in the article [31]. Applicability domain information was implemented in a form unified with other SerotoninAI modules. The ‘Serotonergic activity’ and ‘Selectivity’ sections provide radial charts of the ten most important descriptors. If their values for a tested compound are within the range for at least seven descriptors, the compound is in the applicability domain and predictions have a high probability of success.

Considerations related to the sulfur atom in relation to the pIC50 value for the 5-HT6 receptor appear in a study by Bukhari S.N.A. et al. Based on the QSAR model created, followed by a docking step to confirm the results obtained, among other things, a sulfur atom was determined that should be taken into account when optimizing the molecule for its effect on the 5-HT6 receptor [8].

In summary, this study demonstrated the importance of a comprehensive set of descriptors for understanding both serotonergic activity and receptor selectivity. The significant differences in the importance of the descriptors between the two models highlight the complex nature of predicting these pharmacological features.

## Figures and Tables

**Table 1:** Serotonin receptors, their corresponding numbers, and the associated range of predictions indicative of each receptor type.

Receptor	Number	Range of Predicted Values
5-HT1A	1	≤1.5
5-HT1B	2	1.5–2.5
5-HT1D	3	2.5–3.5
5-HT2A	4	3.5–4.5
5-HT2B	5	4.5–5.5
5-HT2C	6	5.5–6.5
5-HT3	7	6.5–7.5
5-HT4	8	7.5–8.5
5-HT5A	9	8.5–9.5
5-HT6	10	9.5–10.5
5-HT7	11	≥10.5

**Table 2:** Numbers of ligands for serotonin receptors that were used to develop a multiclass model.

Receptor	Quantity
5-HT1A	5422
5-HT1B	76
5-HT1D	145
5-HT2A	1238
5-HT2B	129
5-HT2C	215
5-HT3	236
5-HT4	632
5-HT5A	83
5-HT6	2850
5-HT7	859

**Table 3:** Results of binary classification for active and inactive compounds of serotonergic activity.

Dataset	Accuracy	Precision	Recall	F1	MCC
Training set	0.994	0.998	0.993	0.995	0.988
Test	0.854	0.885	0.882	0.883	0.689

**Table 4:** The most important Mordred descriptors for serotonergic activity, based on Mljar-SHAP approach [24].

No.	Descriptor	Description	av|SHAP Value|
1	nBase	basic group count	0.017
2	MATS1v	Moran coefficient of lag 1 weighted by vdw volume (van der Waals volume)	0.016
3	PEOE_VSA7	MOE Charge VSA Descriptor 7	0.016
4	SlogP_VSA1	MOE logP VSA Descriptor 1	0.014
5	AXp-7dv	Seven-ordered averaged Chi path weighted by valence electrons	0.014
6	PEOE_VSA9	MOE Charge VSA Descriptor 9	0.013
7	Xch-7dv	Seven-ordered Chi chain weighted by valence electrons	0.013
8	AATSC2dv	averaged and centered Moreau–Broto autocorrelation of lag 2 weighted by valence electrons	0.011
9	VSA_EState2	VSA EState Descriptor 2	0.011
10	ATSC6v	centered Moreau–Broto autocorrelation of lag 6 weighted by vdw volume	0.011
11	SLogP	Wildman–Crippen LogP	0.011
12	Kier2	kappa shape index 2	0.010
13	ATSC5d	centered Moreau–Broto autocorrelation of lag 5 weighted by sigma electrons	0.009
14	PEOE_VSA1	MOE Charge VSA Descriptor 1	0.009
15	JGI2	Two-ordered mean topological charge	0.009
16	SpMAD_Dzare	spectral mean absolute deviation from Barysz matrix weighted by Allred-Rochow EN	0.009
17	SaasC	sum of aasC	0.009
18	MATS1se	Moran coefficient of lag 1 weighted by sanderson EN	0.009
19	IC3	Three-ordered neighborhood information content	0.008
20	SaaaC	sum of aaaC	0.008
21	VSA_EState7	VSA EState Descriptor 7	0.008
22	ATSC7Z	centered Moreau–Broto autocorrelation of lag 7 weighted by atomic number	0.008
23	ZMIC3	Three-ordered Z-modified information content	0.007
24	JGI9	Nine-ordered mean topological charge	0.007
25	nFRing	fused ring count	0.007
26	Kier3	kappa shape index 3	0.007
27	SsssN	sum of sssN (>N-)	0.007
28	GATS4i	Geary coefficient of lag 4 weighted by ionization potential	0.007
29	PEOE_VSA6	MOE Charge VSA Descriptor 6	0.006
30	MAXaasC	max of aasC	0.006
31	GGI9	Nine-ordered raw topological charge	0.006
32	GATS6v	Geary coefficient of lag 6 weighted by vdw volume	0.006
33	GATS3i	Geary coefficient of lag 3 weighted by ionization potential	0.005
34	TopoShapeIndex	topological shape index	0.005
35	SssO	sum of ssO (-O-)	0.005
36	PEOE_VSA10	MOE Charge VSA Descriptor 10	0.005
37	GATS3v	Geary coefficient of lag 3 weighted by vdw volume	0.005

**Table 5:** Metrics for multiclass classification model.

Dataset	Accuracy	Precision	Recall	F1	MCC
Training set	0.966	0.976	0.966	0.969	0.953
Test	0.744	0.881	0.744	0.788	0.675

**Table 6:** The most important Mordred descriptors for selectivity towards serotonergic receptors, based on Mljar-SHAP approach.

No.	Descriptor	Description	av|SHAP Value|
1	SddssS	sum of ddssS (≥S≤)	0.731
2	Xch-5d	five-ordered Chi chain weighted by sigma electrons	0.341
3	AATSC2s	averaged and centered Moreau–Broto autocorrelation of lag 2 weighted by intrinsic state	0.272
4	MDEC-33	molecular distance edge between tertiary C and tertiary C	0.239
5	ETA_dPsi_B	ETA delta psi (type: B)	0.165
6	AATS6s	averaged Moreau–Broto autocorrelation of lag 6 weighted by intrinsic state	0.138
7	SpMAD_DzZ	spectral mean absolute deviation from Barysz matrix weighted by atomic number	0.132
8	ATSC3c	centered Moreau–Broto autocorrelation of lag 3 weighted by Gasteiger charge	0.095
9	NsssCH	number of sssCH (>CH-)	0.084
10	SaasN	sum of aasN	0.083
11	BalabanJ	Balaban’s J index	0.083
12	NddssS	number of ddssS (≥S≤)	0.081
13	Xch-5dv	five-ordered Chi chain weighted by valence electrons	0.077

## Data Availability

The data presented in this study are available on request from the corresponding author.

## Supplementary Materials

The following supporting information can be downloaded at: https://www.mdpi.com/article/10.3390/pharmaceutics16030349/s1, Supplementary S1: Results of Dunn test—*p* values for serotonin receptor pairs; Supplementary S2: Statistical approach summary—distribution of values of 31 selected descriptors; Supplementary S3: Value range of the most important descriptors for serotonergic activity model based on entire database; Supplementary S4: Values of molecular descriptors for serotonergic active and inactive molecules; Supplementary S5: Value range of the most important descriptors for selectivity model of serotonin receptors based on entire database; Supplementary S6: Radial plots for 13 most important descriptors for selectivity model of all serotonin receptors.
