# Supplementary material for: Integrated QSAR Models for Prediction of Serotonergic Activity: Machine Learning Unveiling Activity and Selectivity Patterns of Molecular Descriptors
Source: Pharmaceutics. 2024 Mar 1;16(3):349. doi: 10.3390/pharmaceutics16030349 (PMC10974160; doi:10.3390/pharmaceutics16030349)
Supplement: Supplementary file 1 [file pharmaceutics-16-00349-s001.zip › Supplementary S2.pdf]

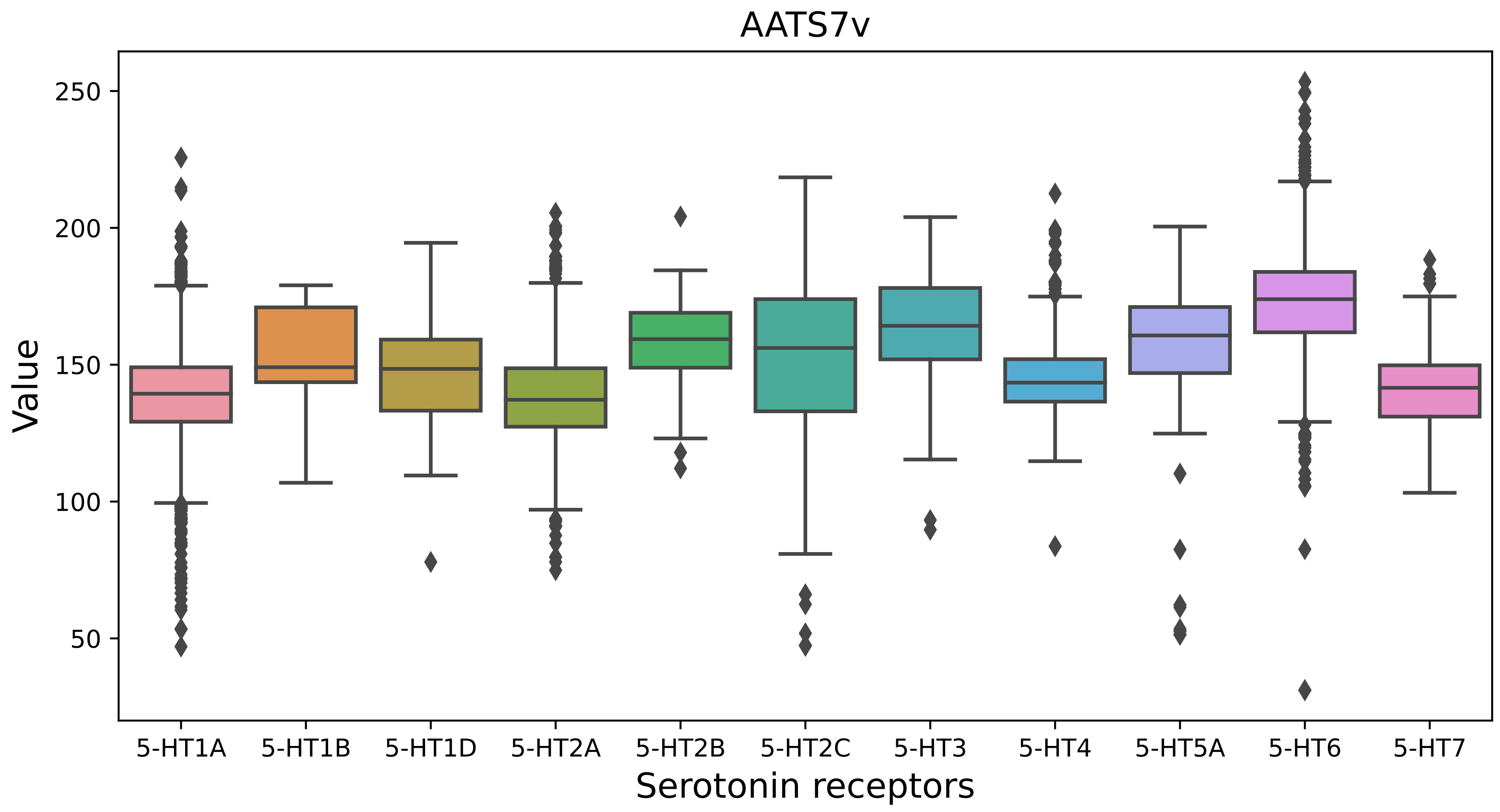

AATS7v
Value
250
200
150
100
50
5-HT1A
5-HT1B
5-HT1D
5-HT2A
5-HT2B
5-HT2C
5-HT3
5-HT4
5-HT5A
5-HT6
5-HT7
Serotonin receptors

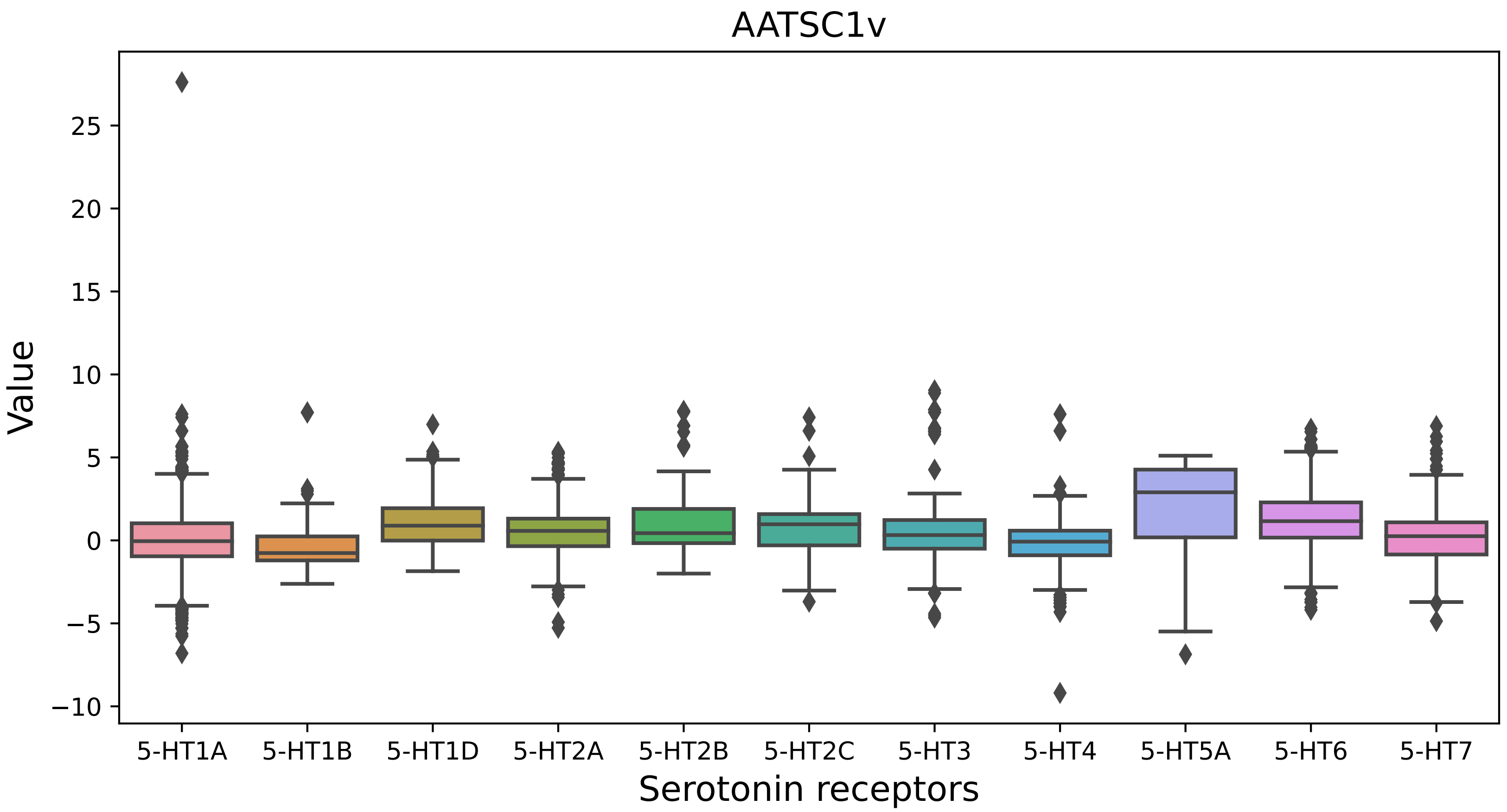
AATSC1v
Value
25
20
15
10
5
0
−5
−10
5-HT1A
5-HT1B
5-HT1D
5-HT2A
5-HT2B
5-HT2C
5-HT3
5-HT4
5-HT5A
5-HT6
5-HT7
Serotonin receptors

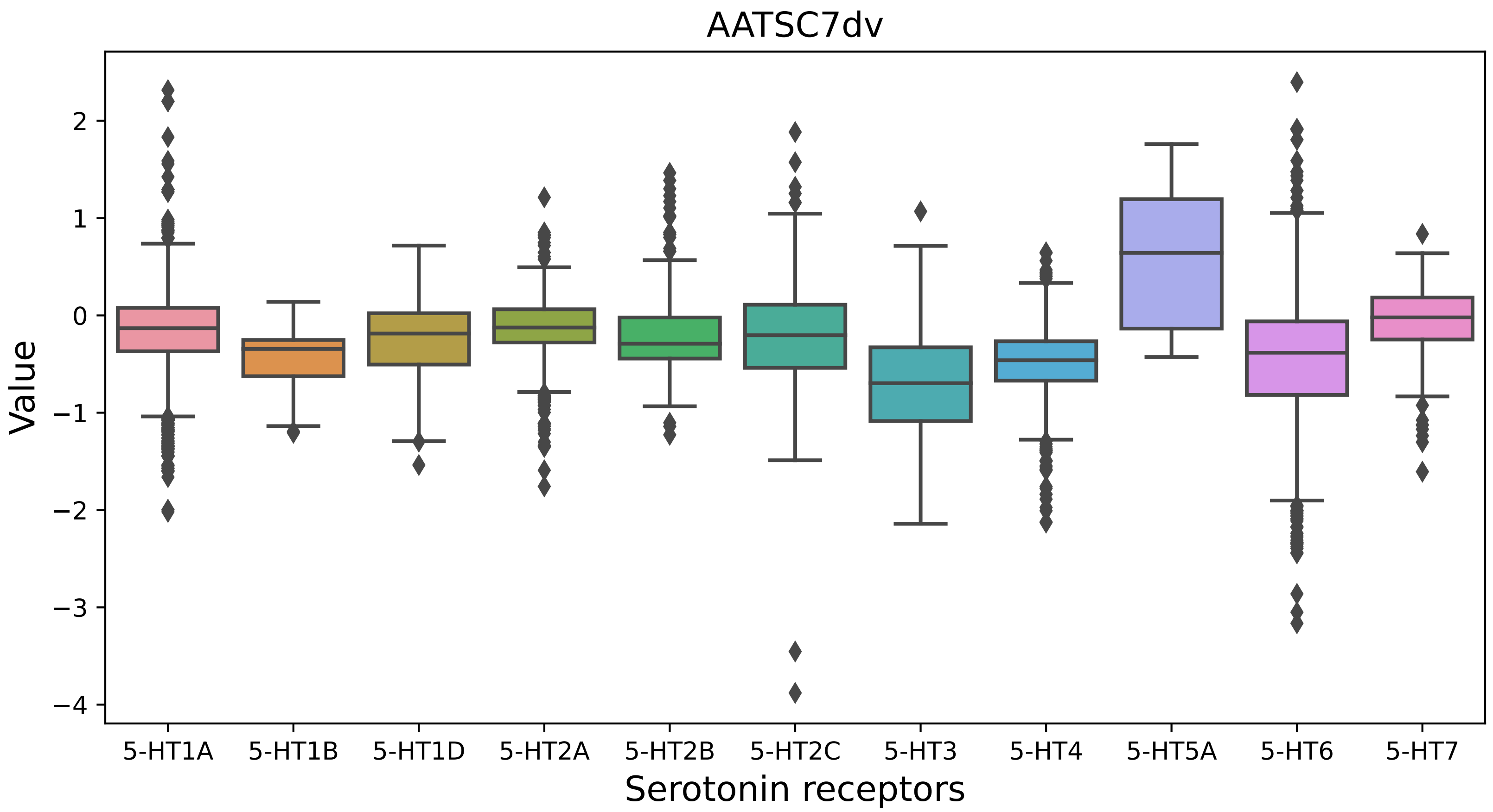

AATSC7dv
Value
2
1
0
−1
−2
−3
−4
5-HT1A
5-HT1B
5-HT1D
5-HT2A
5-HT2B
5-HT2C
5-HT3
5-HT4
5-HT5A
5-HT6
5-HT7
Serotonin receptors

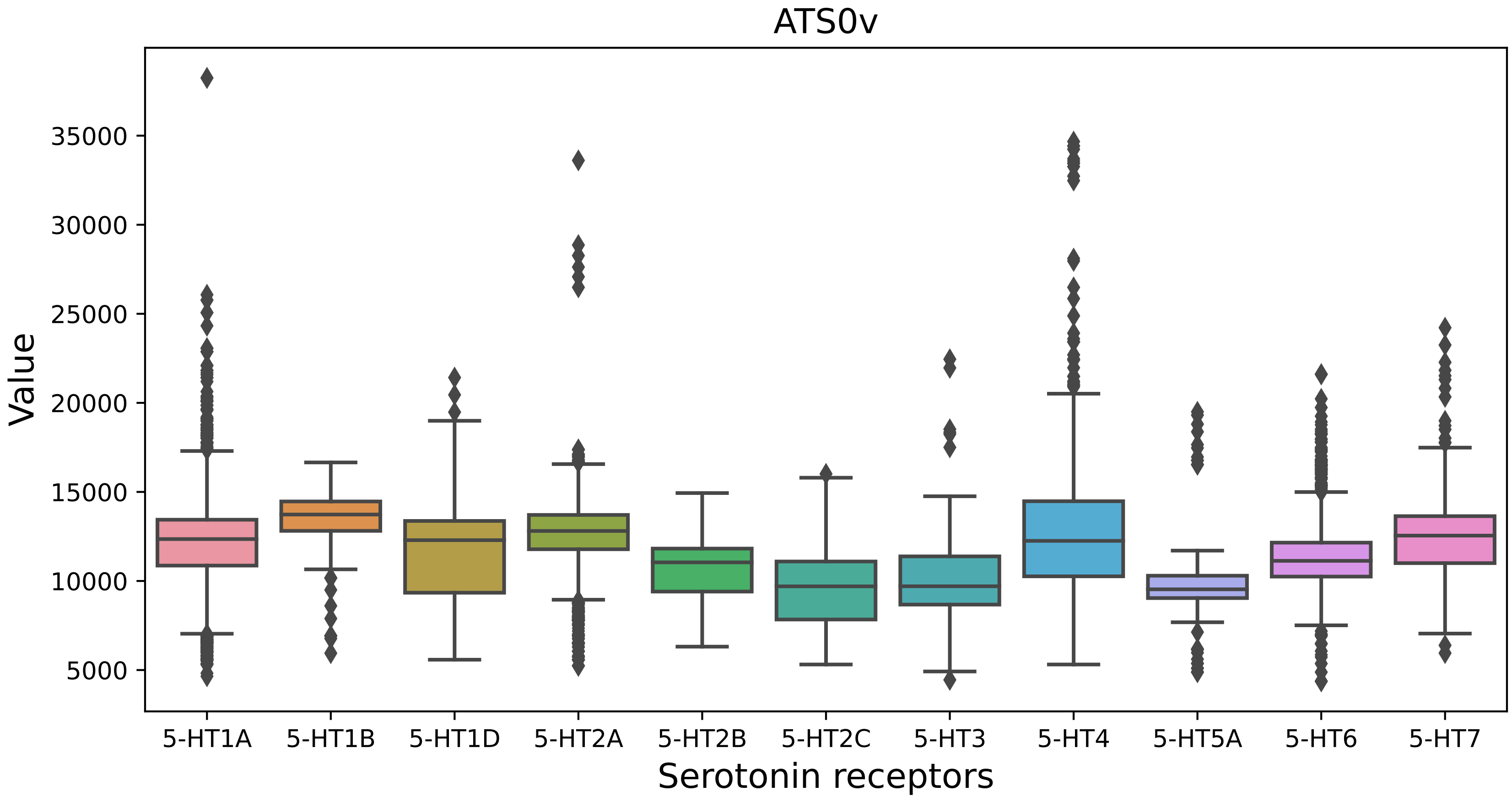
ATS0v
Value
35000
30000
25000
20000
15000
10000
5000
5-HT1A
5-HT1B
5-HT1D
5-HT2A
5-HT2B
5-HT2C
5-HT3
5-HT4
5-HT5A
5-HT6
5-HT7
Serotonin receptors

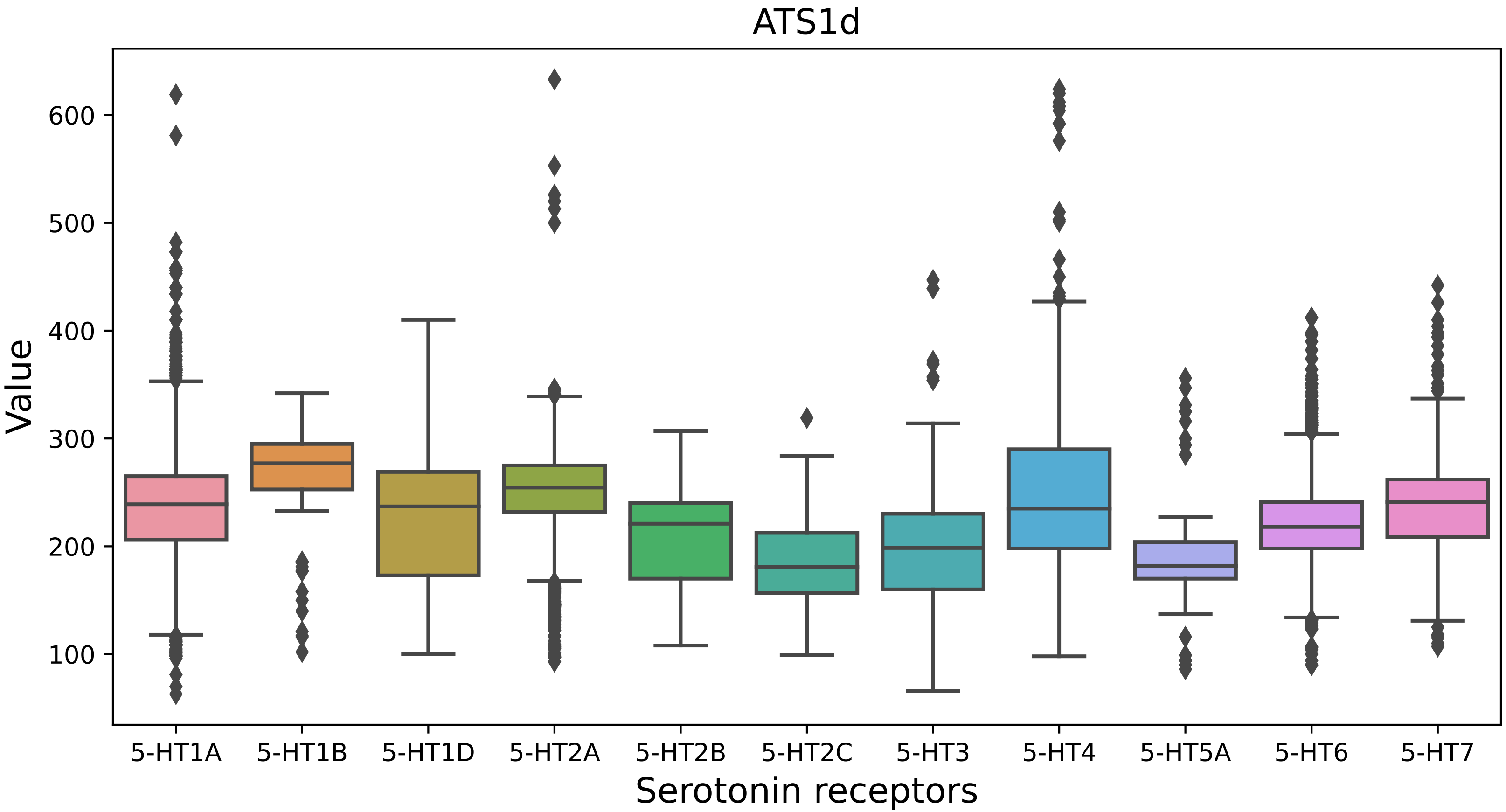

ATS1d
Value
600
500
400
300
200
100
5-HT1A
5-HT1B
5-HT1D
5-HT2A
5-HT2B
5-HT2C
5-HT3
5-HT4
5-HT5A
5-HT6
5-HT7
Serotonin receptors

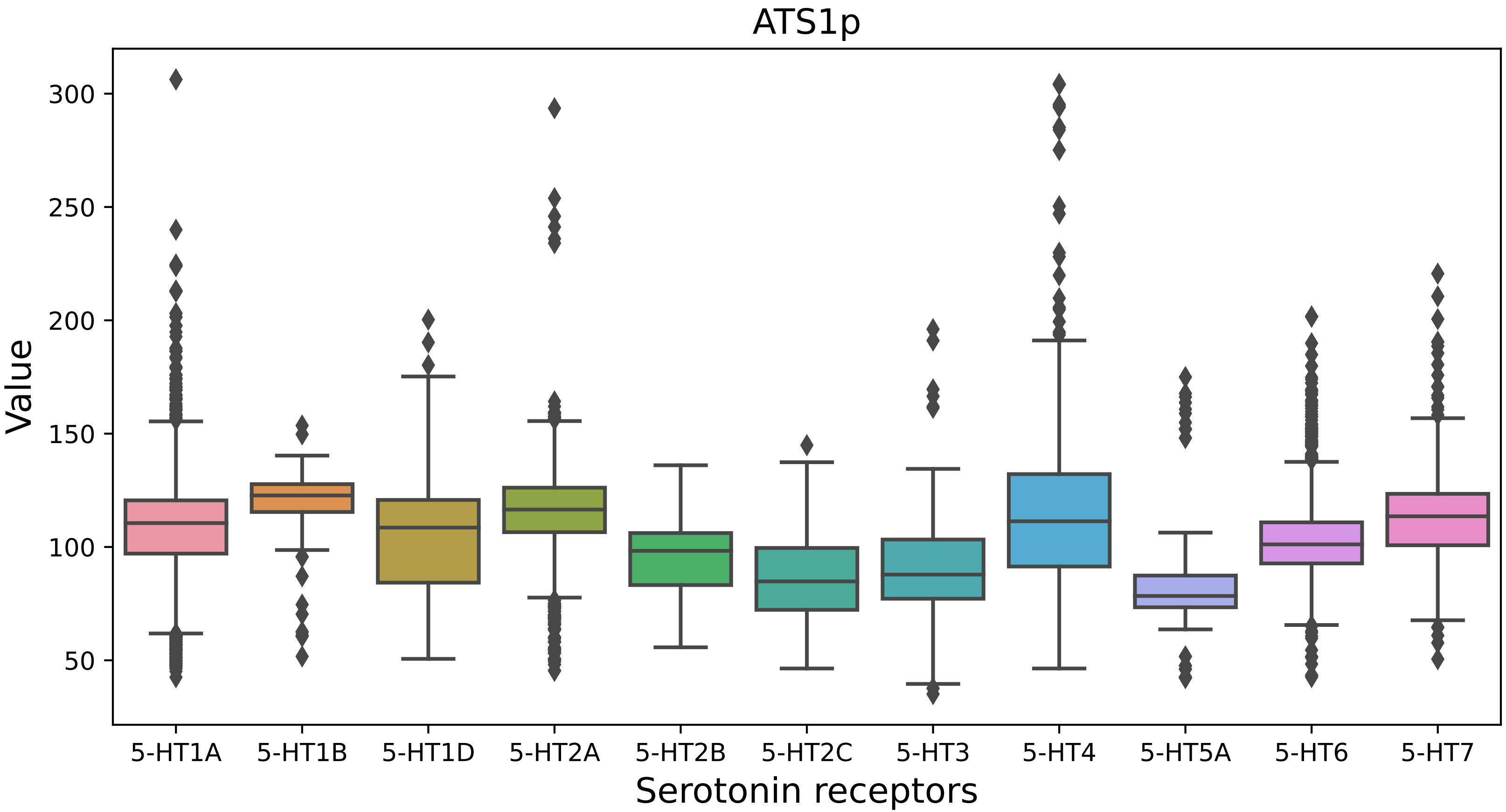
ATS1p
Value
300
250
200
150
100
50
5-HT1A
5-HT1B
5-HT1D
5-HT2A
5-HT2B
5-HT2C
5-HT3
5-HT4
5-HT5A
5-HT6
5-HT7
Serotonin receptors

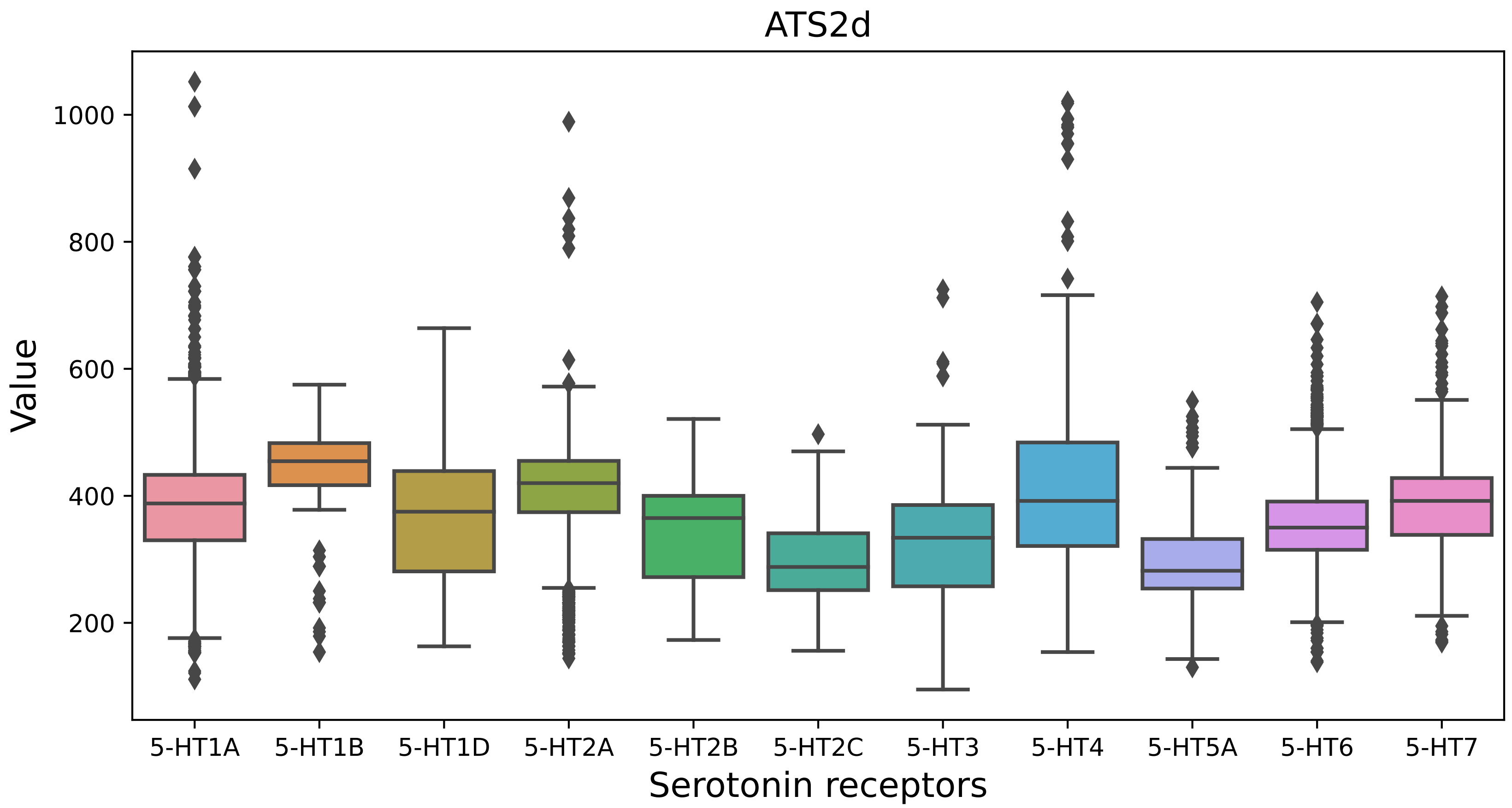

ATS2d
Value
1000
800
600
400
200
5-HT1A
5-HT1B
5-HT1D
5-HT2A
5-HT2B
5-HT2C
5-HT3
5-HT4
5-HT5A
5-HT6
5-HT7
Serotonin receptors

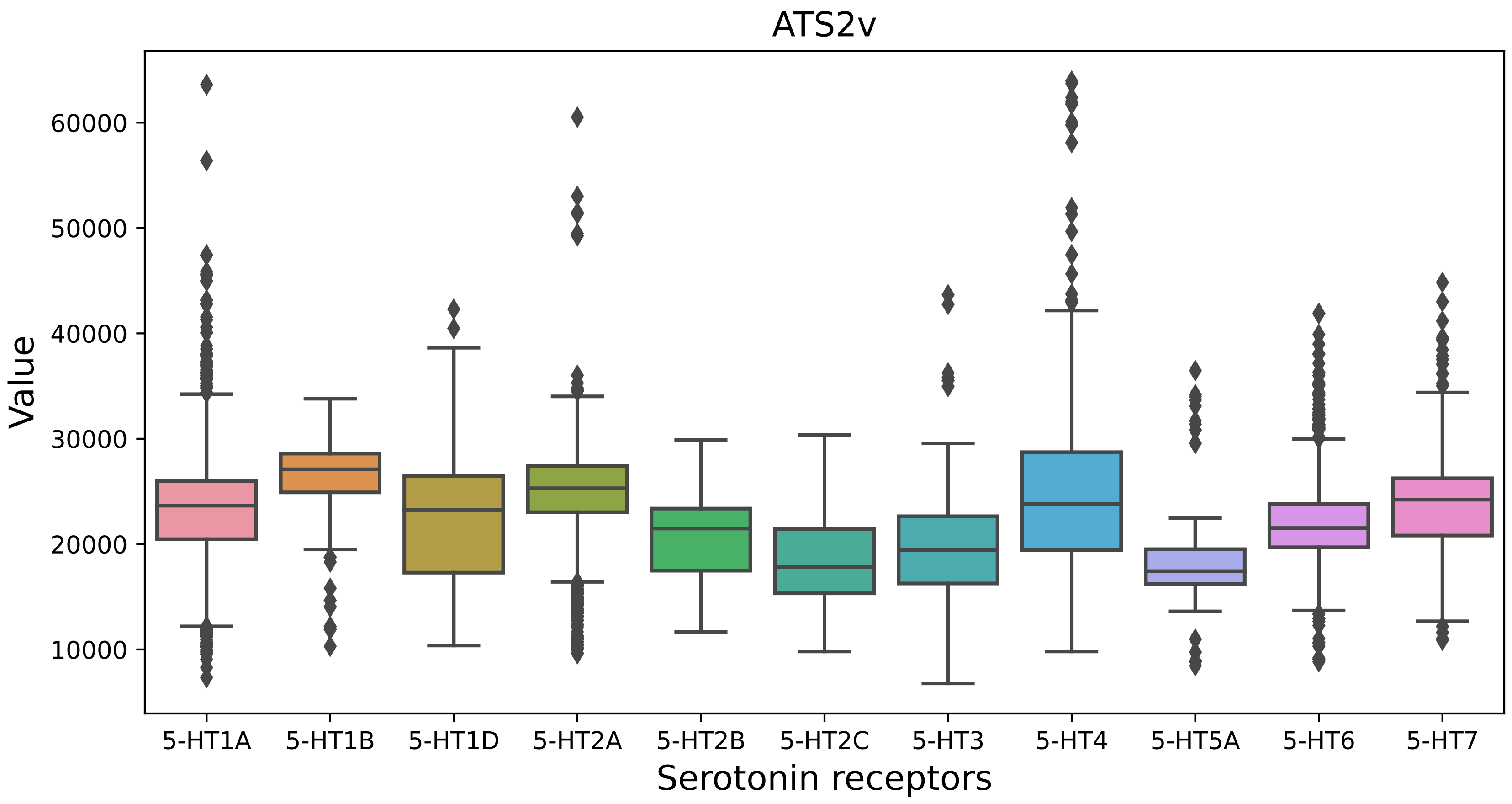
ATS2v
Value
60000
50000
40000
30000
20000
10000
5-HT1A
5-HT1B
5-HT1D
5-HT2A
5-HT2B
5-HT2C
5-HT3
5-HT4
5-HT5A
5-HT6
5-HT7
Serotonin receptors

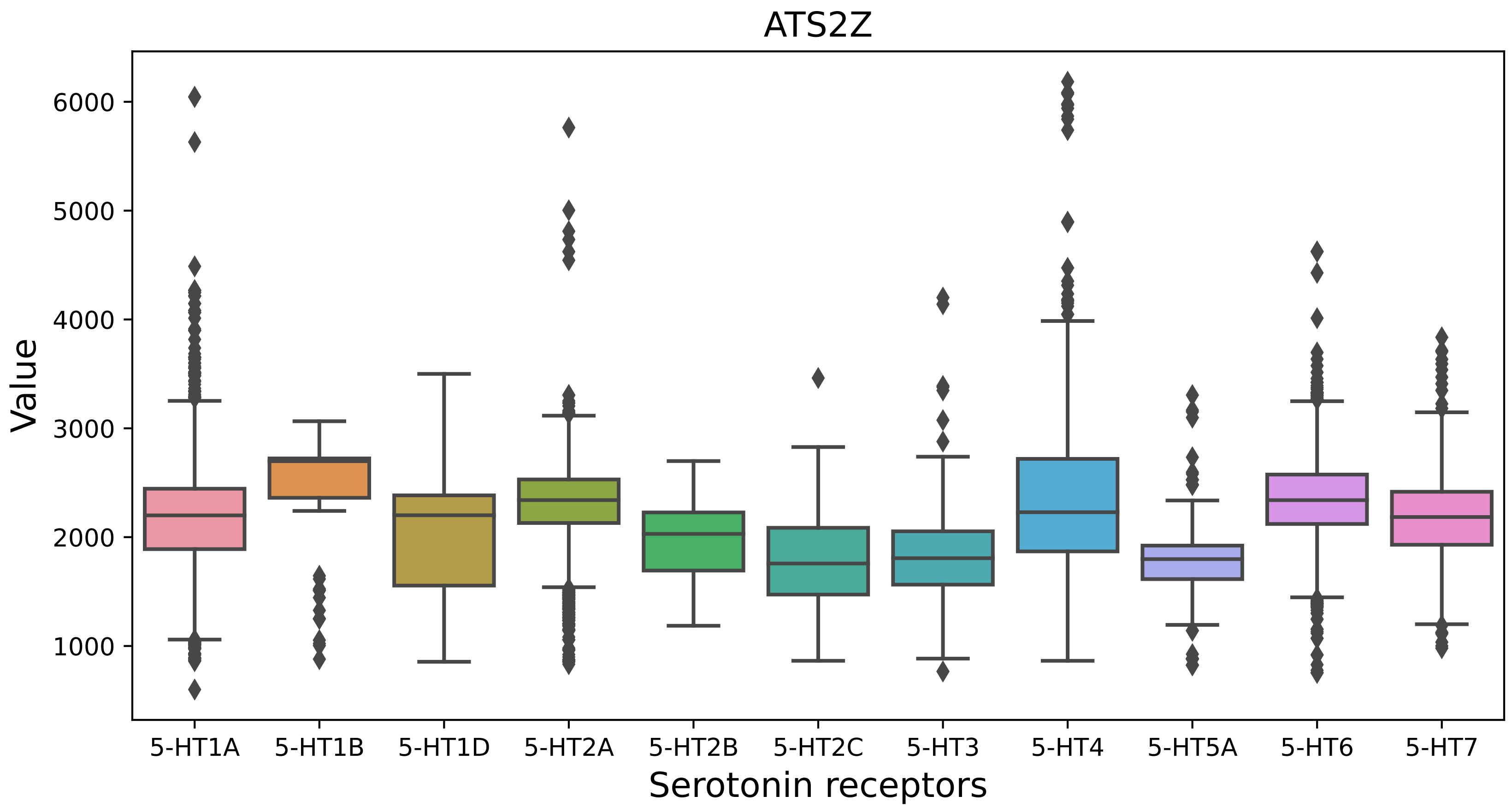
ATS2Z
Value
6000
5000
4000
3000
2000
1000
5-HT1A
5-HT1B
5-HT1D
5-HT2A
5-HT2B
5-HT2C
5-HT3
5-HT4
5-HT5A
5-HT6
5-HT7
Serotonin receptors

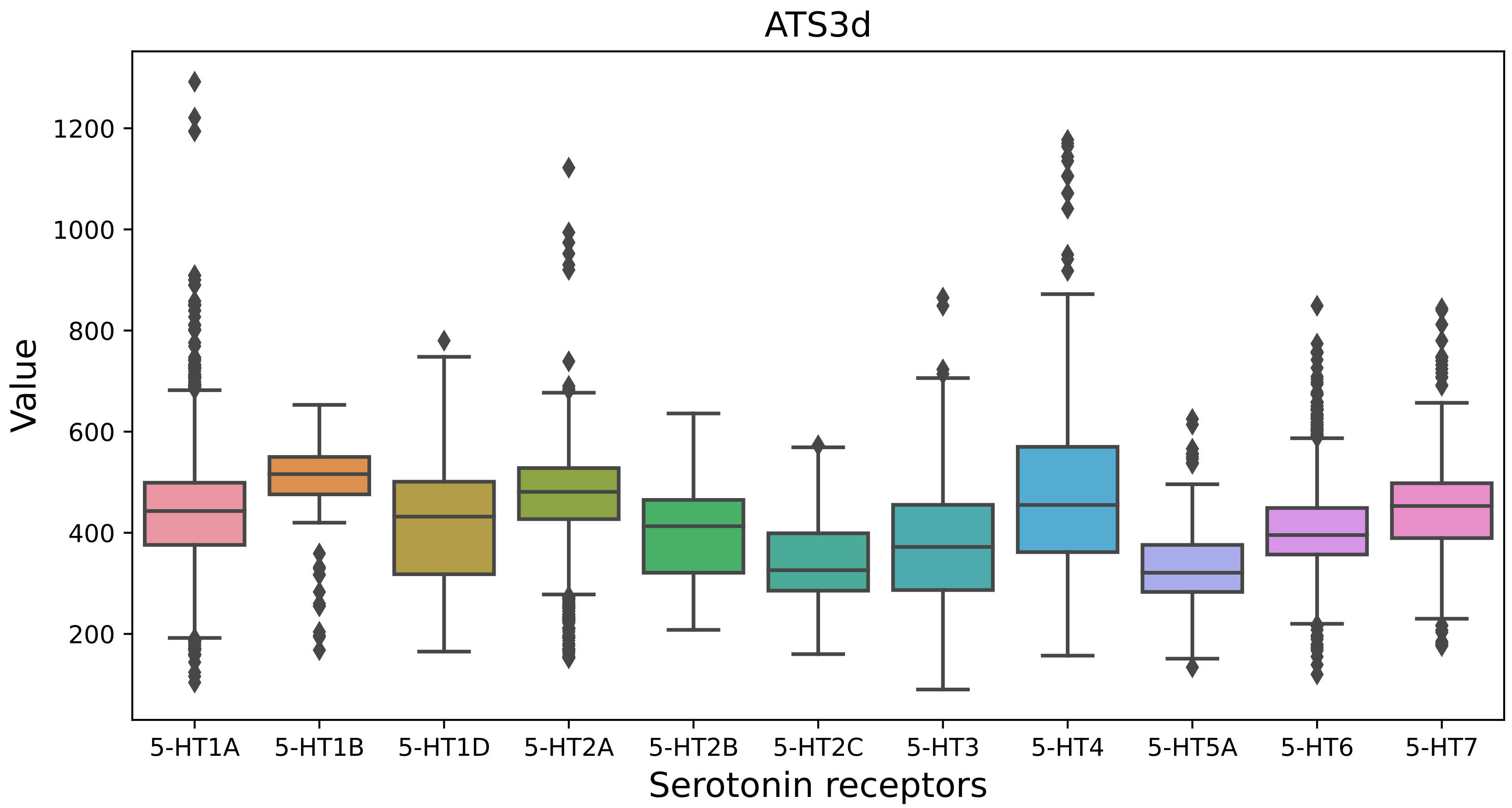

ATS3d
Value
1200
1000
800
600
400
200
5-HT1A
5-HT1B
5-HT1D
5-HT2A
5-HT2B
5-HT2C
5-HT3
5-HT4
5-HT5A
5-HT6
5-HT7
Serotonin receptors

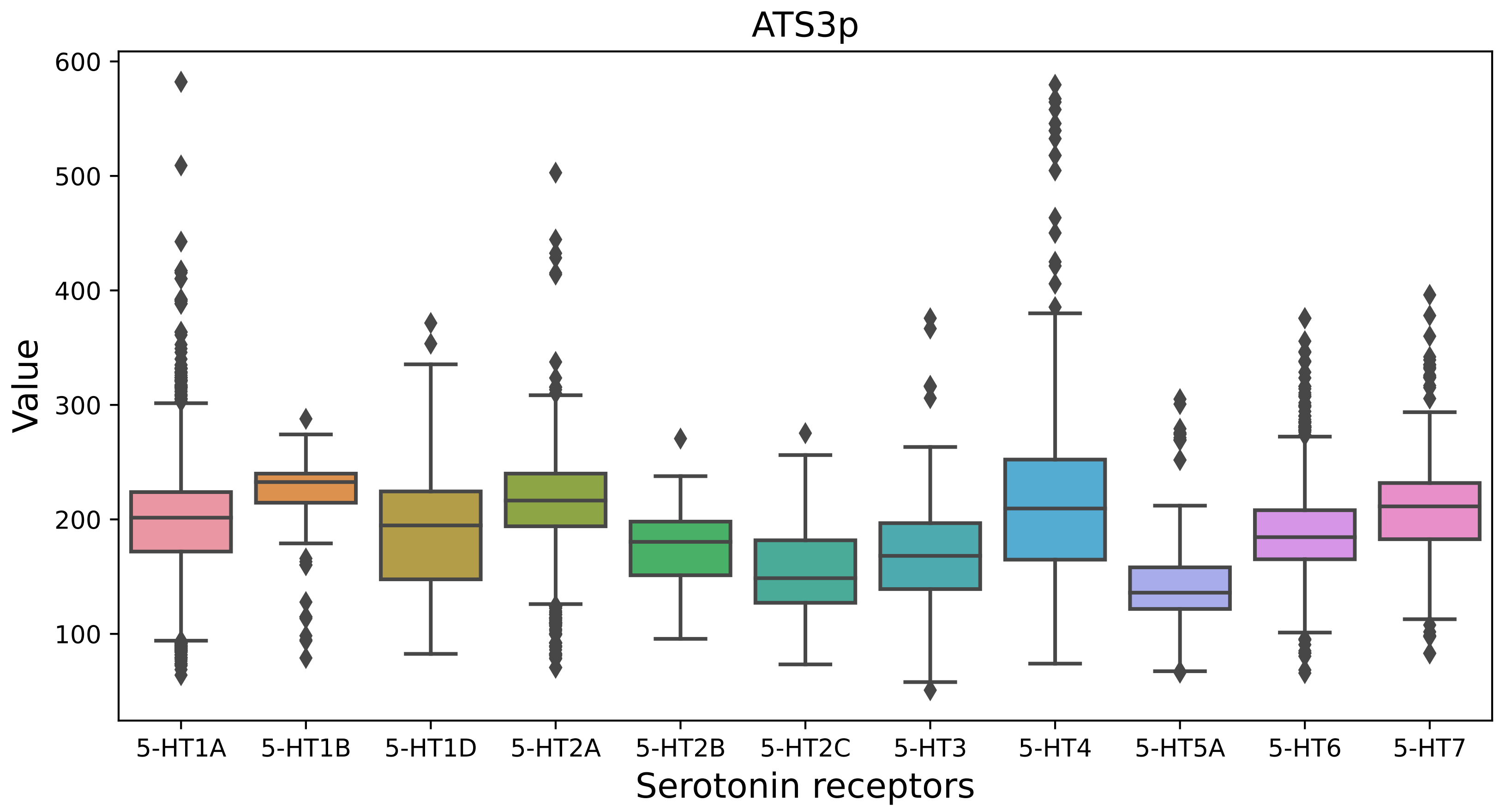
ATS3p
Value
600
500
400
300
200
100
5-HT1A
5-HT1B
5-HT1D
5-HT2A
5-HT2B
5-HT2C
5-HT3
5-HT4
5-HT5A
5-HT6
5-HT7
Serotonin receptors

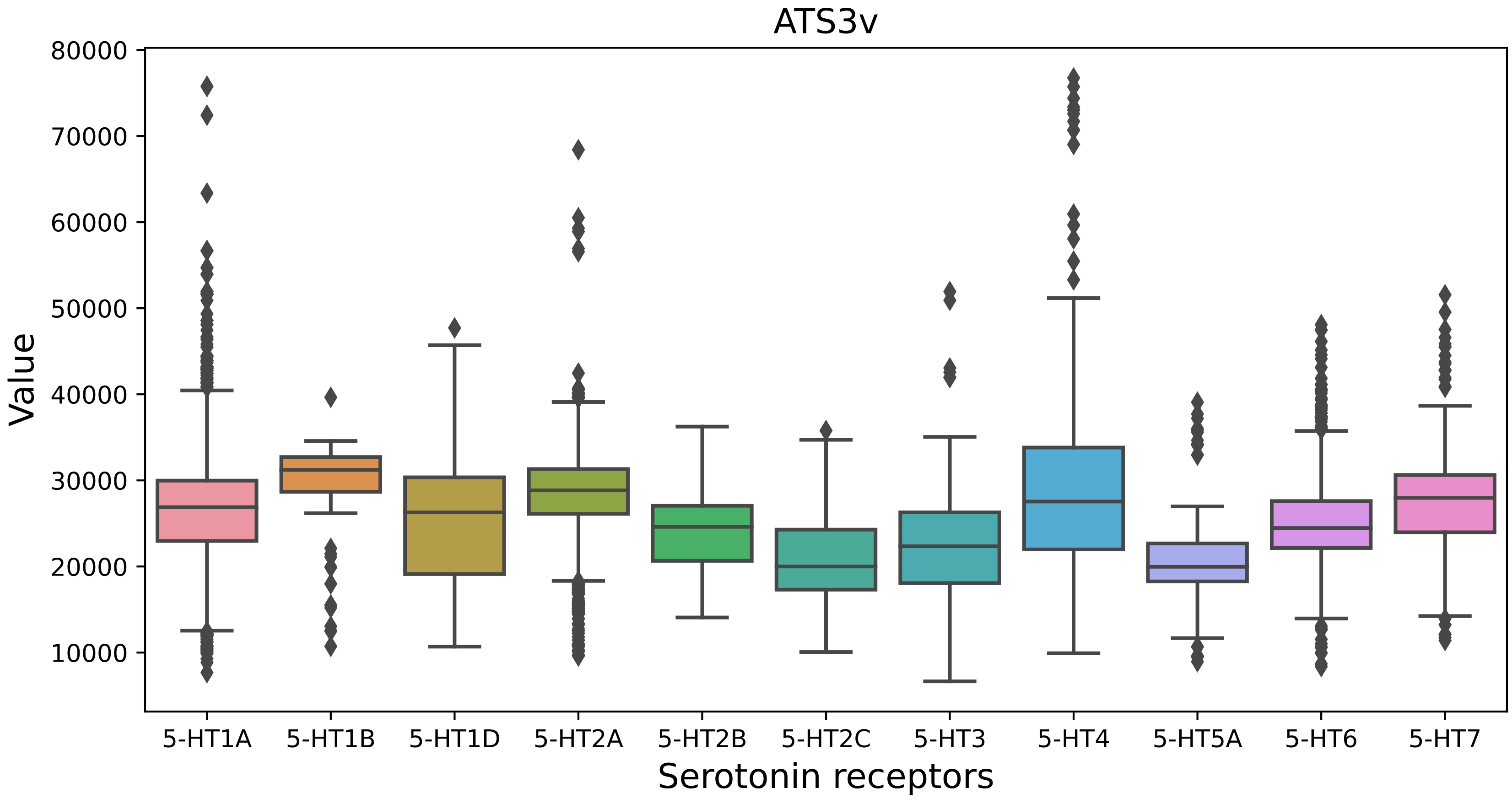
ATS3v
80000
70000
60000
50000
40000
30000
20000
10000
Value
5-HT1A
5-HT1B
5-HT1D
5-HT2A
5-HT2B
5-HT2C
5-HT3
5-HT4
5-HT5A
5-HT6
5-HT7
Serotonin receptors

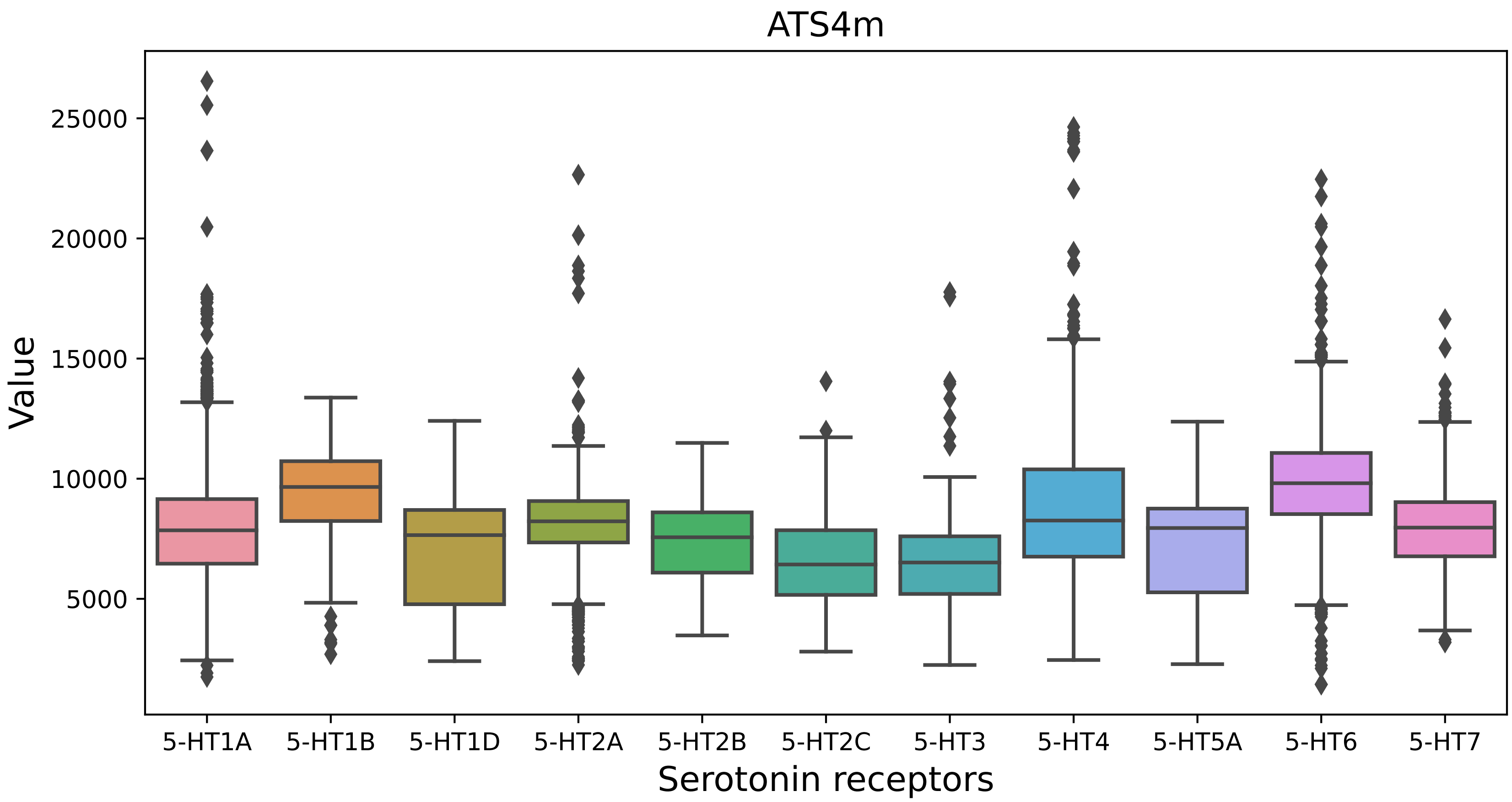

ATS4m
Value
25000
20000
15000
10000
5000
5-HT1A
5-HT1B
5-HT1D
5-HT2A
5-HT2B
5-HT2C
5-HT3
5-HT4
5-HT5A
5-HT6
5-HT7
Serotonin receptors

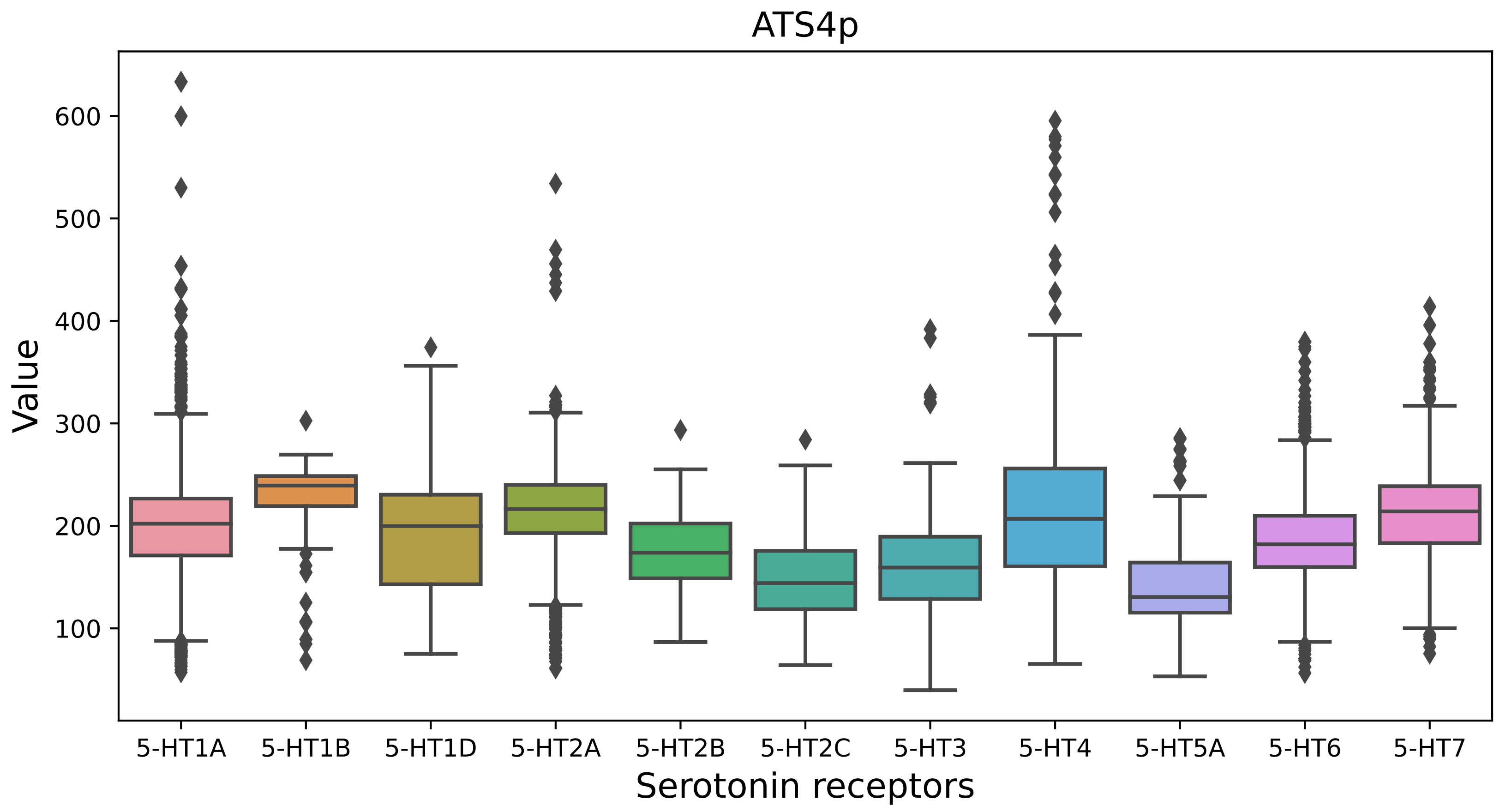

ATS4p
Value
600
500
400
300
200
100
5-HT1A
5-HT1B
5-HT1D
5-HT2A
5-HT2B
5-HT2C
5-HT3
5-HT4
5-HT5A
5-HT6
5-HT7
Serotonin receptors

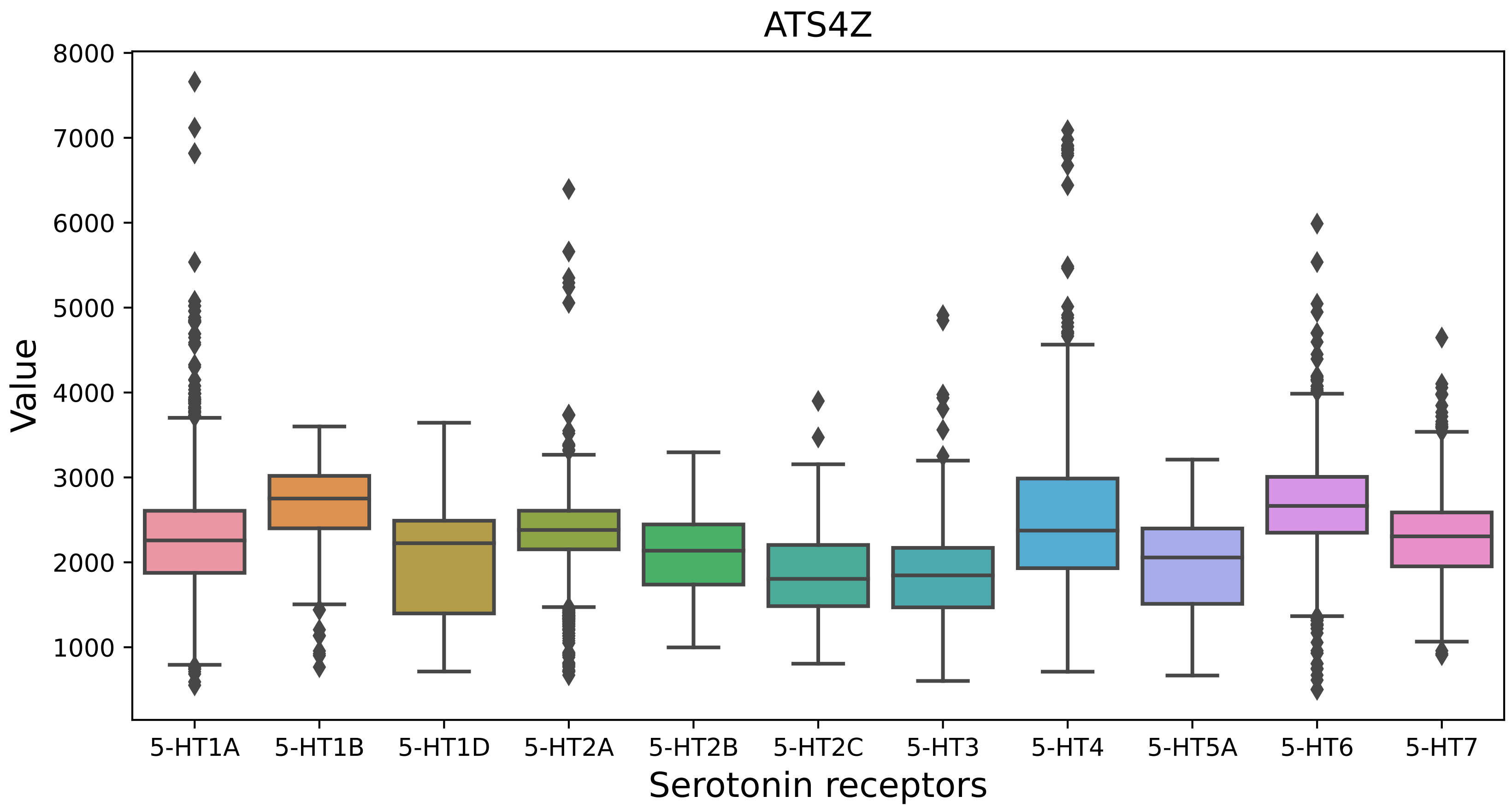
ATS4Z
Value
8000
7000
6000
5000
4000
3000
2000
1000
5-HT1A
5-HT1B
5-HT1D
5-HT2A
5-HT2B
5-HT2C
5-HT3
5-HT4
5-HT5A
5-HT6
5-HT7
Serotonin receptors

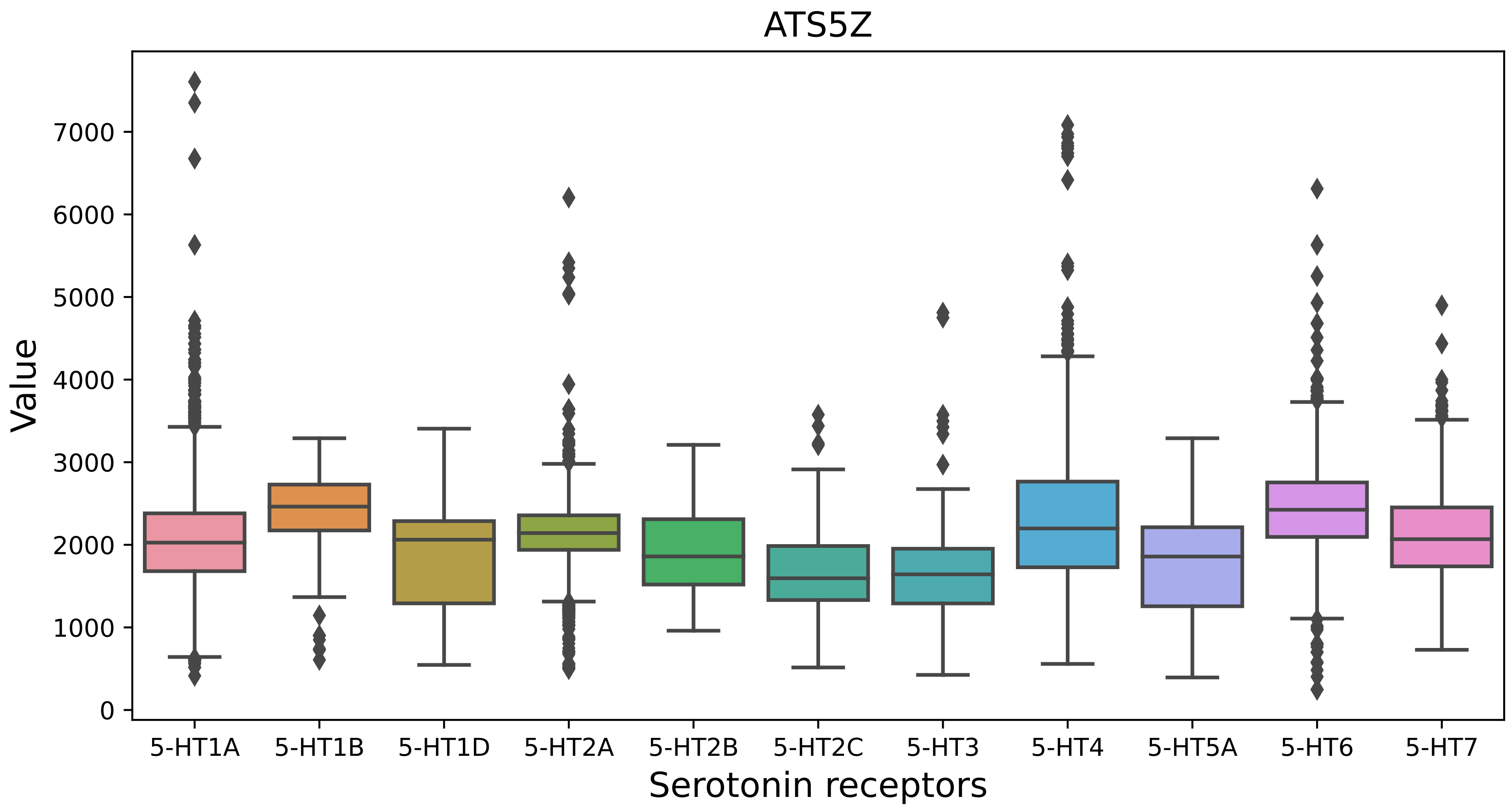
ATS5Z
Value
7000
6000
5000
4000
3000
2000
1000
0
5-HT1A
5-HT1B
5-HT1D
5-HT2A
5-HT2B
5-HT2C
5-HT3
5-HT4
5-HT5A
5-HT6
5-HT7
Serotonin receptors

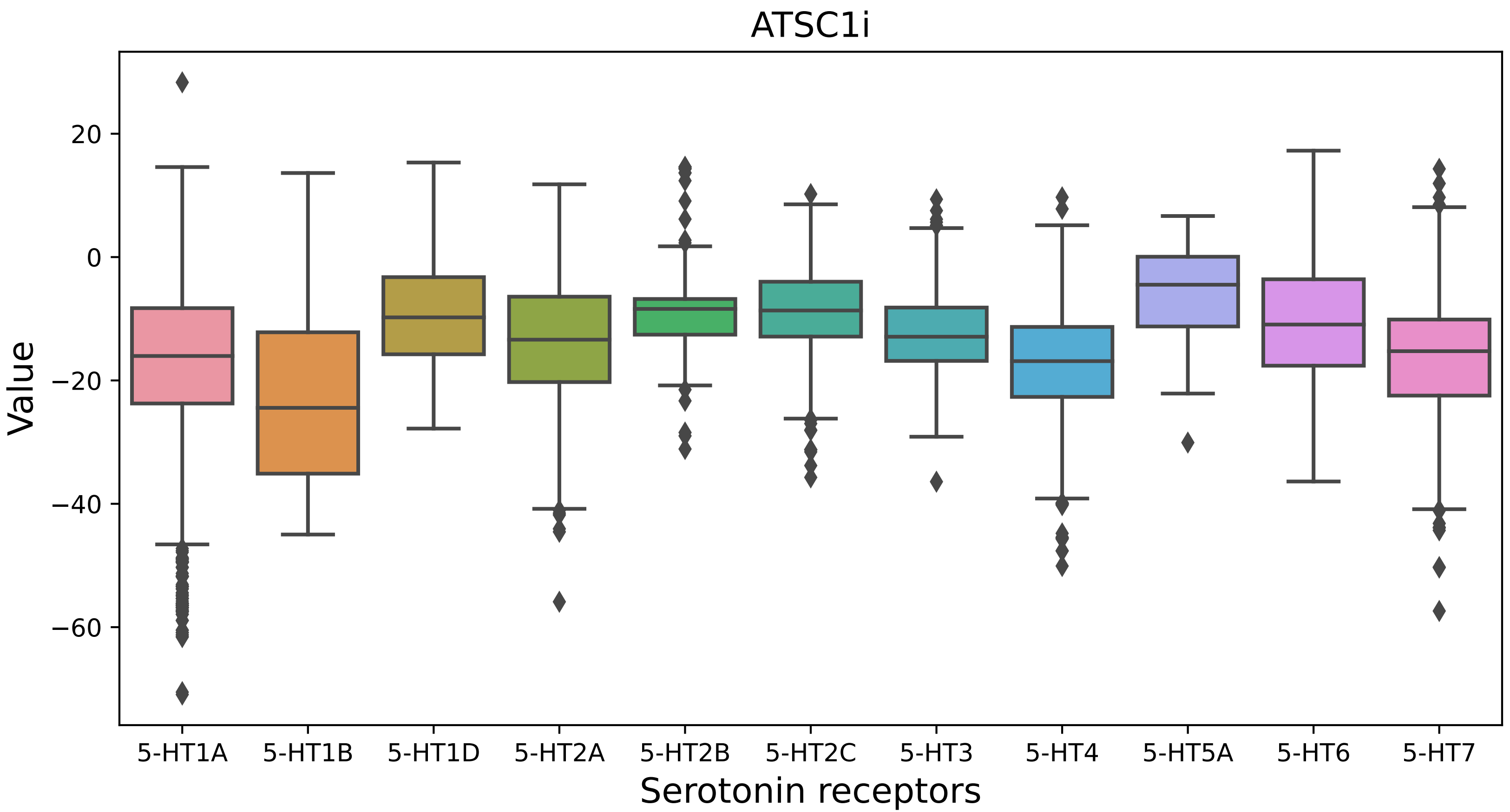
ATSC1i
Value
20
0
−20
−40
−60
5-HT1A
5-HT1B
5-HT1D
5-HT2A
5-HT2B
5-HT2C
5-HT3
5-HT4
5-HT5A
5-HT6
5-HT7
Serotonin receptors

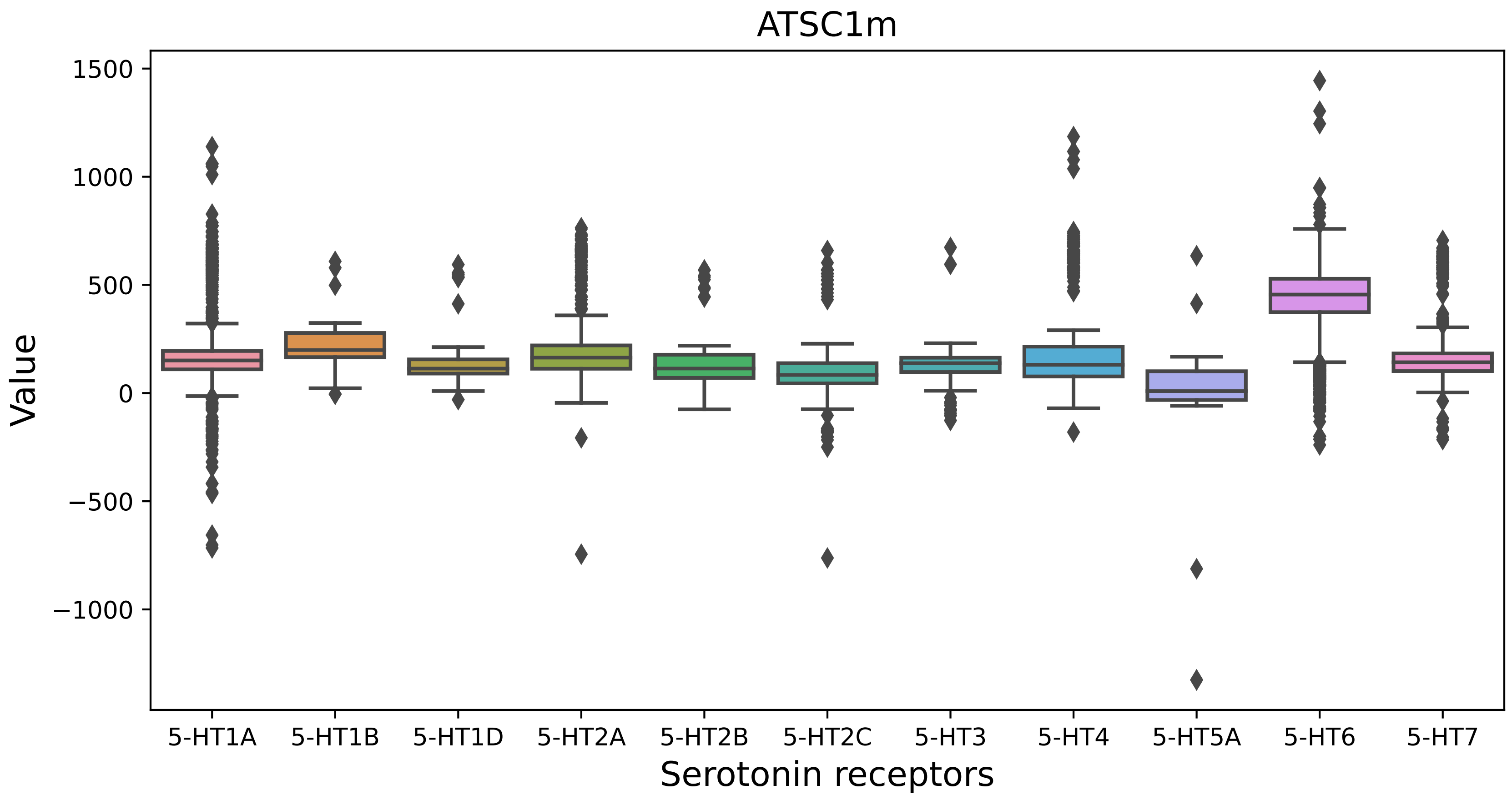

ATSC1m
Value
1500
1000
500
0
−500
−1000
5-HT1A
5-HT1B
5-HT1D
5-HT2A
5-HT2B
5-HT2C
5-HT3
5-HT4
5-HT5A
5-HT6
5-HT7
Serotonin receptors

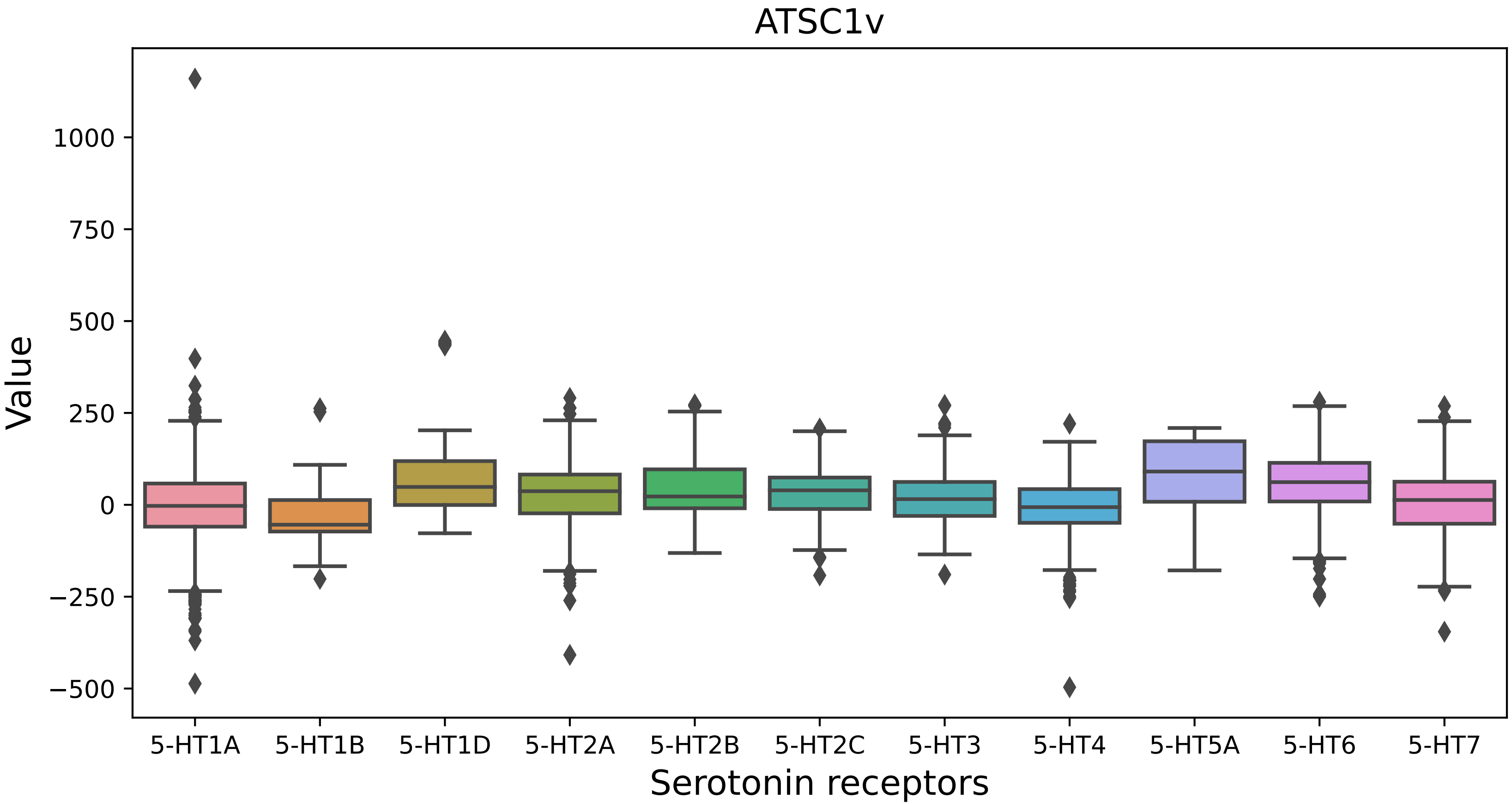
ATSC1v
Value
1000
750
500
250
0
−250
−500
5-HT1A
5-HT1B
5-HT1D
5-HT2A
5-HT2B
5-HT2C
5-HT3
5-HT4
5-HT5A
5-HT6
5-HT7
Serotonin receptors

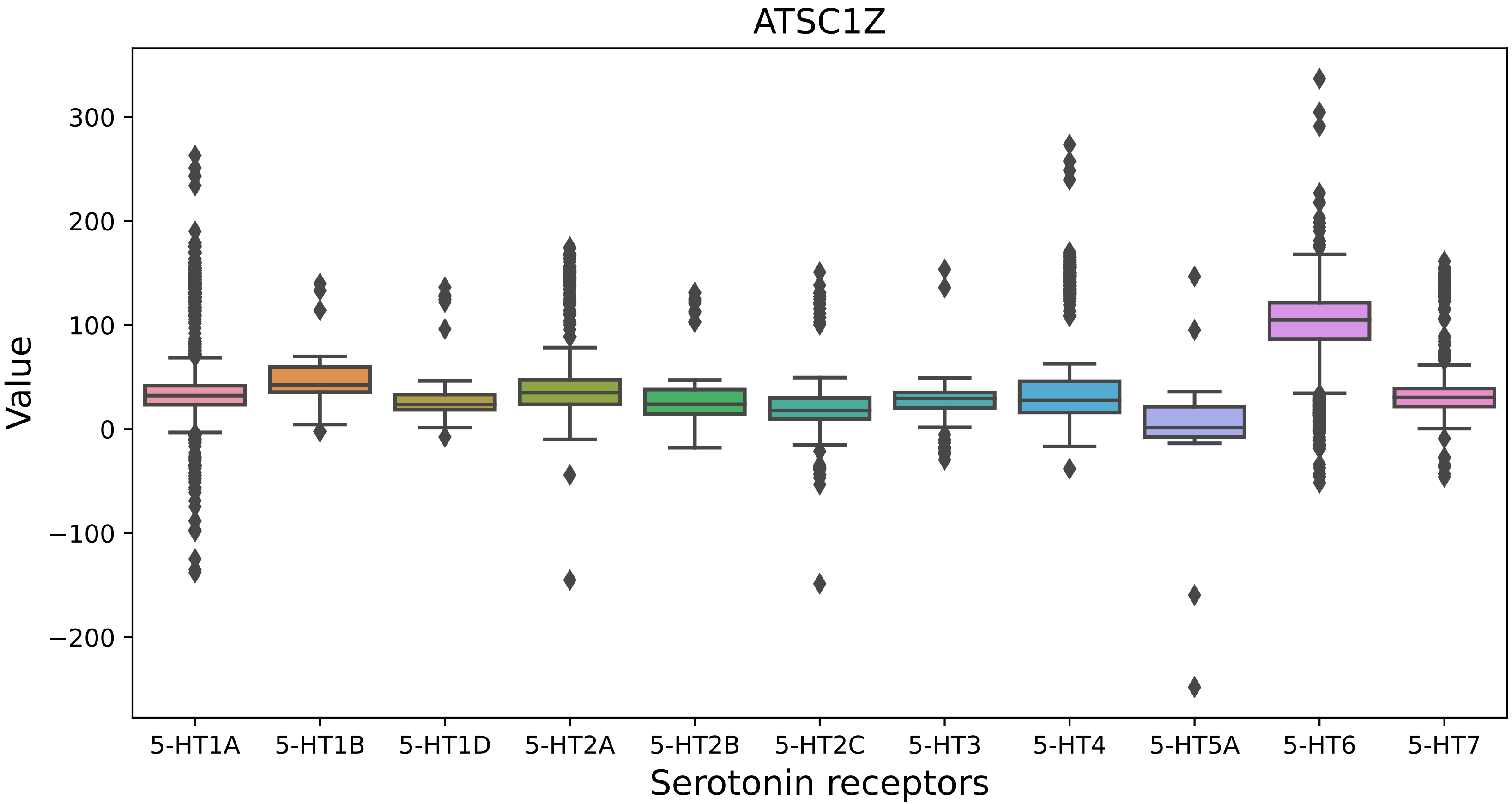

ATSC1Z
300
200
100
0
−100
−200
Value
5-HT1A
5-HT1B
5-HT1D
5-HT2A
5-HT2B
5-HT2C
5-HT3
5-HT4
5-HT5A
5-HT6
5-HT7
Serotonin receptors

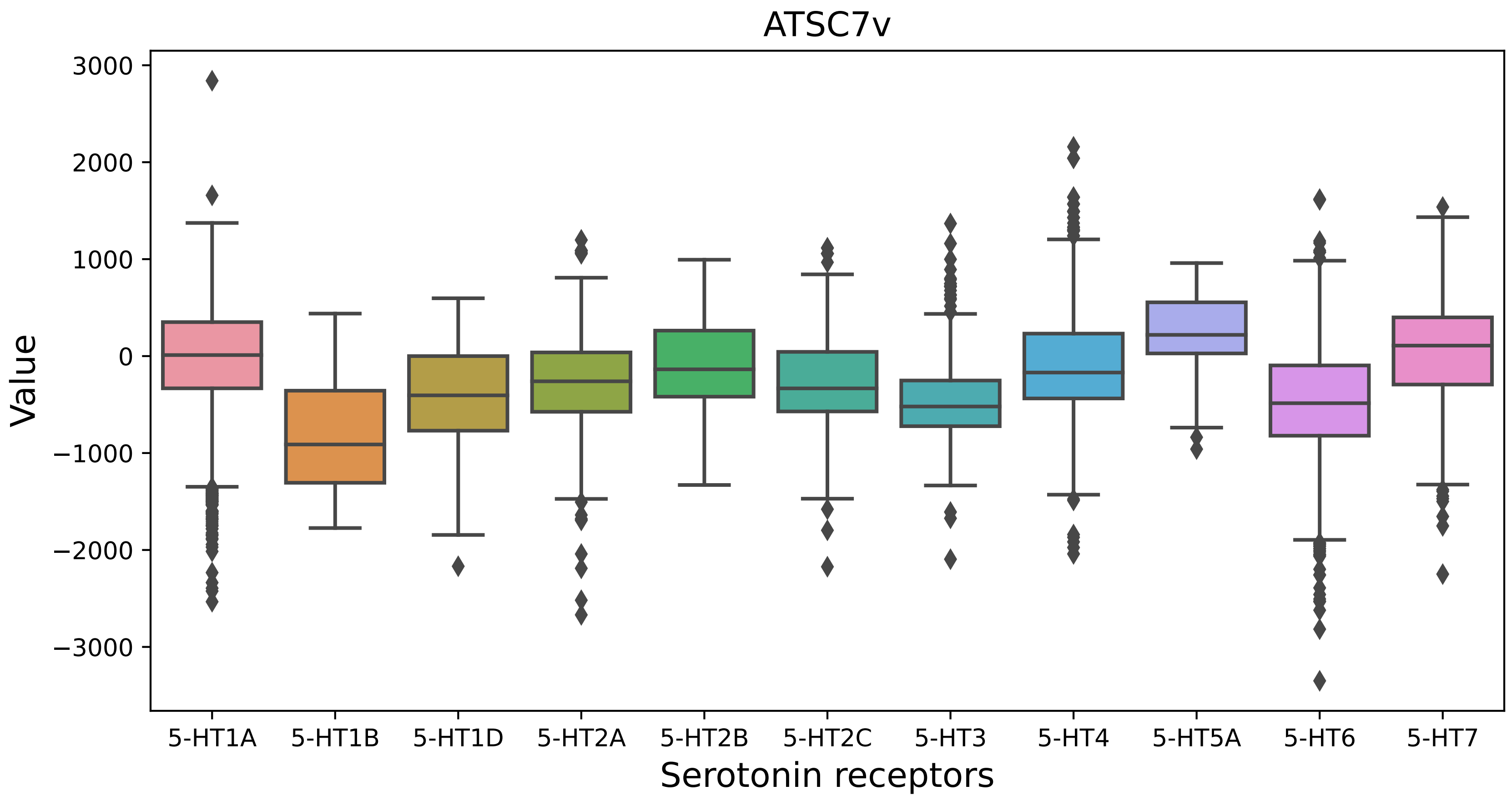

ATSC7v
Value
3000
2000
1000
0
−1000
−2000
−3000
5-HT1A
5-HT1B
5-HT1D
5-HT2A
5-HT2B
5-HT2C
5-HT3
5-HT4
5-HT5A
5-HT6
5-HT7
Serotonin receptors

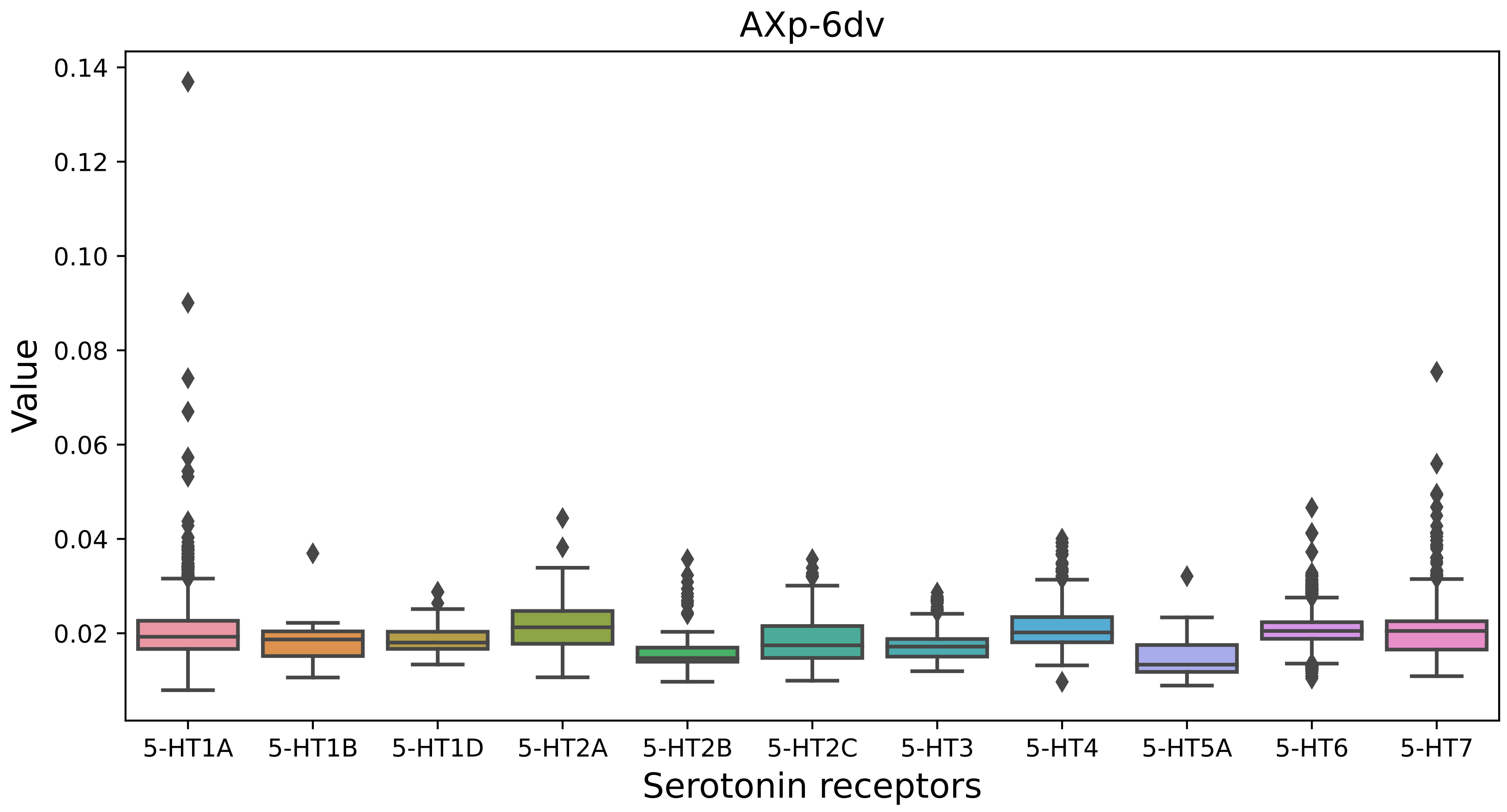
AXp-6dv
Value
0.14
0.12
0.10
0.08
0.06
0.04
0.02
5-HT1A
5-HT1B
5-HT1D
5-HT2A
5-HT2B
5-HT2C
5-HT3
5-HT4
5-HT5A
5-HT6
5-HT7
Serotonin receptors

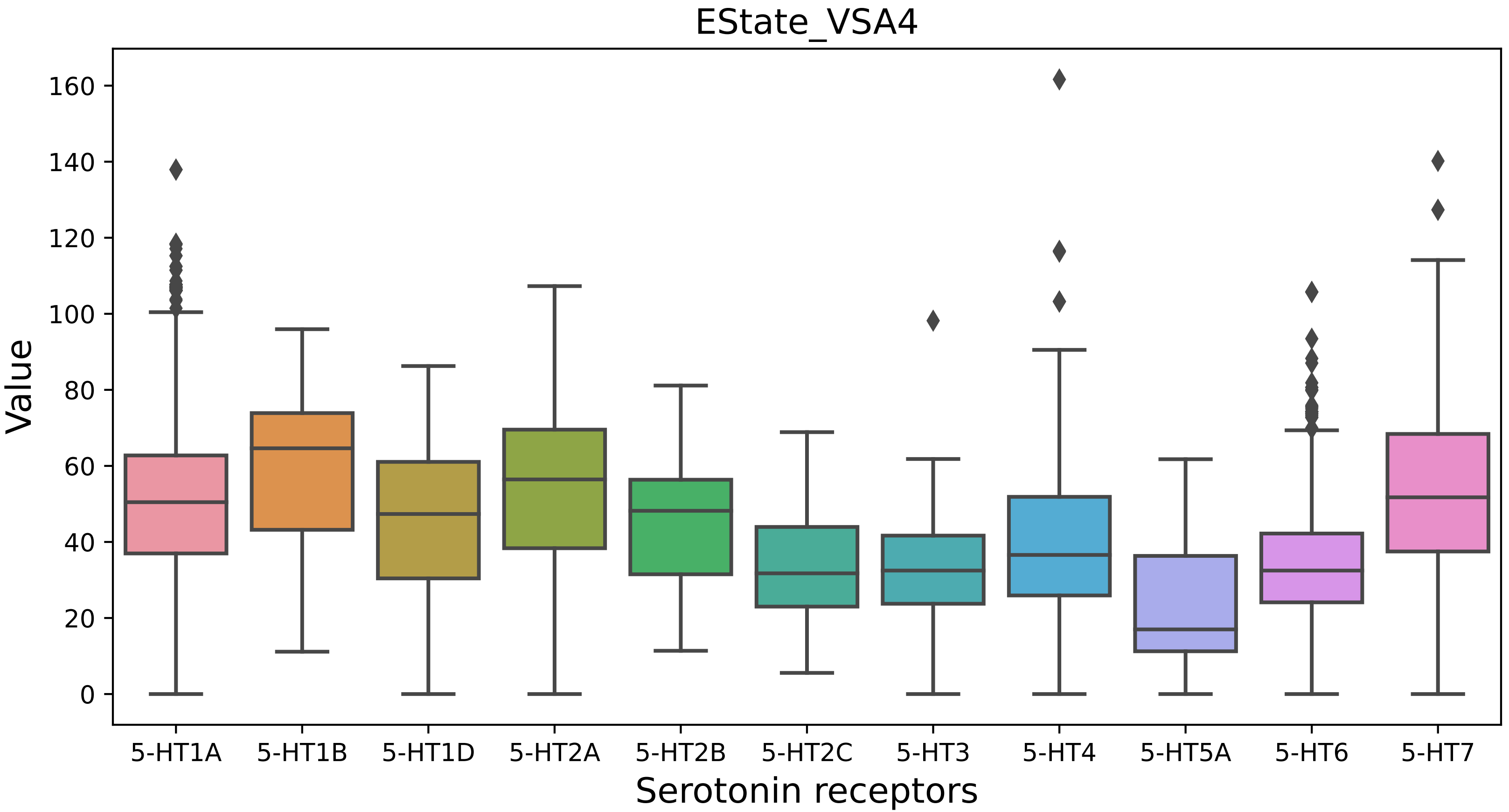
EState_VSA4
Value
Serotonin receptors
0
20
40
60
80
100
120
140
160
5-HT1A
5-HT1B
5-HT1D
5-HT2A
5-HT2B
5-HT2C
5-HT3
5-HT4
5-HT5A
5-HT6
5-HT7

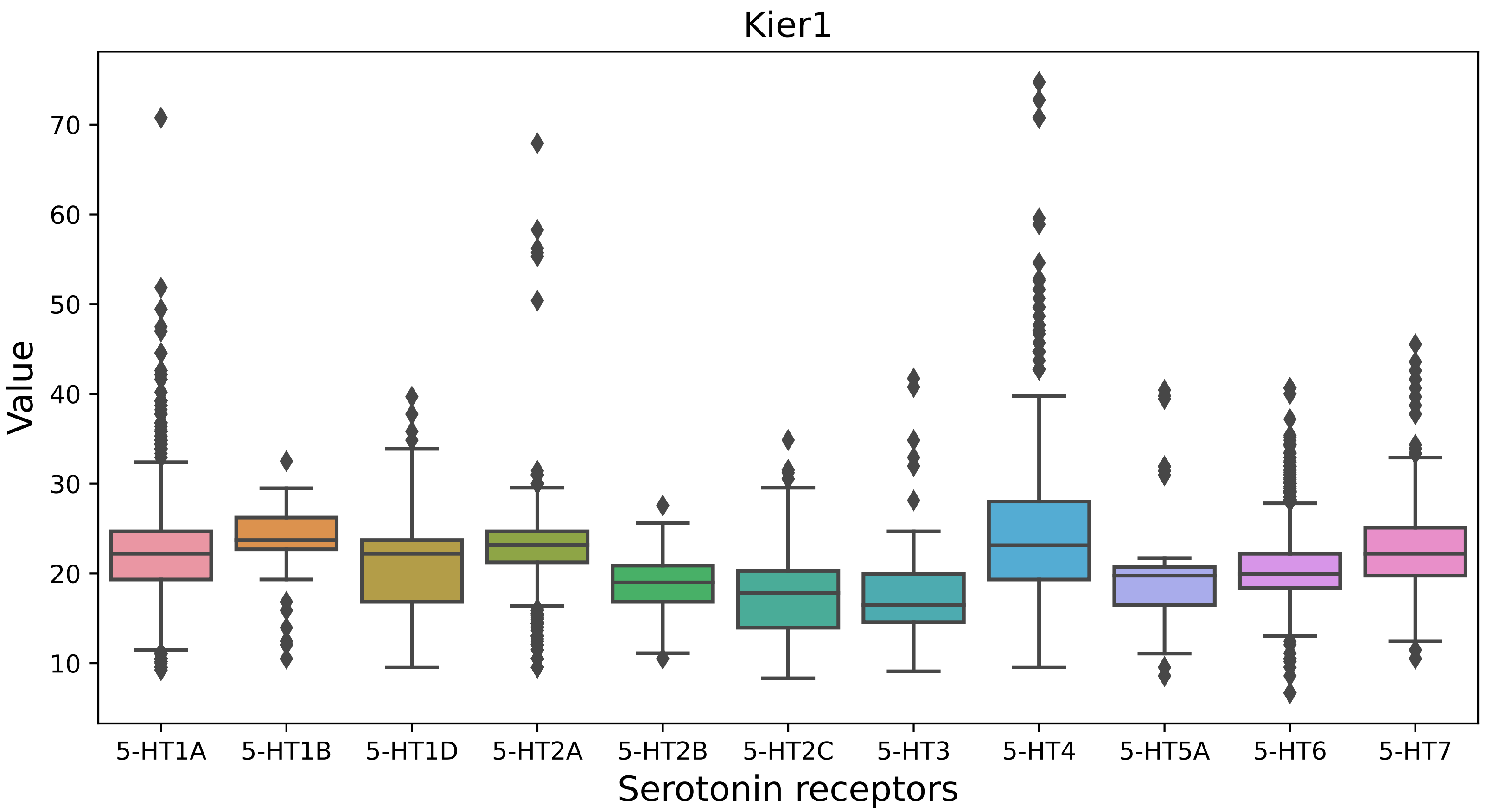

Kier1
Value
70
60
50
40
30
20
10
5-HT1A
5-HT1B
5-HT1D
5-HT2A
5-HT2B
5-HT2C
5-HT3
5-HT4
5-HT5A
5-HT6
5-HT7
Serotonin receptors

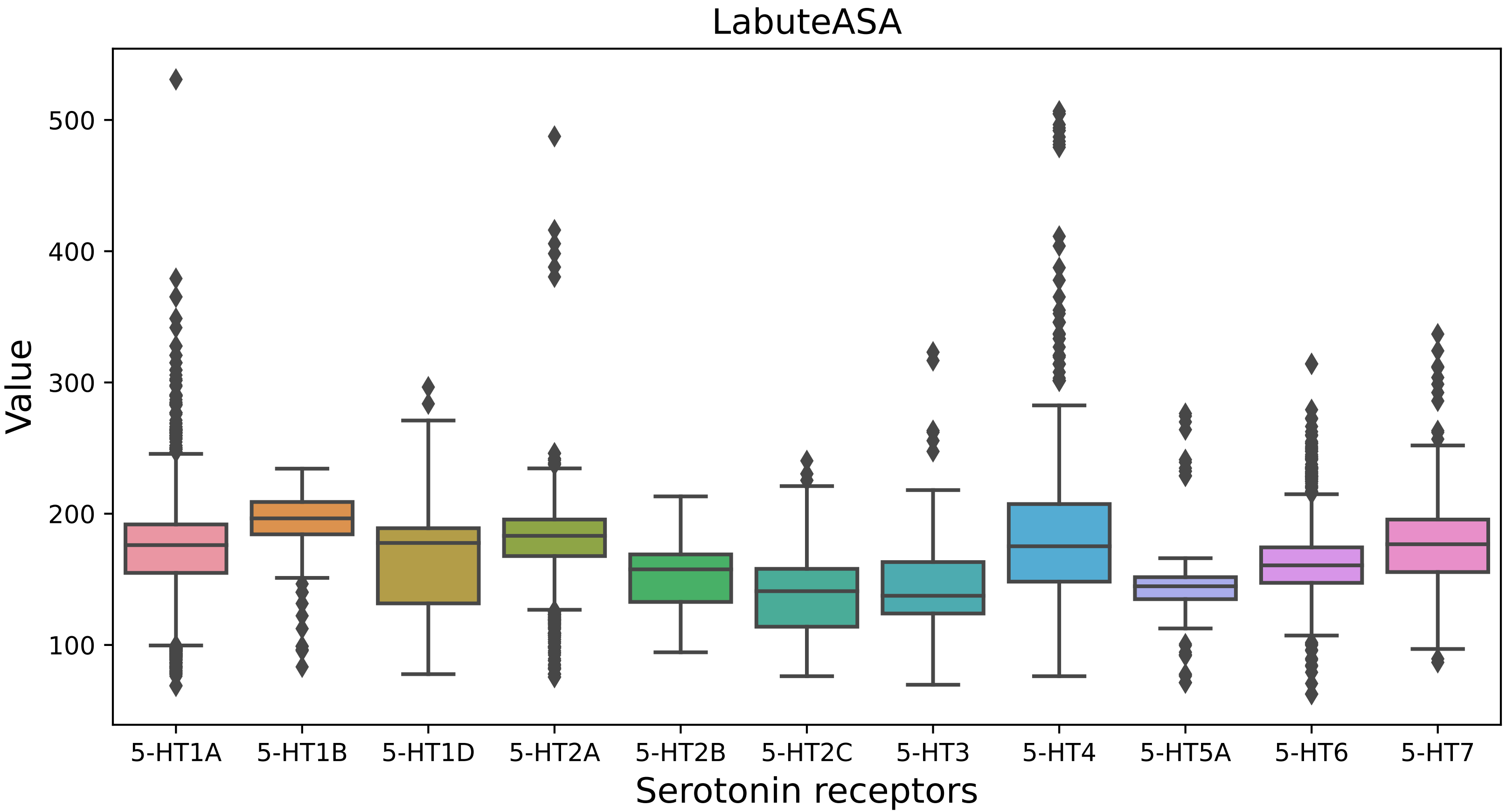
LabuteASA
Value
500
400
300
200
100
5-HT1A
5-HT1B
5-HT1D
5-HT2A
5-HT2B
5-HT2C
5-HT3
5-HT4
5-HT5A
5-HT6
5-HT7
Serotonin receptors

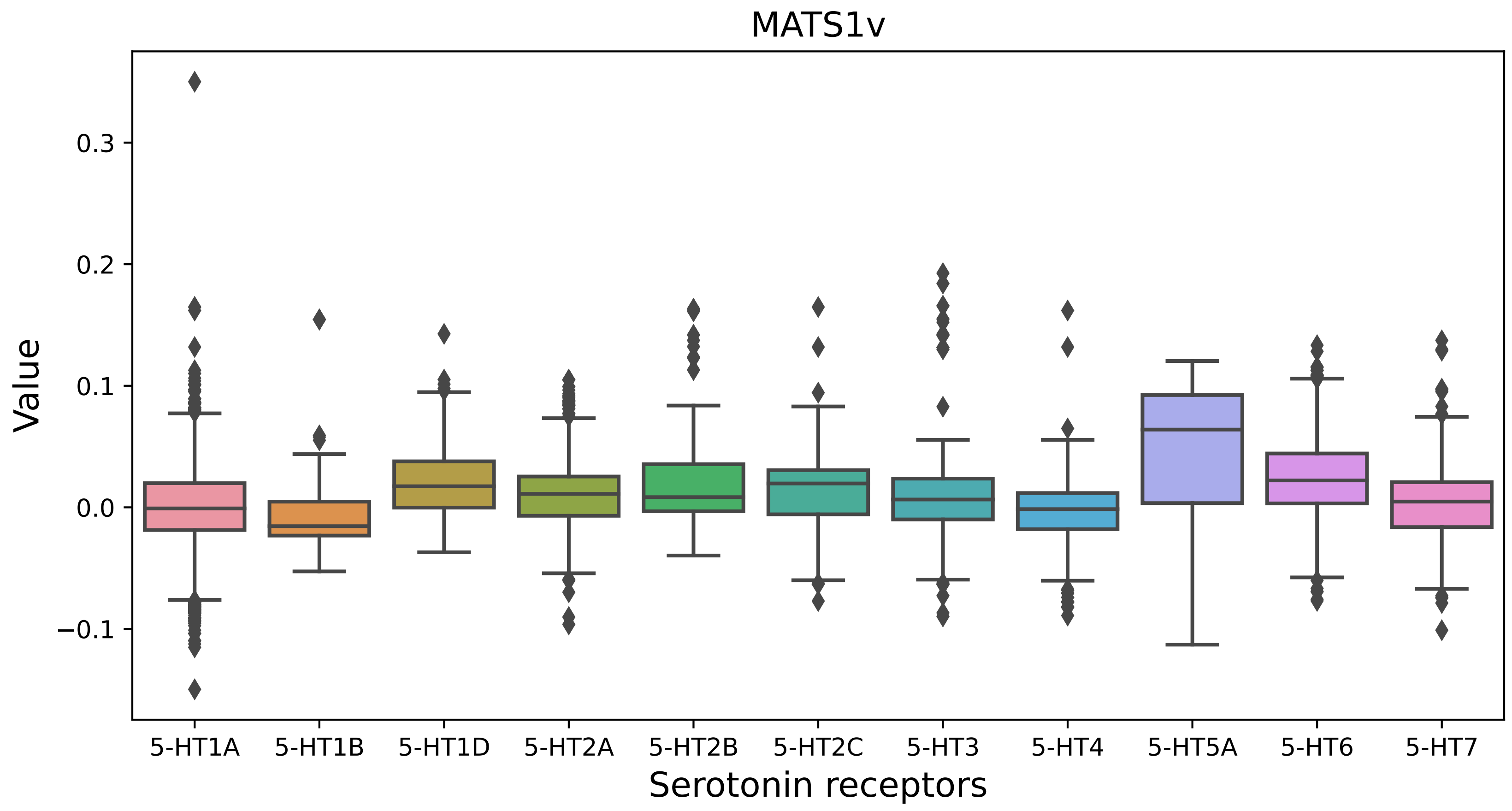

MATS1v
Value
0.3
0.2
0.1
0.0
−0.1
5-HT1A
5-HT1B
5-HT1D
5-HT2A
5-HT2B
5-HT2C
5-HT3
5-HT4
5-HT5A
5-HT6
5-HT7
Serotonin receptors

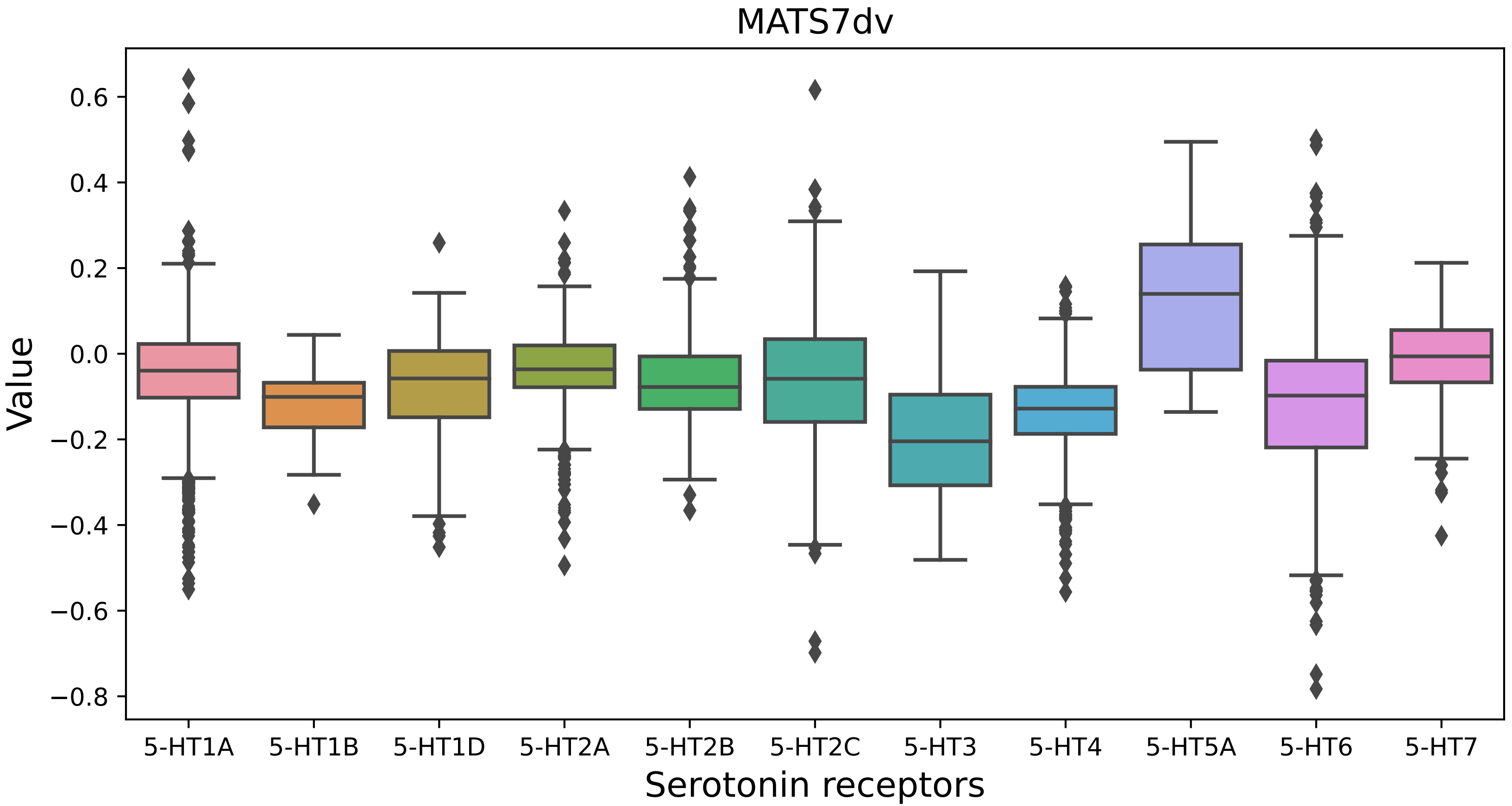

MATS7dv
0.6
0.4
0.2
0.0
−0.2
−0.4
−0.6
−0.8
Value
5-HT1A
5-HT1B
5-HT1D
5-HT2A
5-HT2B
5-HT2C
5-HT3
5-HT4
5-HT5A
5-HT6
5-HT7
Serotonin receptors

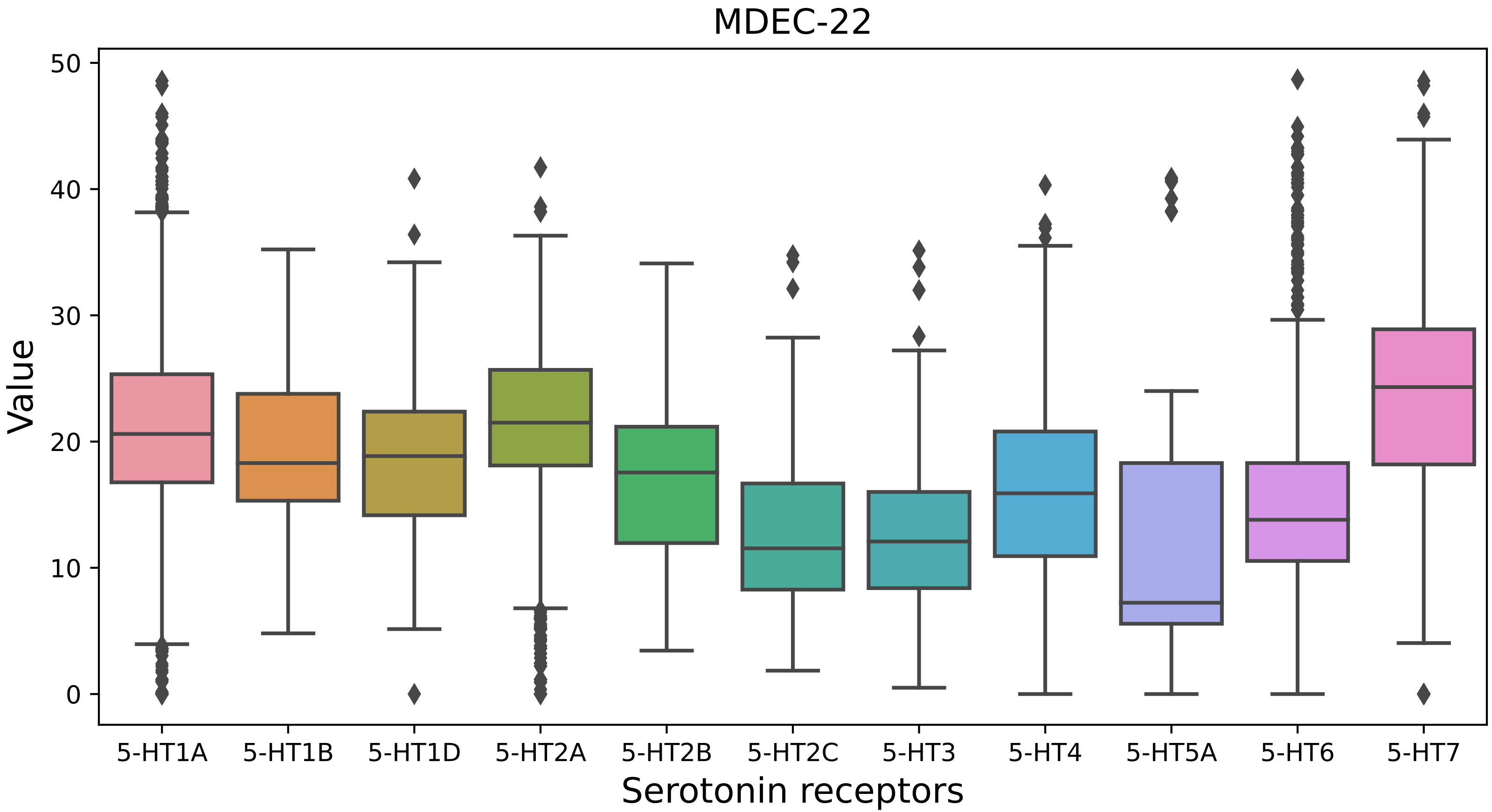
MDEC-22
Value
0
10
20
30
40
50
5-HT1A
5-HT1B
5-HT1D
5-HT2A
5-HT2B
5-HT2C
5-HT3
5-HT4
5-HT5A
5-HT6
5-HT7
Serotonin receptors

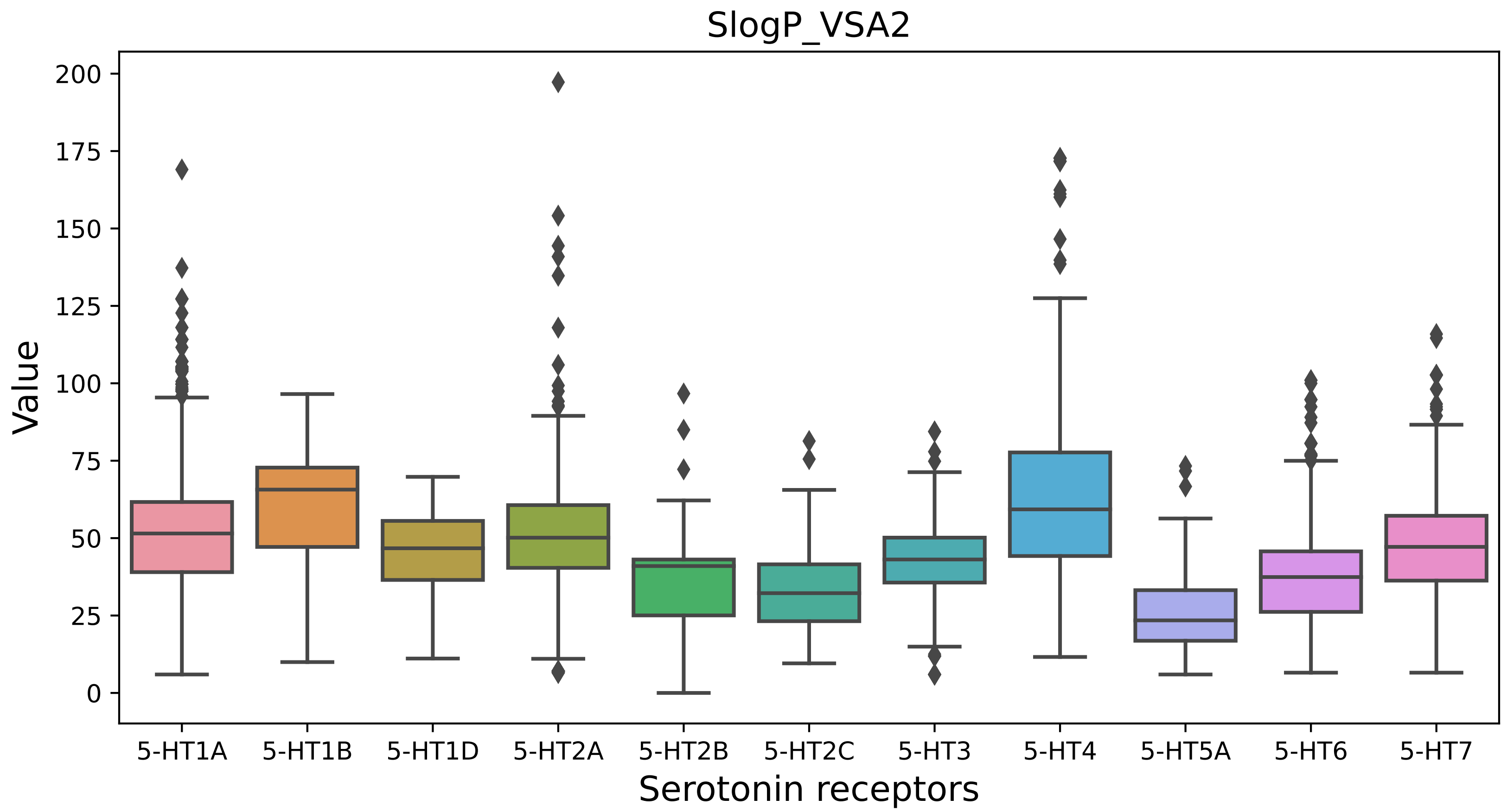
SlogP_VSA2
Value
200
175
150
125
100
75
50
25
0
5-HT1A
5-HT1B
5-HT1D
5-HT2A
5-HT2B
5-HT2C
5-HT3
5-HT4
5-HT5A
5-HT6
5-HT7
Serotonin receptors

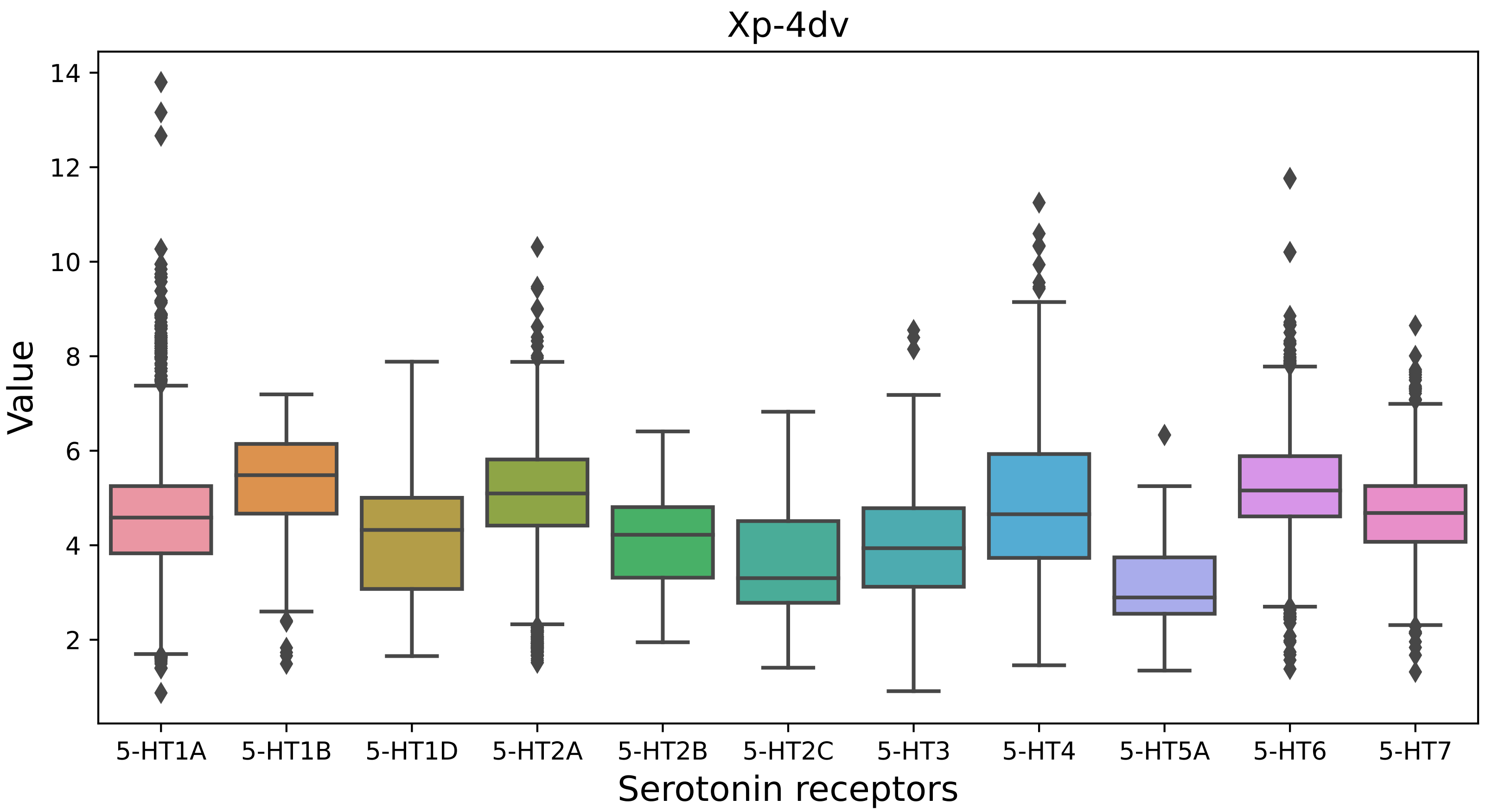
Xp-4dv
Value
14
12
10
8
6
4
2
5-HT1A
5-HT1B
5-HT1D
5-HT2A
5-HT2B
5-HT2C
5-HT3
5-HT4
5-HT5A
5-HT6
5-HT7
Serotonin receptors

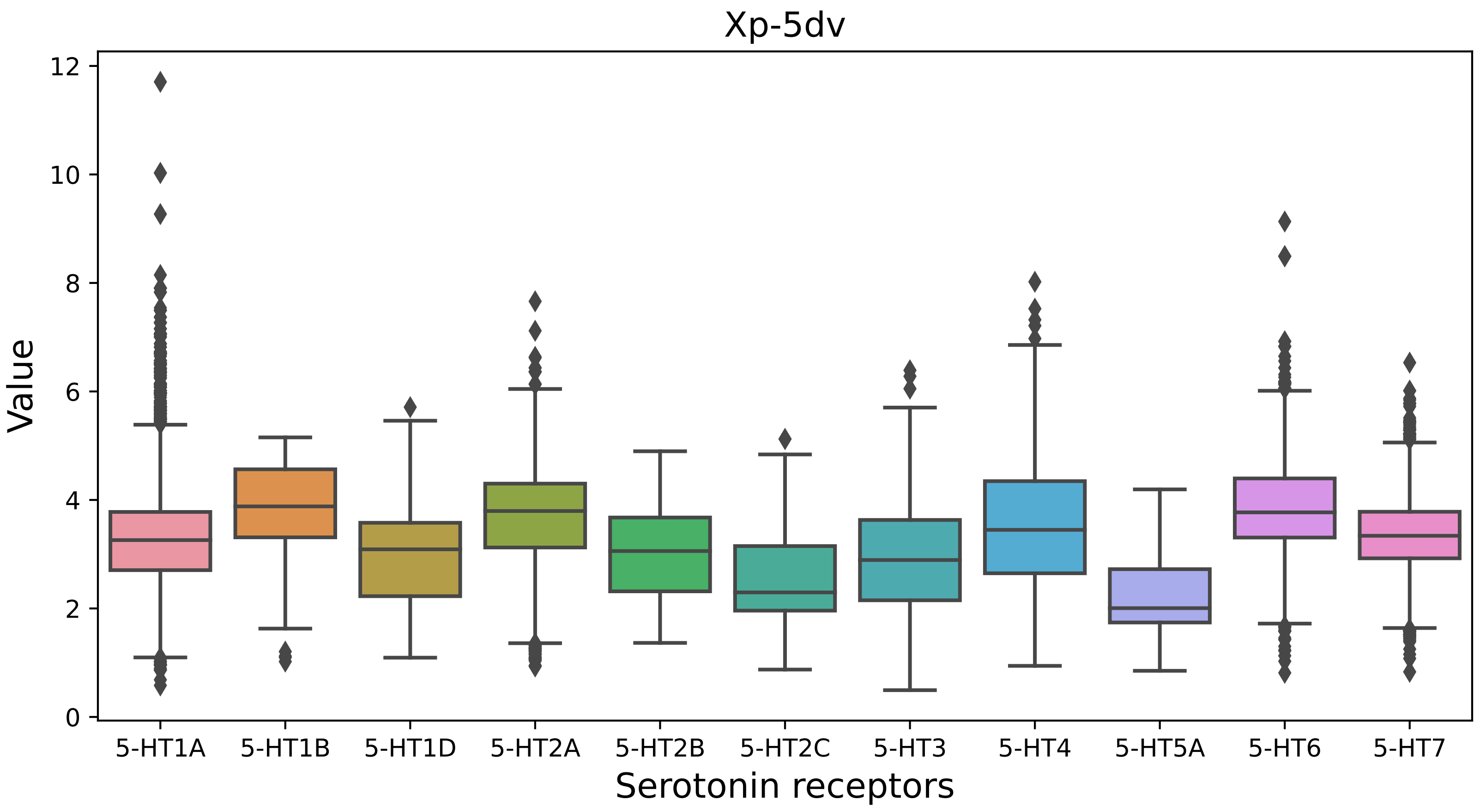

Xp-5dv
Value
12
10
8
6
4
2
0
5-HT1A
5-HT1B
5-HT1D
5-HT2A
5-HT2B
5-HT2C
5-HT3
5-HT4
5-HT5A
5-HT6
5-HT7
Serotonin receptors
