# Supplementary material for: Integrated QSAR Models for Prediction of Serotonergic Activity: Machine Learning Unveiling Activity and Selectivity Patterns of Molecular Descriptors
Source: Pharmaceutics. 2024 Mar 1;16(3):349. doi: 10.3390/pharmaceutics16030349 (PMC10974160; doi:10.3390/pharmaceutics16030349)
Supplement: Supplementary file 1 [file pharmaceutics-16-00349-s001.zip › Supplementary S3.pdf]

Supplementary S3: Value range of the most important descriptors for serotonergic activity model based on entire database

Descriptor	Active	Inactive
**nBase**	0 - 5	0 - 5
**MATS1v**	-0.15 - 0.35	-0.134 - 0.202
**PEOE_VSA7**	0.0 - 167.025	0.0 - 131.574
**SlogP_VSA1**	0.0 - 91.375	0.0 - 47.525
**AXp-7dv**	0.004 - 0.068	0.004 - 0.057
**PEOE_VSA9**	0.0 - 137.285	0.0 - 163.712
**Xch-7dv**	0.0 - 8.247	0.0 - 10.333
**AATSC2dv**	-0.662 - 2.213	-0.76 - 2.582
**VSA_EState2**	-1.306 - 87.06	-0.47 - 69.287
**ATSC6v**	-3489.32 - 3956.086	-3283.871 - 2862.563
**SLogP**	-3.24 - 9.445	-2.226 - 10.693
**Kier2**	2.939 - 41.981	2.146 - 44.684
**ATSC5d**	-101.306 - 22.712	-32.539 - 29.463
**PEOE_VSA1**	0.0 - 77.268	0.0 - 76.116
**JGI2**	0.031 - 0.144	0.028 - 0.156
**SpMAD_Dzare**	3.74 - 31.57	3.418 - 26.437
**SaasC**	-3.852 - 12.205	-4.633 - 14.035
**MATS1se**	-0.377 - 0.416	-0.396 - 0.459
**IC3**	2.969 - 6.109	3.303 - 6.127
**SaaaC**	-0.56 - 6.184	-0.699 - 6.396
**VSA_EState7**	-10.188 - 28.721	-10.783 - 22.081
**ATSC7Z**	-1023.77 - 709.752	-936.333 - 506.879
**ZMIC3**	15.555 - 88.704	15.542 - 108.155
**JGI9**	0.0 - 0.027	0.0 - 0.026
**nFRing**	0 - 3	0 - 3
**Kier3**	1.249 - 29.045	0.517 - 29.018
**SsssN**	0.0 - 13.116	0.0 - 10.038
**GATS4i**	0.429 - 1.433	0.54 - 1.536
**PEOE_VSA6**	0.0 - 110.967	0.0 - 130.584
**MAXaasC**	-0.572 - 1.709	-0.206 - 1.682
**GGI9**	0.0 - 1.413	0.0 - 1.084
**GATS6v**	0.382 - 1.925	0.111 - 1.628
**GATS3i**	0.553 - 2.01	0.564 - 1.633
**TopoShapeIndex**	0.0 - 1.0	0.0 - 1.0
**SssO**	0.0 - 77.604	0.0 - 79.125
**PEOE_VSA10**	0.0 - 60.131	0.0 - 62.373
**GATS3v**	0.294 - 1.999	0.693 - 1.442
