# Supplementary material for: Integrated QSAR Models for Prediction of Serotonergic Activity: Machine Learning Unveiling Activity and Selectivity Patterns of Molecular Descriptors
Source: Pharmaceutics. 2024 Mar 1;16(3):349. doi: 10.3390/pharmaceutics16030349 (PMC10974160; doi:10.3390/pharmaceutics16030349)
Supplement: Supplementary file 1 [file pharmaceutics-16-00349-s001.zip › Supplementary S4.pdf]

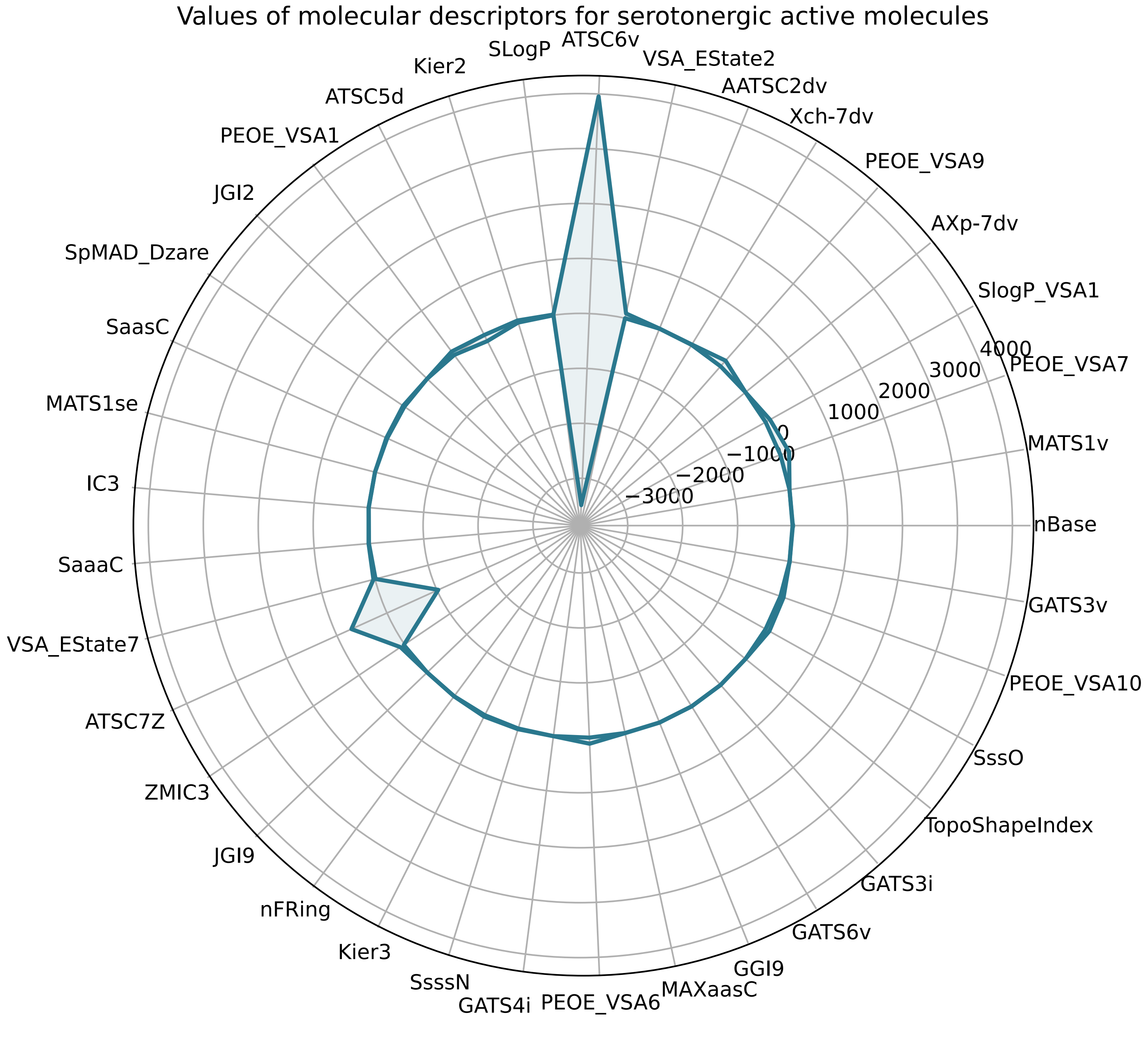

Values of molecular descriptors for serotonergic active molecules
ATSC6v
VSA_EState2
AATSC2dv
Xch-7dv
PEOE_VSA9
AXp-7dv
SlogP_VSA1
PEOE_VSA7
MATS1v
nBase
GATS3v
PEOE_VSA10
SssO
TopoShapeIndex
GATS3i
GATS6v
GGI9
MAXaasC
PEOE_VSA6
GATS4i
SsssN
Kier3
nFRing
JGI9
ZMIC3
ATSC7Z
VSA_EState7
SaaaC
IC3
MATS1se
SaasC
SpMAD_Dzare
JGI2
PEOE_VSA1
ATSC5d
Kier2
SLogP
4000
3000
2000
1000
0
−1000
−2000
−3000

Values of molecular descriptors for serotonergic active molecules

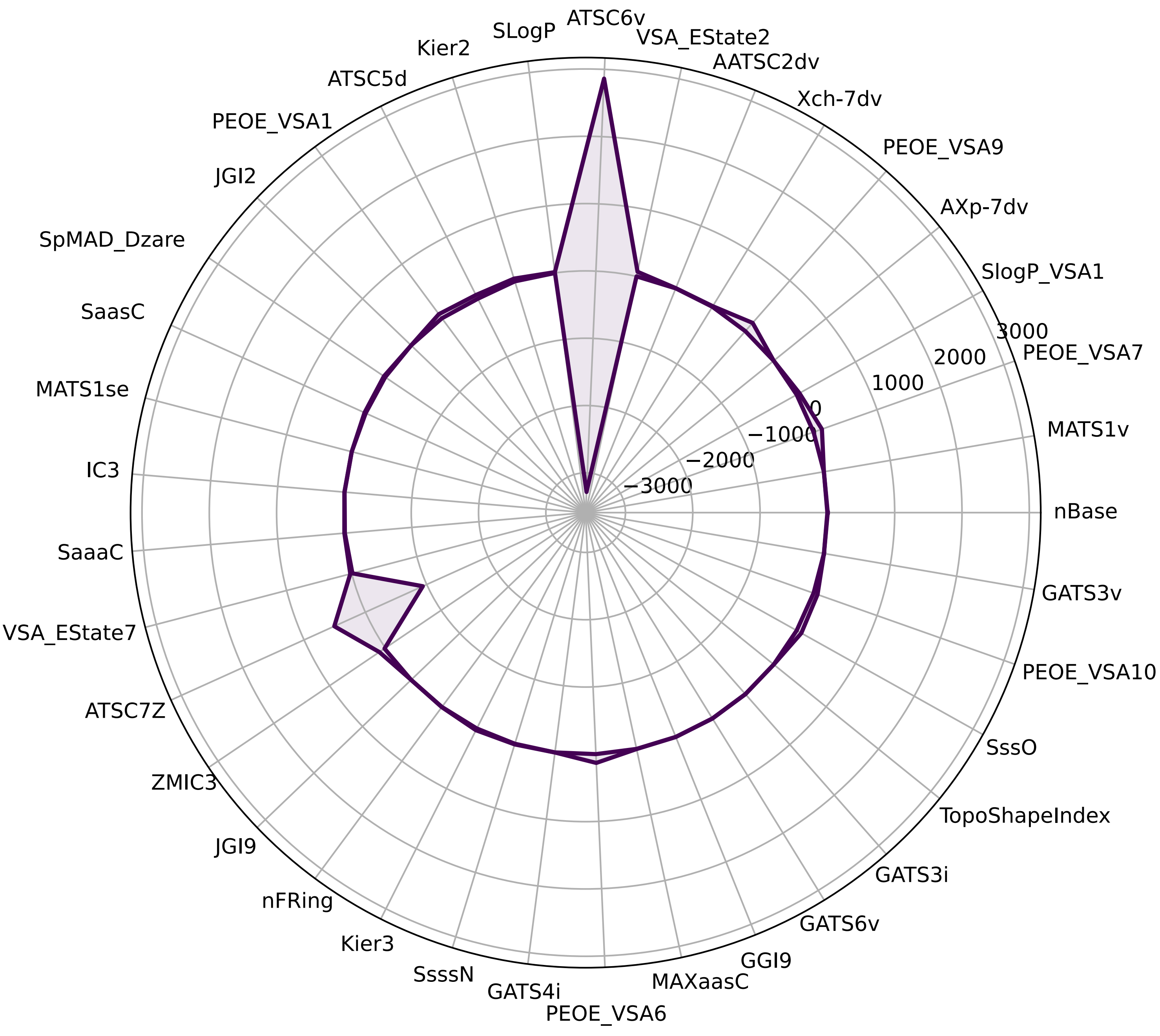

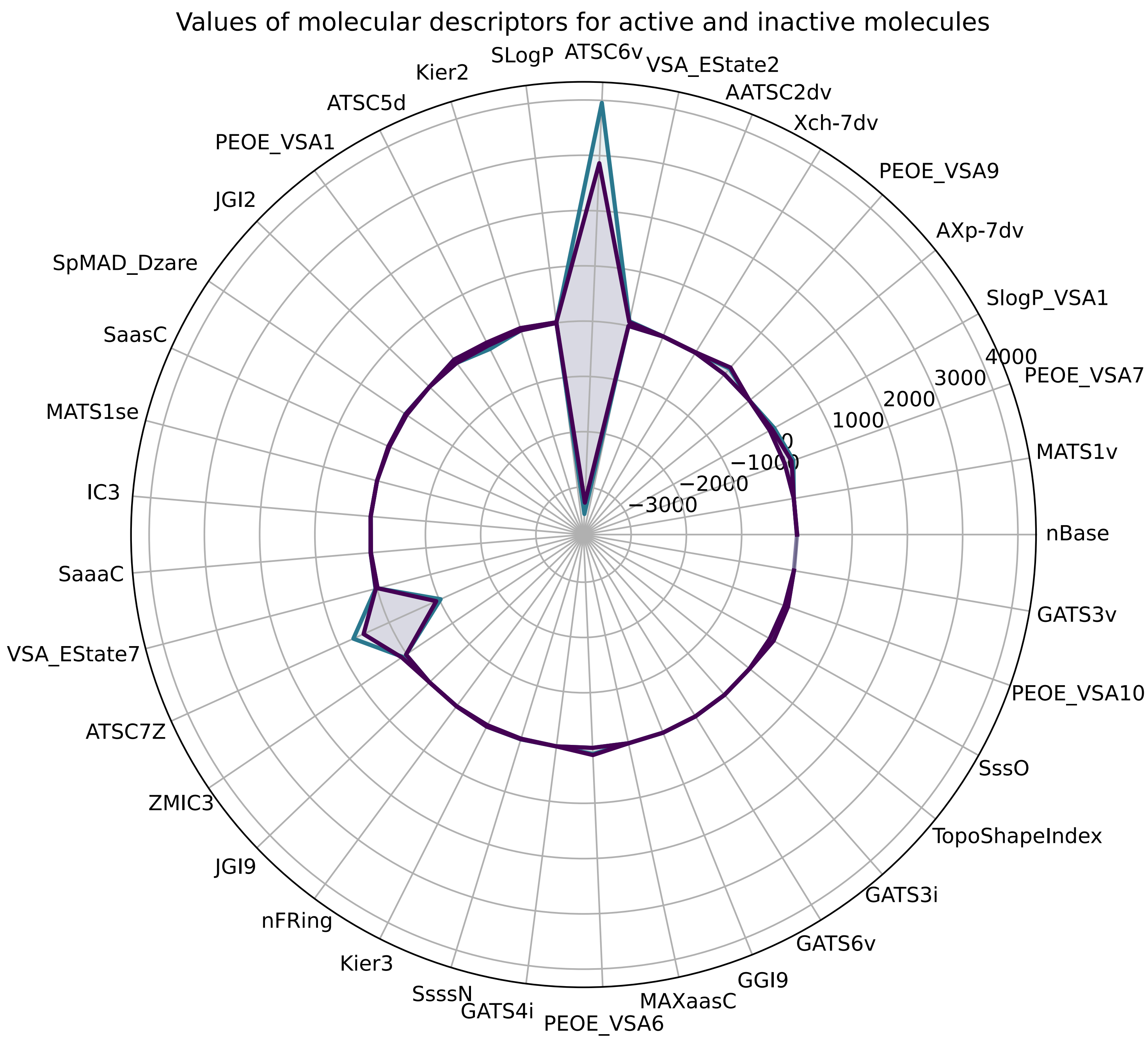
Values of molecular descriptors for active and inactive molecules
ATSC6v
VSA_EState2
AATSC2dv
Xch-7dv
PEOE_VSA9
AXp-7dv
SlogP_VSA1
PEOE_VSA7
MATS1v
nBase
GATS3v
PEOE_VSA10
SssO
TopoShapeIndex
GATS3i
GATS6v
GGI9
MAXaasC
PEOE_VSA6
GATS4i
SsssN
Kier3
nFRing
JGI9
ZMIC3
ATSC7Z
VSA_EState7
SaaaC
IC3
MATS1se
SaasC
SpMAD_Dzare
JGI2
PEOE_VSA1
ATSC5d
Kier2
SLogP
4000
3000
2000
1000
0
−1000
−2000
−3000

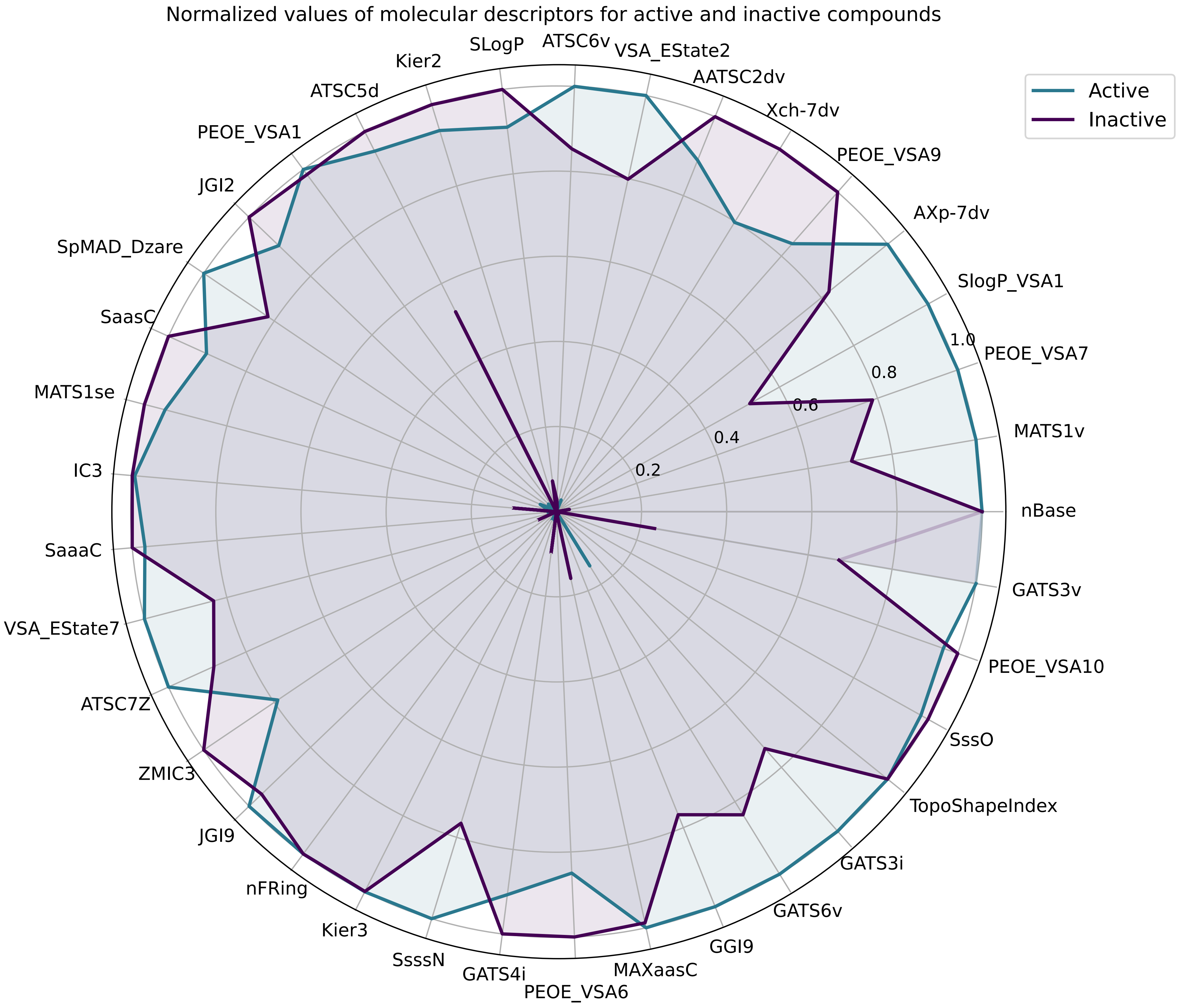

Normalized values of molecular descriptors for active and inactive compounds
Active
Inactive
nBase
MATS1v
PEOE_VSA7
SlogP_VSA1
AXp-7dv
PEOE_VSA9
Xch-7dv
AATSC2dv
VSA_EState2
ATSC6v
SLogP
Kier2
ATSC5d
PEOE_VSA1
JGI2
SpMAD_Dzare
SaasC
MATS1se
IC3
SaaaC
VSA_EState7
ATSC7Z
ZMIC3
JGI9
nFRing
Kier3
SsssN
GATS4i
PEOE_VSA6
MAXaasC
GGI9
GATS6v
GATS3i
TopoShapeIndex
SssO
PEOE_VSA10
GATS3v
0.2
0.4
0.6
0.8
1.0
