# Supplementary material for: Integrated QSAR Models for Prediction of Serotonergic Activity: Machine Learning Unveiling Activity and Selectivity Patterns of Molecular Descriptors
Source: Pharmaceutics. 2024 Mar 1;16(3):349. doi: 10.3390/pharmaceutics16030349 (PMC10974160; doi:10.3390/pharmaceutics16030349)
Supplement: Supplementary file 1 [file pharmaceutics-16-00349-s001.zip › Supplementary S5.pdf]

Supplementary S5: Value range of the most important descriptors for selectivity model of serotonin receptors based on entire database

**Descriptor**	**5-HT1A**	**5-HT1B**	**5-HT1D**	**5-HT2A**	**5-HT2B**	**5-HT2C**
SddssS	-8.625 - 0.0	-3.191 - 0.0	-5.656 - 0.0	-4.323 - 0.0	-3.792 - 0.0	-4.32 - 0.0
Xch-5d	0.0 - 1.659	0.0 - 0.341	0.0 - 0.415	0.0 - 1.093	0.0 - 1.023	0.0 - 0.463
AATSC2s	-0.141 - 1.841	0.03 - 0.238	-0.06 - 0.298	-0.081 - 1.76	0.004 - 1.447	-0.025 - 2.496
MDEC-33	0.0 - 27.181	2.381 – 14.992	1.438 - 13.542	0.007 - 17.306	1.651 - 15.141	0.005 - 14.487
ETA_dPsi_B	0.0 - 0.037	0.0 - 0.0	0.0 - 0.0	0.0 - 0.0	0.0 - 0.0	0.0 - 0.0
AATS6s	1.455 - 5.987	1.941 - 2.949	1.936 - 3.226	1.772 - 4.991	1.927 - 4.059	1.325 - 5.256
SpMAD_DzZ	3.821 - 21.871	5.366 - 11.034	5.102 - 12.879	4.264 - 16.145	5.275 - 12.048	4.325 - 13.636
ATSC3c	-0.53 - 1.57	-0.248 - 0.329	-0.332 - 0.363	-0.657 - 1.153	-0.949 - 0.28	-0.597 - 0.768
NsssCH	0-19	0-3	0-4	0-6	0-5	0-9
SaasN	-0.025 - 6.623	0.0 - 4.224	0.0 - 2.339	0.0 - 4.493	0.0 - 2.274	0.0 - 4.037
BalabanJ	0.0 - 3.251	0.925 - 1.876	0.0 - 2.077	0.0 - 2.601	1.013 - 2.06	0.0 - 2.12
NddssS	0 - 2	0 - 1	0 - 1	0 - 1	0 - 1	0 - 1
Xch-5dv	0.0 - 1.637	0.0 - 0.179	0.0 - 0.21	0.0 - 0.824	0.0 - 0.749	0.0 - 0.321

**Descriptor**	**5-HT3**	**5-HT4**	**5-HT5A**	**5-HT6**	**5-HT7**
SddssS	-3.729 - 0.0	-7.322 - 0.0	-3.521 - 0.0	-11.182 - 0.0	-3.927 - 0.0
Xch-5d	0.0 - 0.332	0.0 - 0.856	0.0 - 0.585	0.0 - 0.437	0.0 - 0.51
AATSC2s	-0.007 - 0.304	-0.025 - 1.203	-0.037 - 0.545	-0.052 - 2.032	-0.05 - 2.484
MDEC-33	0.5 - 25.061	0.5 - 17.618	0.071 - 20.353	0.001 - 18.67	0.002 - 17.799
ETA_dPsi_B	0.0 - 0.0	0.0 - 0.0	0.0 - 0.0	0.0 - 0.0	0.0 - 0.0
AATS6s	2.137 - 3.108	1.726 - 4.37	1.78 - 4.447	1.204 - 8.658	1.604 - 4.661
SpMAD_DzZ	5.863 - 10.055	6.529 - 32.06	3.825 - 10.055	4.128 - 19.549	6.207 - 25.19
ATSC3c	-1.137 - 0.5	-0.88 - 0.423	-0.881 - 0.644	-0.932 - 0.726	-0.855 - 0.899
NsssCH	0 - 5	0 - 7	0 - 2	0 - 6	0 - 5
SaasN	0.0 - 2.406	0.0 - 3.084	0.0 - 2.501	0.0 - 4.42	0.0 - 6.585
BalabanJ	0.784 - 2.562	0.0 - 2.454	0.0 - 2.025	0.0 - 2.169	0.0 - 2.263
NddssS	0 - 1	0 - 2	0 - 1	0 - 3	0 - 1
Xch-5dv	0.0 - 0.153	0.0 - 0.812	0.0 - 0.448	0.0 - 0.311	0.0 - 0.433
