# Supplementary material for: Integrated QSAR Models for Prediction of Serotonergic Activity: Machine Learning Unveiling Activity and Selectivity Patterns of Molecular Descriptors
Source: Pharmaceutics. 2024 Mar 1;16(3):349. doi: 10.3390/pharmaceutics16030349 (PMC10974160; doi:10.3390/pharmaceutics16030349)
Supplement: Supplementary file 1 [file pharmaceutics-16-00349-s001.zip › Supplementary S6.pdf]

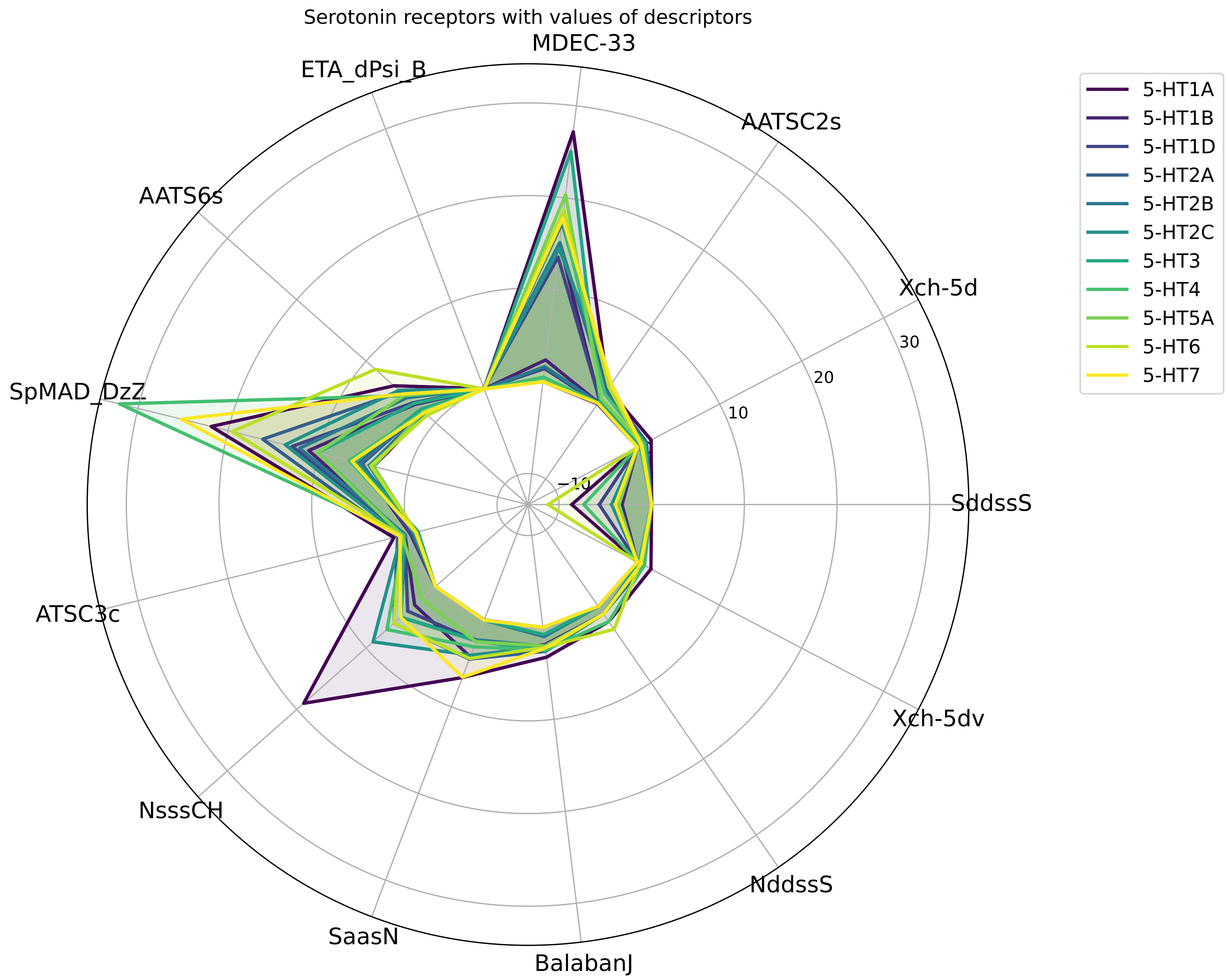
Serotonin receptors with values of descriptors
MDEC-33
AATSC2s
Xch-5d
SddssS
Xch-5dv
NddssS
BalabanJ
SaasN
NsssCH
ATSC3c
SpMAD_DzZ
AATS6s
ETA_dPsi_B
−10
10
20
30
5-HT1A
5-HT1B
5-HT1D
5-HT2A
5-HT2B
5-HT2C
5-HT3
5-HT4
5-HT5A
5-HT6
5-HT7

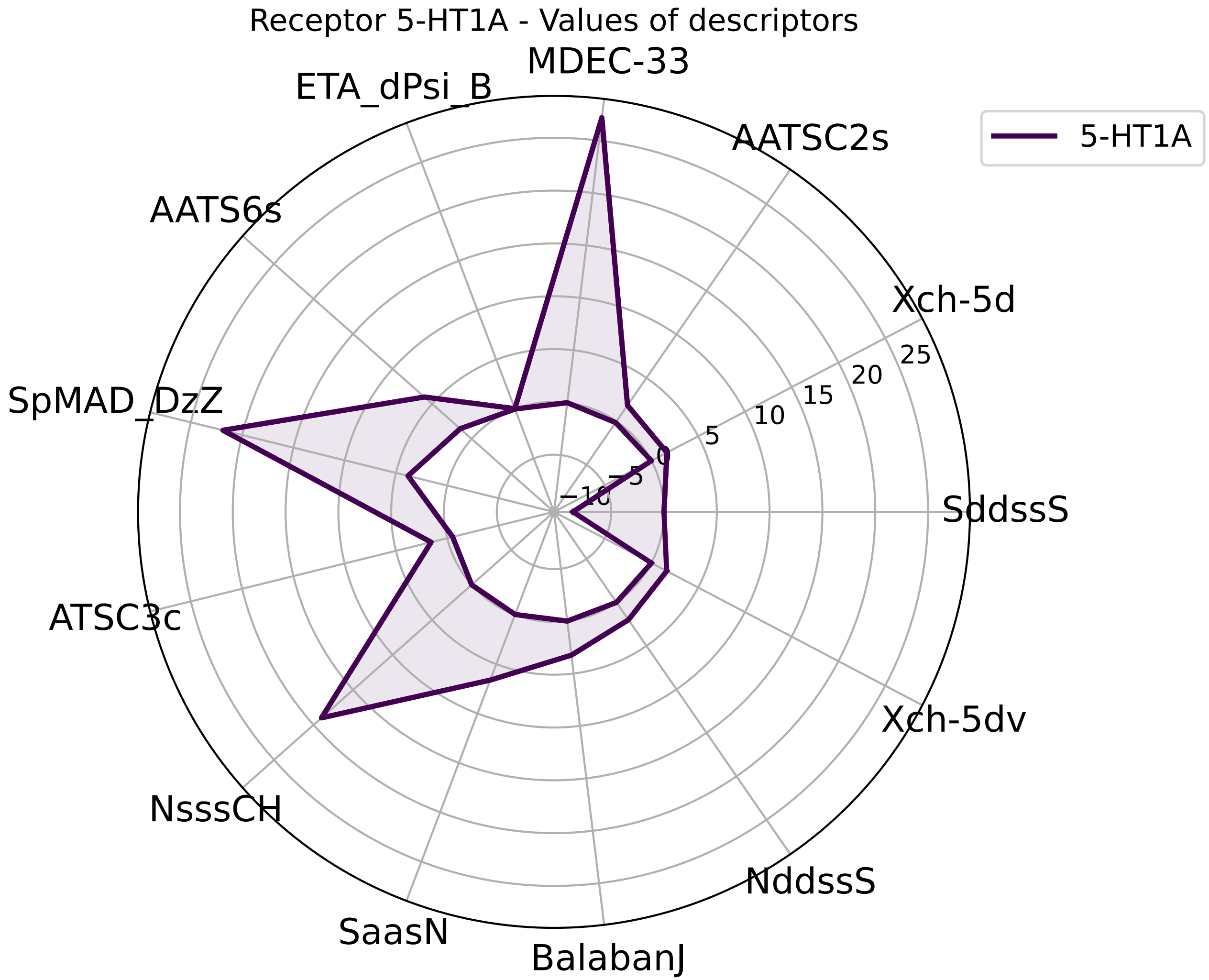

Receptor 5-HT1A - Values of descriptors
MDEC-33
AATSC2s
Xch-5d
SddssS
Xch-5dv
NddssS
BalabanJ
SaasN
NsssCH
ATSC3c
SpMAD_DzZ
AATS6s
ETA_dPsi_B
5-HT1A
−10
−5
0
5
10
15
20
25

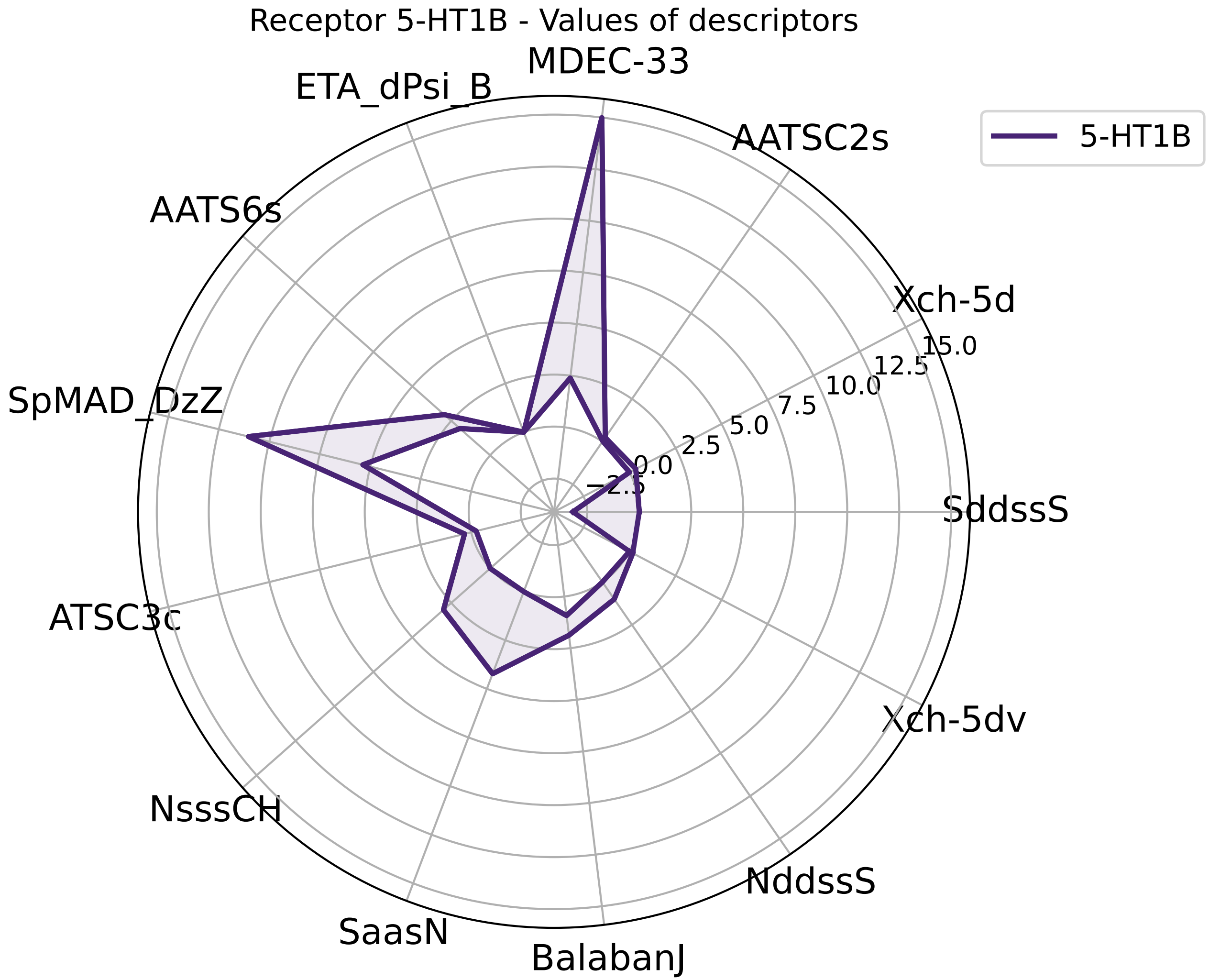

Receptor 5-HT1B - Values of descriptors
MDEC-33
AATSC2s
Xch-5d
SddssS
Xch-5dv
NddssS
BalabanJ
SaasN
NsssCH
ATSC3c
SpMAD_DzZ
AATS6s
ETA_dPsi_B
5-HT1B
−2.5
0.0
2.5
5.0
7.5
10.0
12.5
15.0

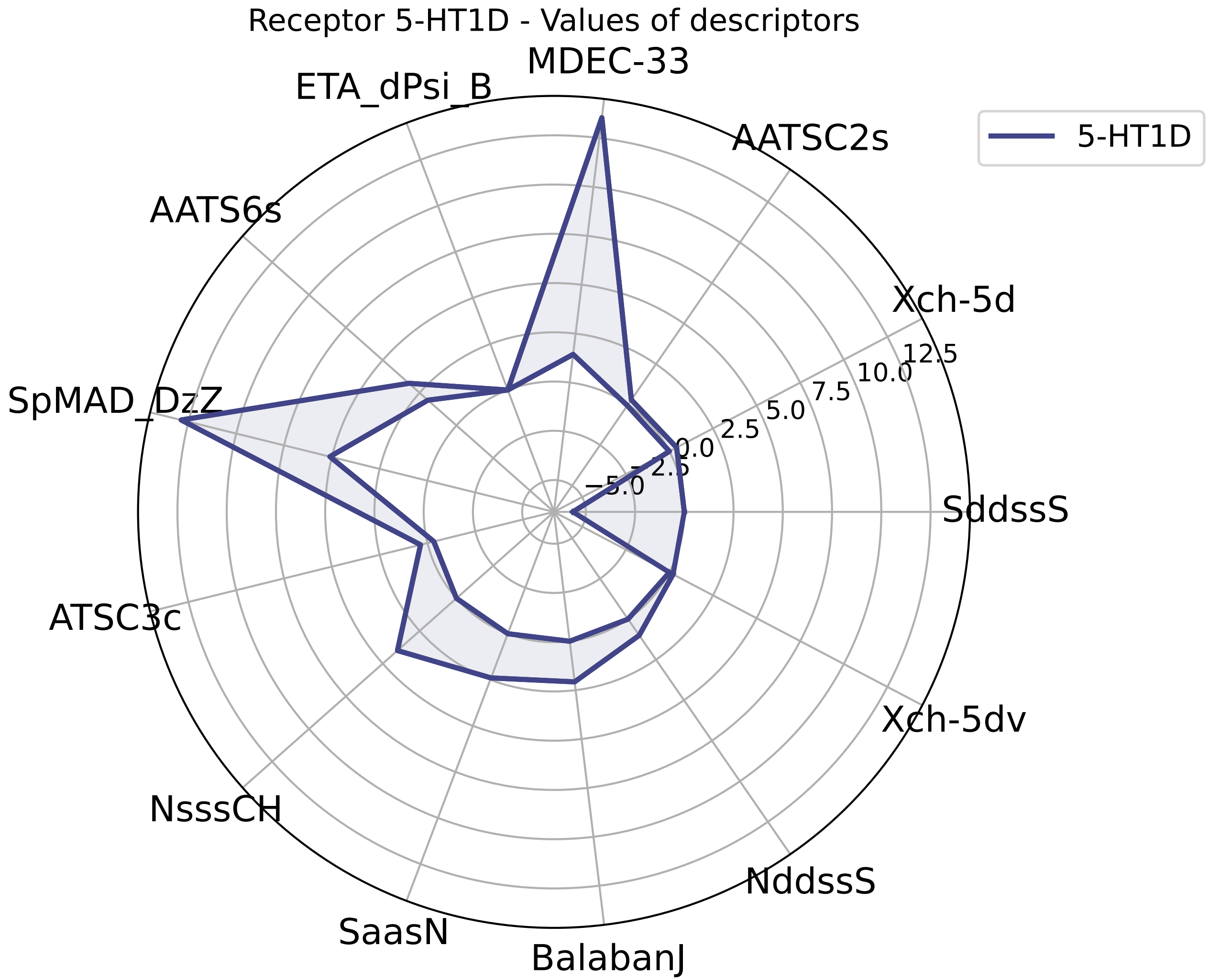

Receptor 5-HT1D - Values of descriptors
MDEC-33
AATSC2s
Xch-5d
SddssS
Xch-5dv
NddssS
BalabanJ
SaasN
NsssCH
ATSC3c
SpMAD_DzZ
AATS6s
ETA_dPsi_B
5-HT1D
12.5
10.0
7.5
5.0
2.5
0.0
−2.5
−5.0

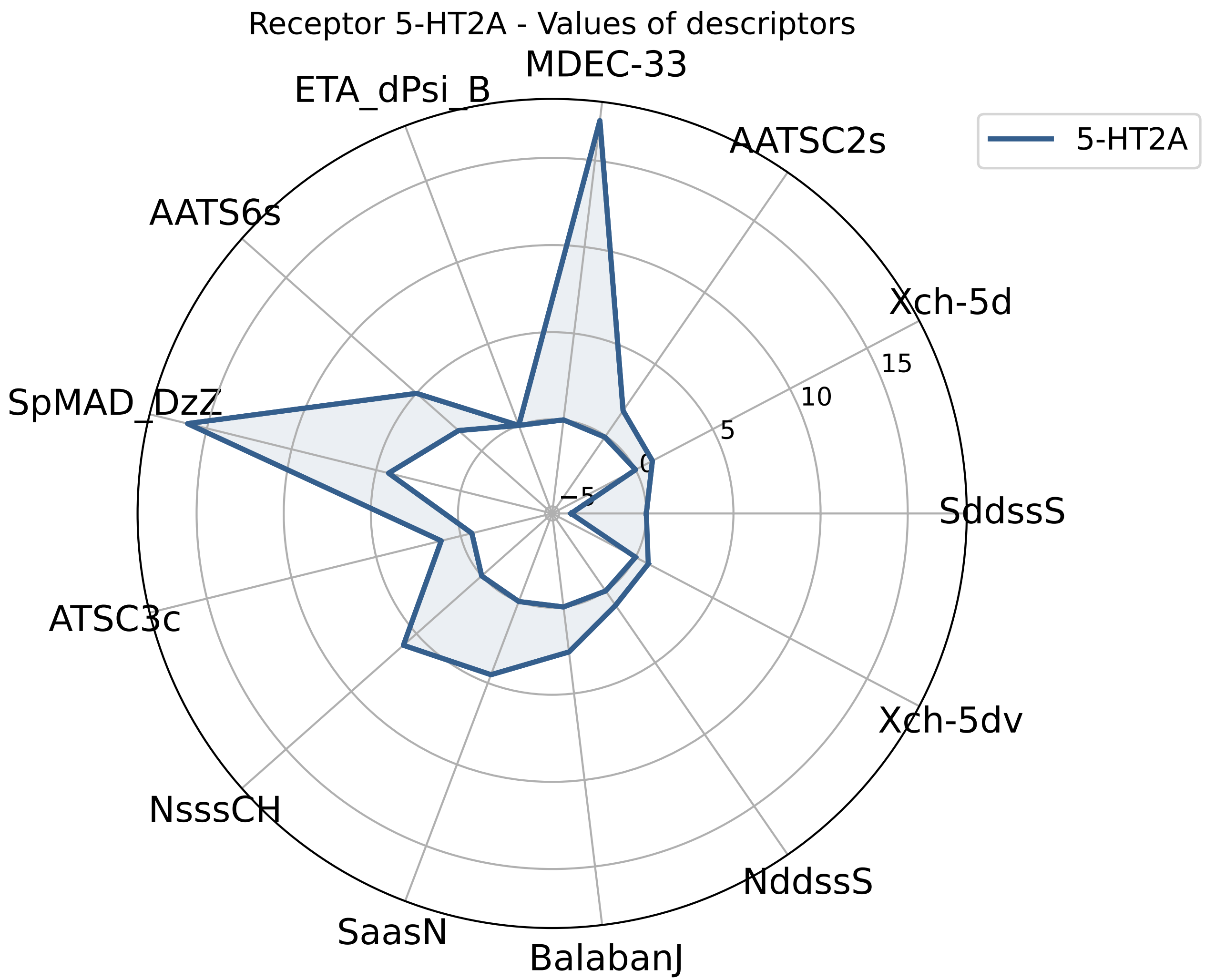

Receptor 5-HT2A - Values of descriptors
MDEC-33
AATSC2s
Xch-5d
SddssS
Xch-5dv
NddssS
BalabanJ
SaasN
NsssCH
ATSC3c
SpMAD_DzZ
AATS6s
ETA_dPsi_B
−5
0
5
10
15
5-HT2A

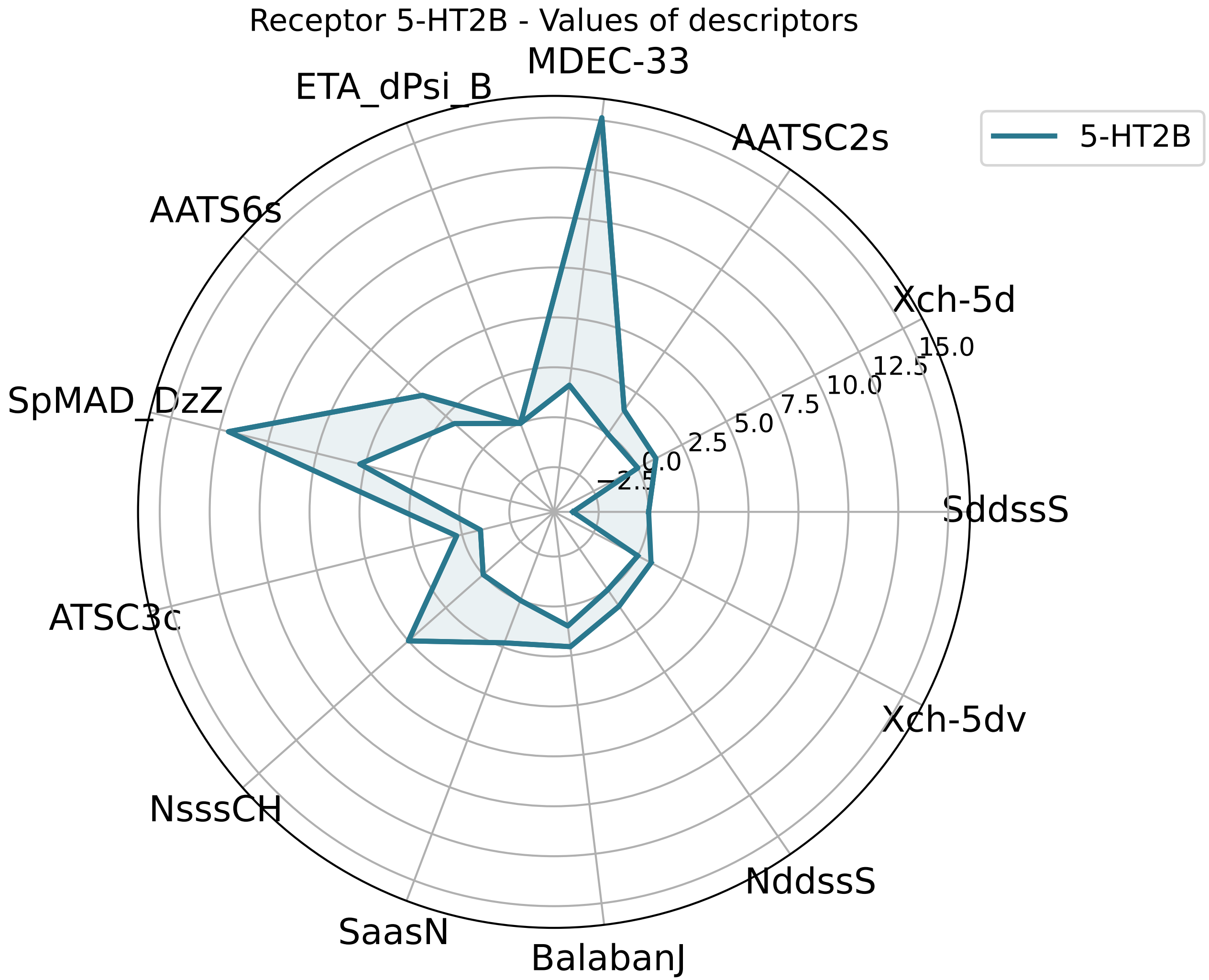
Receptor 5-HT2B - Values of descriptors
MDEC-33
AATSC2s
Xch-5d
SddssS
Xch-5dv
NddssS
BalabanJ
SaasN
NsssCH
ATSC3c
SpMAD_DzZ
AATS6s
ETA_dPsi_B
5-HT2B
15.0
12.5
10.0
7.5
5.0
2.5
0.0
−2.5

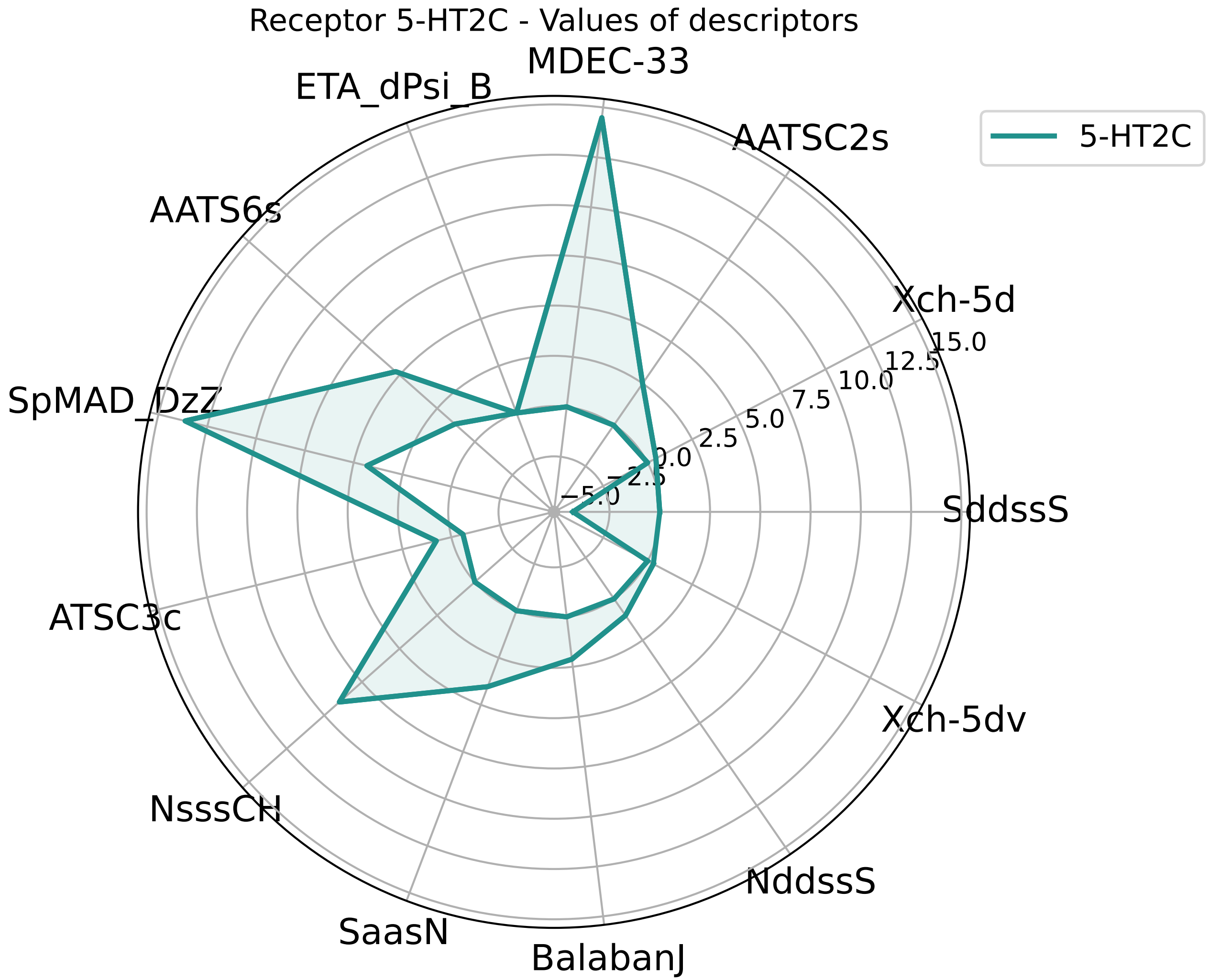
Receptor 5-HT2C - Values of descriptors
MDEC-33
AATSC2s
Xch-5d
SddssS
Xch-5dv
NddssS
BalabanJ
SaasN
NsssCH
ATSC3c
SpMAD_DzZ
AATS6s
ETA_dPsi_B
5-HT2C
−5.0
−2.5
0.0
2.5
5.0
7.5
10.0
12.5
15.0

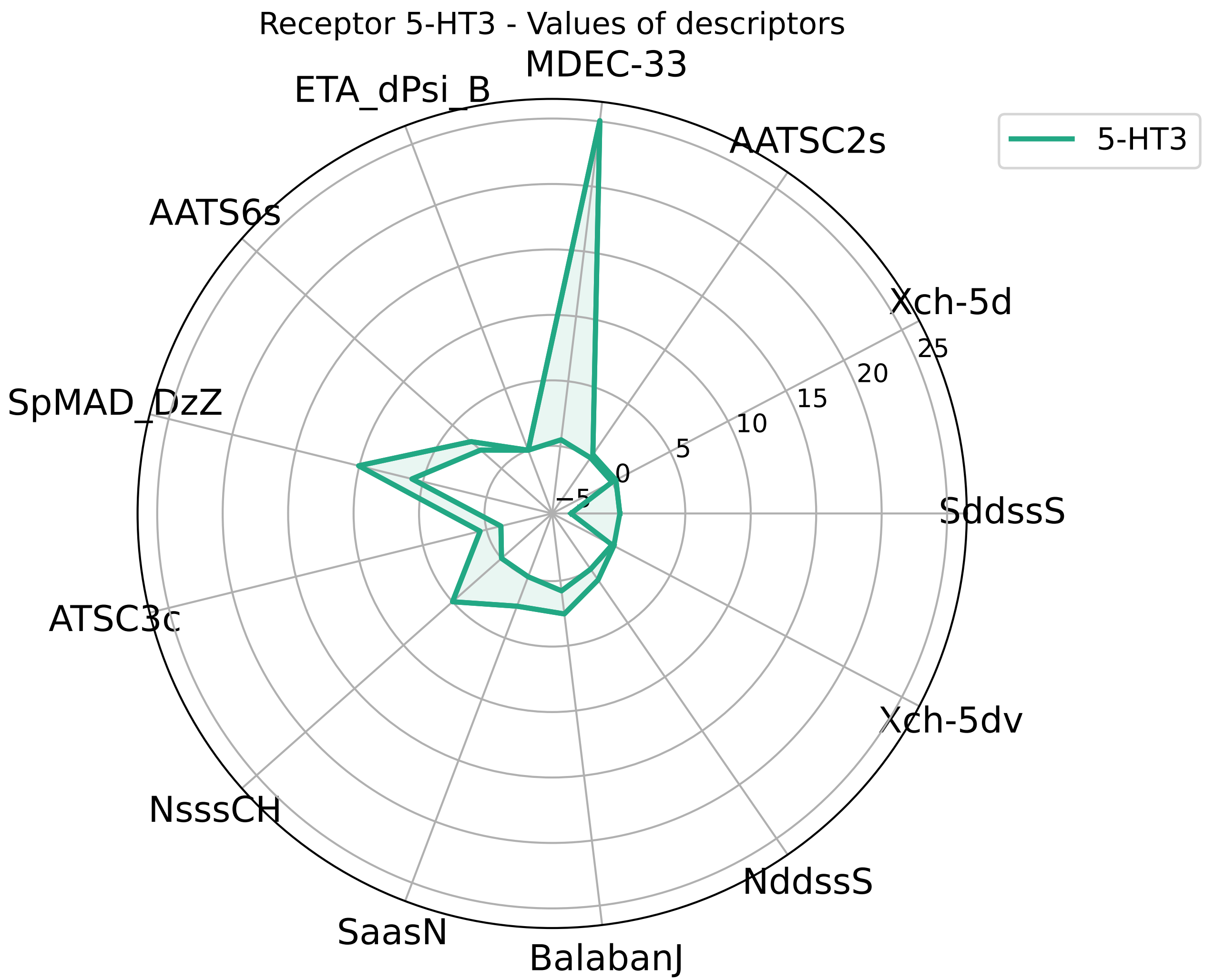

Receptor 5-HT3 - Values of descriptors
MDEC-33
AATSC2s
Xch-5d
SddssS
Xch-5dv
NddssS
BalabanJ
SaasN
NsssCH
ATSC3c
SpMAD_DzZ
AATS6s
ETA_dPsi_B
−5
0
5
10
15
20
25
5-HT3

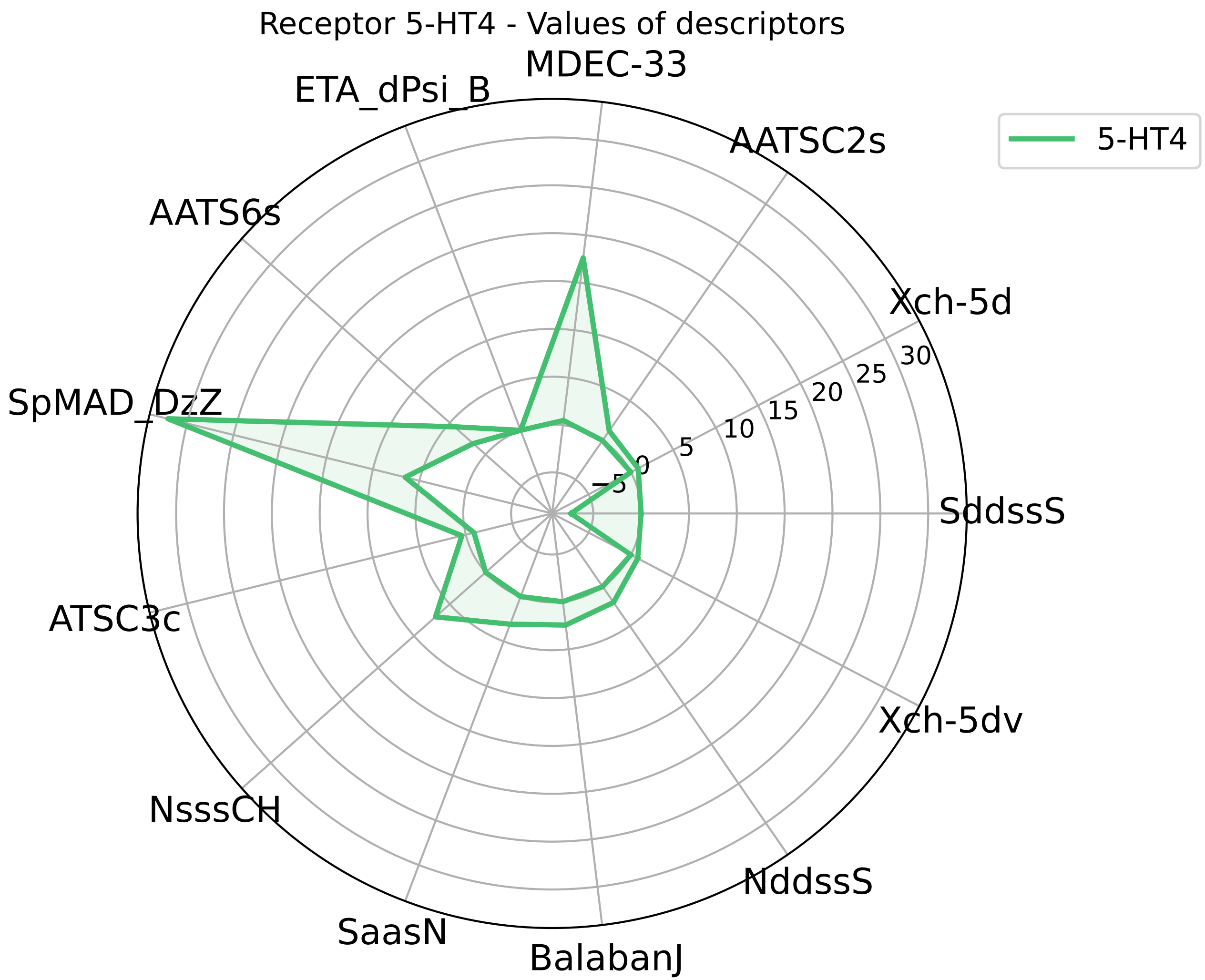

Receptor 5-HT4 - Values of descriptors
MDEC-33
AATSC2s
Xch-5d
SddssS
Xch-5dv
NddssS
BalabanJ
SaasN
NsssCH
ATSC3c
SpMAD_DzZ
AATS6s
ETA_dPsi_B
5-HT4
−5
0
5
10
15
20
25
30

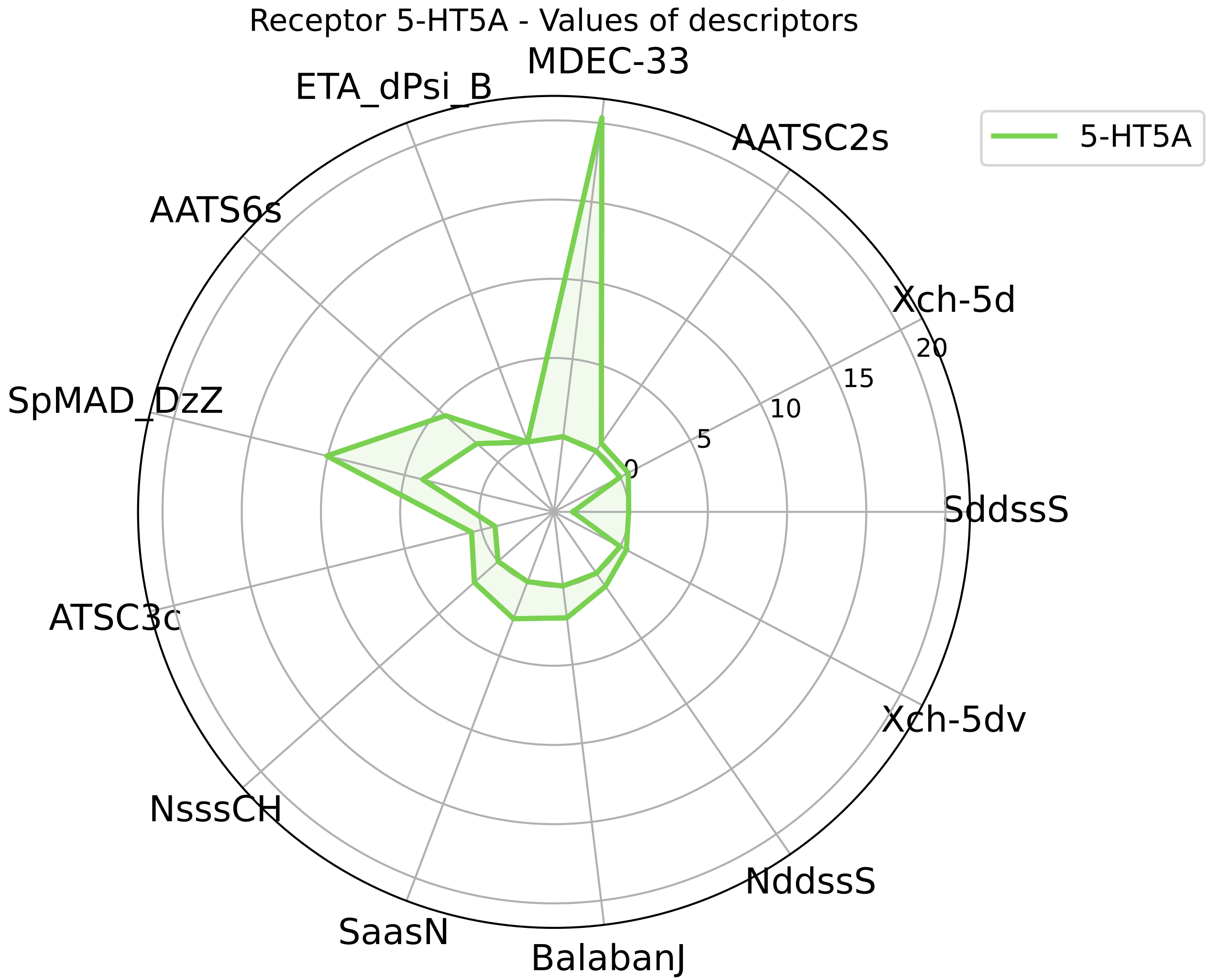

Receptor 5-HT5A - Values of descriptors
MDEC-33
AATSC2s
Xch-5d
SddssS
Xch-5dv
NddssS
BalabanJ
SaasN
NsssCH
ATSC3c
SpMAD_DzZ
AATS6s
ETA_dPsi_B
0
5
10
15
20
5-HT5A

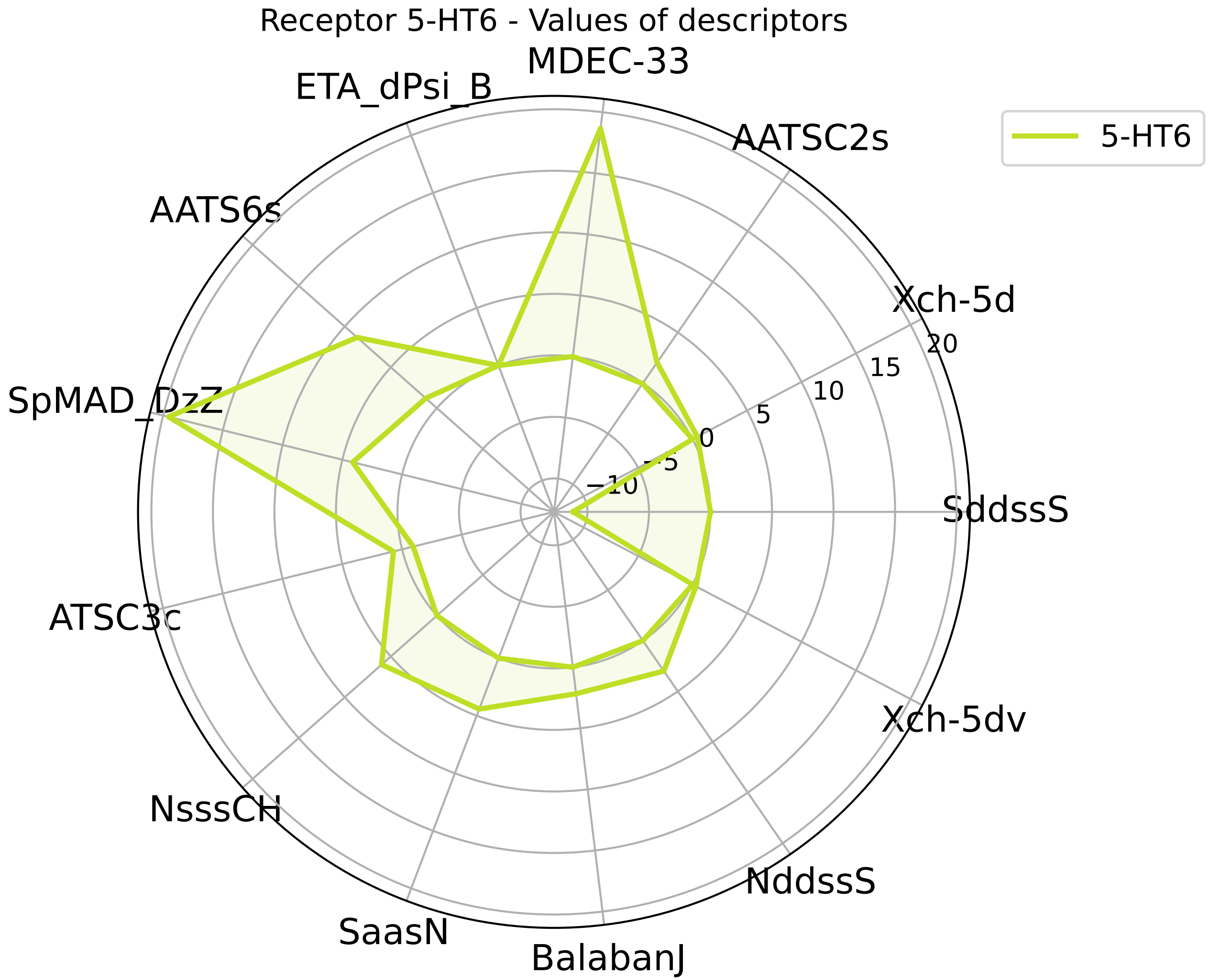

Receptor 5-HT6 - Values of descriptors
MDEC-33
AATSC2s
Xch-5d
SddssS
Xch-5dv
NddssS
BalabanJ
SaasN
NsssCH
ATSC3c
SpMAD_DzZ
AATS6s
ETA_dPsi_B
20
15
10
5
0
−5
−10
5-HT6

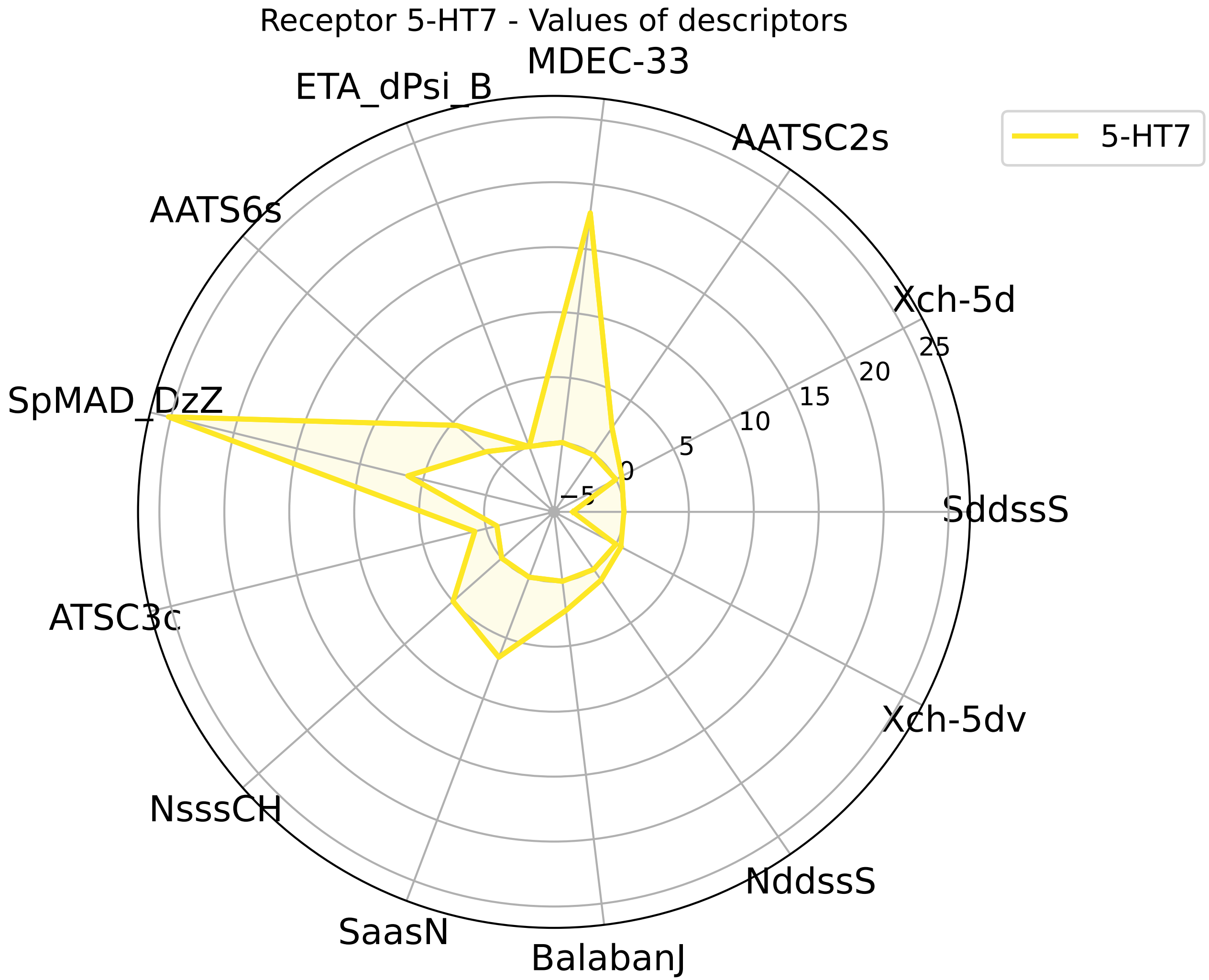

Receptor 5-HT7 - Values of descriptors
MDEC-33
AATSC2s
Xch-5d
SddssS
Xch-5dv
NddssS
BalabanJ
SaasN
NsssCH
ATSC3c
SpMAD_DzZ
AATS6s
ETA_dPsi_B
−5
0
5
10
15
20
25
5-HT7
